# Deeper Analysis to Identify the True Benefit of ICIs Immunotherapy in First-Line Treatment for Non-HER2-Positive/HER2-Negative Advanced or Metastatic Advanced or Metastatic Gastric Cancer (GC) or Gastroesophageal Junction Cancer (GEJC)

**DOI:** 10.3390/cancers17040657

**Published:** 2025-02-15

**Authors:** Bowen Zheng, Fanzhuoran Lou, Yuting He, Miao Fu, Xintian Huang, Weijuan Tan, Quan Chen, Xiaowen Xie, Tianhui Hu, Li Xiao

**Affiliations:** 1 Department of Oncology, Zhongshan Hospital of Xiamen University, School of Medicine, Xiamen University, Xiamen 361004, China; 24520220157288@stu.xmu.edu.cn (B.Z.); 24520231154759@stu.xmu.edu.cn (F.L.); 24520221154629@stu.xmu.edu.cn (Y.H.); 24520241154932@stu.xmu.edu.cn (X.H.); tanwj102@163.com (W.T.); cq498348619@163.com (Q.C.); 2 National Institute for Data Science in Health and Medicine, Xiamen University, Xiamen 361102, China; 3 Xiamen Key Laboratory for Tumor Metastasis, Cancer Research Center, School of Medicine, Xiamen University, Xiamen 361102, China; 24520231154559@stu.xmu.edu.cn (M.F.); 24520241154893@stu.xmu.edu.cn (X.X.); 4 Shenzhen Research Institute of Xiamen University, Shenzhen 518057, China

**Keywords:** gastric cancer, gastroesophageal junction cancer, immune checkpoint inhibitors, positive programmed death ligand 1

## Abstract

**Purpose:** Immune checkpoint inhibitors (ICIs) have improved survival outcomes in patients with advanced or metastatic gastric cancer (GC) or gastroesophageal junction cancer (GEJC) in the first-line setting. However, who will truly benefit from ICIs based on PD-L1 expression in different segments remains controversial, lacking high-level evidence. **Methods:** We performed a pooled analysis of several approved phase 3 trials as first-line immunotherapy for non-HER2-positive/HER2-negative advanced or metastatic GC or gastroesophageal junction cancer (GEJC) based on PD-L1 expression in segments with different expression levels. Kaplan–Meier curves and hazard ratios for overall survival (OS) were extracted. KMSubtraction-derived survival data for unreported subgroups and one-stage pooled analyses were used to estimate the efficacy of the ICI regimens. The primary outcome was OS, and the secondary outcome was progression-free survival across different PD-L1-expression subgroups. **Results:** This study is the largest global-sample-size pooled analysis to date, which involved a total of 4807 patients, with 2407 (50.1%) in the ICI-plus-chemotherapy group and 2400 (49.9%) in the chemotherapy-only group. Overall, the evidence demonstrates that adding ICIs to chemotherapy may not provide additional OS or PFS benefits in the negative-PD-L1-expression (CPS < 1) or PD-L1-low subgroups (CPS 1–4/TAP < 5%) compared with chemotherapy alone. Patients with high PD-L1 CPS scores (CPS ≥ 10) had significant OS and PFS benefits, whereas patients with moderate PD-L1 CPS scores (CPS 5–9) showed unclear OS and PFS benefits. Our analysis indicated that first-line immunotherapy combined with induction chemotherapy, followed by maintenance therapy, may be more suitable for practical clinical applications than immunotherapy combined with continuous chemotherapy. **Conclusions:** These findings can provide stronger evidence to identify patients who truly benefit from first-line ICI immunotherapy based on PD-L1 expression in different segments and offer a basis for optimal treatment and research in advanced or metastatic GC or GEJC.

## Simple Summary

This study investigates the true benefit of immune checkpoint inhibitors (ICIs) in first-line treatment for advanced or metastatic gastric cancer (GC) and gastroesophageal junction cancer (GEJC) based on PD-L1 expression. By analyzing data from several phase 3 clinical trials, we found that ICIs combined with chemotherapy significantly improve overall survival (OS) and progression-free survival (PFS) in patients with high PD-L1 CPS scores (CPS ≥ 10). However, the benefits are less clear in patients with low or negative PD-L1 expression. Our findings suggest that first-line immunotherapy combined with induction chemotherapy followed by maintenance therapy may be more effective than continuous chemotherapy. This study provides valuable insights for optimizing treatment strategies and identifying patients who will most benefit from ICIs in clinical practice.

## 1. Introduction

Gastric cancer (GC) has a high global incidence and mortality rate [1,2]. Several immune checkpoint inhibitors (ICIs) have received approval as first-line immunotherapy for non-HER2-positive/HER2-negative advanced or metastatic GC or gastroesophageal junction cancer (GEJC) in several phase 3 trials, including CheckMate-649, KEYNOTE-859, ORIENT-16, RATIONALE-305, etc. [3,4,5,6,7].

Based on CheckMate-649, the U.S. Food and Drug Administration (FDA) approved nivolumab in combination with certain types of chemotherapy (XELOX (capecitabine and oxaliplatin) or FOLFOX (leucovorin, fuorouracil, and oxaliplatin)) for the initial treatment of patients with advanced or metastatic GC or GEJC (non-HER2-positive tumors). This was the first FDA-approved immunotherapy for the first-line treatment of gastric cancer. On the basis of KEYNOTE-859, the FDA approved pembrolizumab with fluoropyrimidine- and platinum-containing chemotherapy for the first-line treatment of adults with locally advanced unresectable or metastatic HER2-negative GC or GEJC [8,9]. On the basis of ORIENT-16, the National Medical Products Administration (NMPA) of China granted approval for a combined therapy of sintilimab in combination with certain types of chemotherapy (XELOX) for the initial treatment of patients with advanced or metastatic GC or GEJC (non-HER2-positive tumors) [5]. On the basis of RATIONALE-305, the NMPA approved a combined therapy of tislelizumab in combination with certain types of chemotherapy (XELOX or FP: 5-fuorouracil and cisplatin) for the initial treatment of patients with advanced or metastatic GC or GEJC (non-HER2-positive tumors) [6]. PD-L1 expression has been investigated as a predictive marker for PD-1 inhibitor response and efficacy. However, the potential benefit and efficacy of patients with a negative PD-L1 value (CPS < 1) or a low expression of it (CPS 1–4) remains uncertain and controversial [10].

The KMSubtraction method and the IPDfromKM (Individual Patient Data from Kaplan–Meier) method are both techniques for reconstructing individual patient data (IPD) from published Kaplan–Meier (KM) survival curves, each with distinct strengths and limitations. The IPDfromKM method is widely used and highly accurate, extracting IPD by identifying coordinates on KM curves and incorporating information on the number at risk [11]. In contrast, the KMSubtraction method focuses on extracting survival data from unreported subgroups by matching and excluding patients from the overall cohort and known subgroups [12]. While IPDfromKM excels in reconstructing overall data, KMSubtraction uniquely addresses unreported subgroup data, filling a gap in IPDfromKM [12]. Integrating these methods allows for the comprehensive utilization of published survival data, enhancing secondary analyses in clinical research, such as assessing treatment efficacy across different subgroups [13]. This integration is particularly valuable in secondary analyses of clinical studies, such as assessing the efficacy of specific treatments across different subgroups [14].

We conducted a KMSubtraction analysis of four large phase 3 RCTs that tested the addition of PD-1 inhibitors to chemotherapy programs in first-line advanced non-HER2-positive/HER2-negative advanced or metastatic GC or GEJC. All four studies (CheckMate-649, KEYNOTE-859, ORIENT-16, and RATIONALE-305) showed a statistically significant improvement in PFS and OS in all patients; however, the responses were clearly enriched in patients with PD-L1-positive tumors, using CPS scores of various metrics in the first three trials and TAP in the RATIONALE-305 study. However, no trial has attempted to assess the benefit of ICI addition in patients with tumors with a negative or low PD-L1 value. This was, in part, addressed by Zhao et al., although the studies (CheckMate-649, KEYNOTE-062, and KEYNOTE-590) analyzed in the current report are non-overlapping, except for CheckMate-649 and the additional trials included in our report, and are more recent and relevant to the ICI discussion in the first therapy of GC or GEJC [14]. Given the variability in study designs across the aforementioned trials, we categorized and analyzed studies with similar designs in pursuit of more objective conclusions in previously unreported PD-L1-expression subgroups.

## 2. Materials and Methods

This study followed the preferred reporting items for systematic reviews and meta-analyses (PRISMA) reporting guideline for individual patient data (IPD) [15]. Since real human subjects were not involved, approval by an institutional review board (IRB) or approval by an ethics committee was not required. And the protocol of this systematic review was registered with PROSPERO, the international prospective register of systematic reviews (CRD42025638030).

### 2.1. Study Selection

On 18 May 2024, we systematically searched the PubMed, Web of Science, EMBASE, and Cochrane Library databases using specific terms. We focused on randomized controlled trials (RCTs) of ICIs alone or with chemotherapy for advanced/metastatic GC or GEJC in non-HER2-positive/HER2-negative patients (Supplementary Table S1). The included trials were CheckMate-649, KEYNOTE-859, ORIENT-16, and RATIONALE-305. The analysis focused on Kaplan–Meier (KM) plots of the entire study population and PD-L1 CPS subgroups, excluding studies with a significant missing PD-L1 status to minimize biases.

### 2.2. PD-L1 IHC and Interpretation

PD-L1 levels in tumor tissues were assessed using the following specific assays: PD-L1 IHC 22C3 pharmDx in KEYNOTE-859 and ORIENT-16, 28–8 pharmDx in CheckMate-649, and the VENTANA SP263 IHC Assay in RATIONALE-305. CPS ranges from 0 to 100 and is calculated as the ratio of PD-L1-stained cells to viable tumor cells multiplied by 100. PD-L1 expression is categorized as negative (CPS < 1), low (CPS 1–4), moderate (CPS 5–9), and strong (CPS ≥ 10) [16]. TAP denotes the ratio of PD-L1-positive tumor cells and immune cells to the total tumor area, with expression defined as positive if present in ≥1% of the tumor or immune cells [17].

### 2.3. Risk-of-Bias Assessment and Time-to-Event Outcomes Reconstruction

Two reviewers independently evaluated the methodological quality of the included RCTs using the Cochrane Collaboration’s risk of bias (RoB 2) tool [18]. The method proposed by Guyot et al. was used to derive a close approximation of the original individual patient time-to-event outcomes from published KM survival curves [19,20,21].

### 2.4. KMSubtraction

The workflow of KMSubtraction entails the retrieval of unreported subgroup survival data from published KM plots. This method was initially utilized to obtain an unreported KM plot for the subgroup with low PD-L1 expression in advanced gastric and esophageal cancer (Supplementary Table S2) [14]. N. L et al. and J. J. Z et al. validated the effectiveness of KMSubtraction in extracting survival data from an unreported subpopulation [11,12]. The proportional hazards (PHs) regression model was utilized to compute the hazard ratio (HR) for KM plots of an undisclosed subgroup cohort. Next, the median absolute value of natural logarithmic-transformed hazard ratios (|ln(HR)|), obtained through a Monte Carlo simulation, was utilized to assess error limits, with |ln(HR)| equal to 0 representing the ideal state [14].

In summary, we utilized the Hungarian algorithm to align the overall and subgroup KM curves and identify unreported subgroups by excluding matched patients. For each implementation of KMSubtraction, a Monte Carlo simulation was employed to calculate the error limits across 5000 iterations [11].

### 2.5. One-Stage Pooled Analysis

Using derived individual patient data (IPD), one-stage pooled analyses were performed to estimate the efficacy of ICI-based regimens in subgroups with negative or low PD-L1 expressions. OS was the primary outcome, and PFS was the secondary outcome. In the shared-frailty model, a random-effects term was included to account for between-study heterogeneity. The frailties were gamma-distributed.

### 2.6. Statistical Analysis

Pooled hazard ratios (HRs) and corresponding 95% confidence intervals (CIs) were summarized using the generic inverse variance method. Pre-specified subgroup analyses were conducted at specific CPS thresholds (<1, ≥1, 1–4, <5, ≥5, 5–9, <10, and ≥10). Using the reconstructed IPD, cumulative survival probabilities were estimated using Kaplan–Meier curves, and differences were compared using the log-rank test. HRs with 95% CIs were calculated using an unadjusted Cox proportional hazard model. All analyses were performed in R (version 4.3.3) using the robvis, survival, KMSubtraction, IPDfromKM, survRM2, ggplot2, meta, and forestploter packages. Statistical significance was set at *p* < 0.05.

## 3. Results

### 3.1. Study Selection

**Figure 1:**
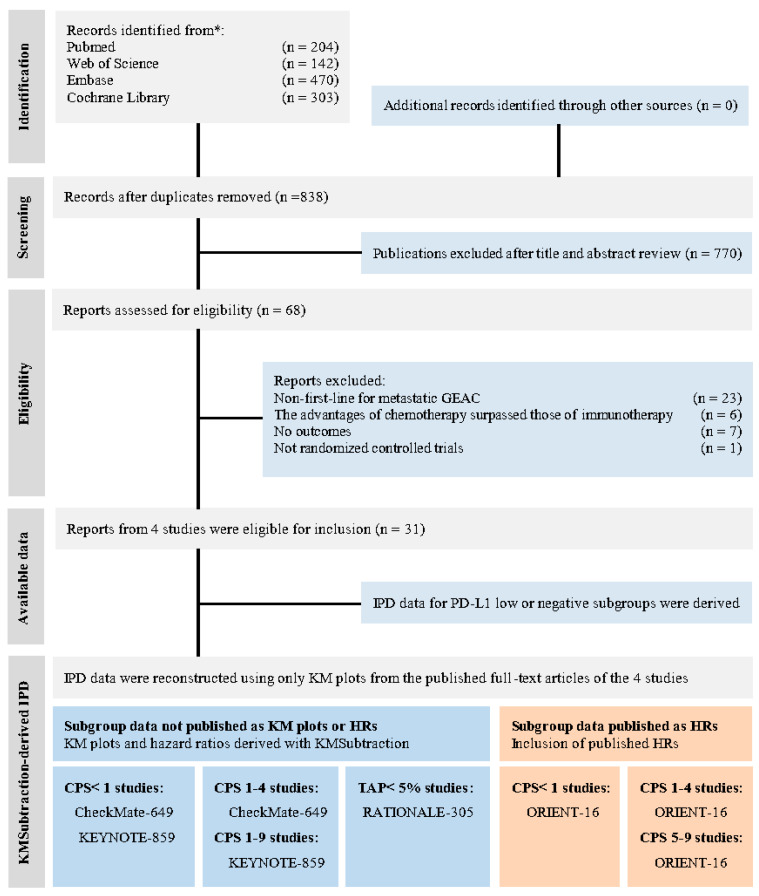
PRISMA diagram. PRISMA, preferred reporting items for systematic reviews and meta-analyses; PD-L1, programmed death ligand 1; CPS, combined positive score; TAP, tumor area positivity; IPD, individual patient data; KM, Kaplan–Meier; * Consider, if feasible to do so, reporting the number of records identified from each database or register searched (rather than the total number across all databases/registers).

Following electronic retrieval, four RCTs were included in this study after eliminating duplicate data and conducting rigorous screening (Figure 1). The search scope was expanded to comprehensively gather clinical trials that reported KM plots from a wide range of sources, including peer-reviewed journals, abstracts, conference presentations, and ongoing research.

### 3.2. Baseline Characteristics of Clinical Trials Included in This Analysis

CheckMate-649, ORIENT-16, and RATIONALE-305 excluded patients with HER2-positive tumors, whereas KEYNOTE-859 exclusively recruited patients with HER2-negative tumors. Our analysis involved a total of 4807 patients, with 2407 (50.1%) in the ICI-plus-chemotherapy group and 2400 (49.9%) in the chemotherapy-only group. CheckMate-649, KEYNOTE-859, and RATIONALE-305 recruited patients from different countries (referred to as global patients in this study), whereas ORIENT-16 exclusively recruited patients from China. Additionally, CheckMate-649 and KEYNOTE-859 reported subgroup outcomes in Asian patients. The selection of CPS as an indicator of PD-L1 expression in tumor cells and immune cells was commonly observed in most of the included studies, whereas Rationale-305, using TAP, was used for assessment. The characteristics and informants of the four trials and the CheckMate-649 Chinese subgroup data are summarized in Supplementary Table S3.

### 3.3. Risk-of-Bias Assessment

The included studies underwent a comprehensive risk assessment, which revealed an overall low level of risk (Supplementary Figure S1).

### 3.4. Assessing the Efficacy of Reconstruction and Matching

Before conducting the analysis, the reconstruction quality was evaluated. For the PD-L1-positive population, we observed a high level of consistency between the reconstructed KM plot and the original image in terms of appearance, HRs, 95% confidence intervals (95% CIs), and the number of individuals at risk (Supplementary Table S4). These data provided strong support for us to conduct further survival analyses by reconstructing KM images. The aforementioned approach could be employed to generate OS and PFS images for a CheckMate-649 PD-L1 CPS of <1 and a CPS of <5, as well as for a CPS of 1–4 in CheckMate-649 and KEYNOTE-859. This methodology is also applicable to other clinical studies (Supplementary Table S5).

The follow-up time between matched pairs on the Bland–Altman plot and the empirical cumulative distribution plot exhibited negligible disparity (Supplementary Table S6). The differences between the reconstructed unmatched plots and the original unreported plots could be disregarded for each implementation of KMSubtraction, even after conducting 5000 iterations of the Monte Carlo simulations (Supplementary Table S7). The mean |ln (HR)| also demonstrated consistent outcomes (Supplementary Table S8).

### 3.5. A Summary of All Results of the HRs and Corresponding 95% CIs from Original Trials or KMSubtraction

**Figure 2:**
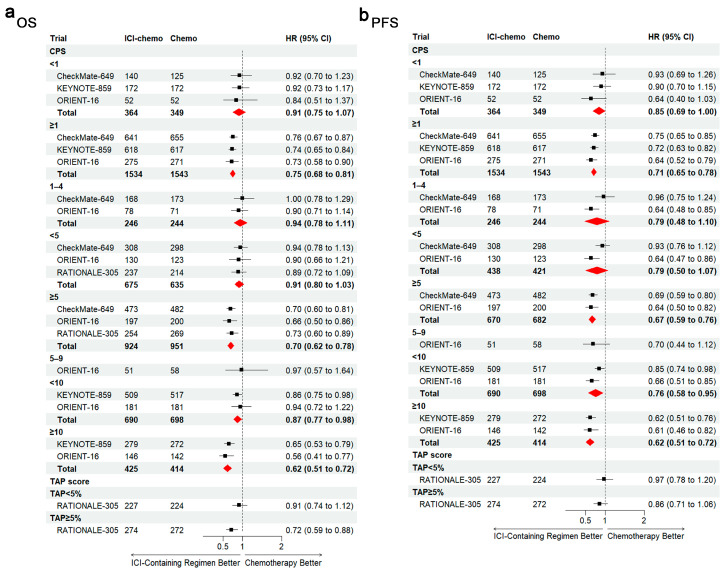
Hazard ratios (HRs) and corresponding 95% confidence intervals (CIs) for each PD-L1 CPS subgroup in terms of overall survival and progression-free survival. Hazard ratios (HRs) and corresponding 95% confidence intervals (CIs) for each PD-L1 CPS subgroup in terms of overall survival and progression-free survival: (**a**) overall and (**b**) progression free. The red diamond represents the result of the pooled analysis.

We calculated hazard ratios (HRs) and corresponding 95% CIs for each PD-L1 CPS subgroup (<1, ≥1, 1–4, <5, ≥5, 5–9, <10, and ≥10) for OS (Figure 2a) and PFS (Figure 2b) from the original trials that reported HRs or a KMSubtraction that did not provide subgroup analyses. The results indicate that patients with a PD-L1 CPS of ≥10 showed a significantly reduced risk of disease progression and death (OS: pooled HR, 0.62; 95% CI, 0.51–0.72; PFS: pooled HR, 0.62; 95% CI, 0.51–0.72). Patients with a PD-L1 CPS of 5–9 also demonstrated notable benefits in delaying disease progression with the addition of ICI immunotherapy, while HRs for OS did not favor ICI immunotherapy plus chemotherapy over chemotherapy alone (OS: pooled HR, 0.97; 95% CI, 0.57–1.64; PFS: pooled HR, 0.70; 95% CI, 0.44–1.12). However, patients with a low PD-L1 expression (OS: pooled HR, 0.94; 95% CI, 0.78–1.11; PFS: pooled HR, 0.79; 95% CI, 0.48–1.10) or a negative expression (OS: pooled HR, 0.91; 95% CI, 0.75–1.07; PFS: pooled HR, 0.85; 95% CI, 0.69–1.00) suggested less pronounced benefits. A pooled HR analysis highlighted that first-line ICI immunotherapy does not reduce the risk of disease progression and death in non-HER2-positive/HER2-negative advanced or metastatic GC or GEJC with a negative expression of PD-L1 or a low expression of it.

### 3.6. Overall Survival

**Figure 3:**
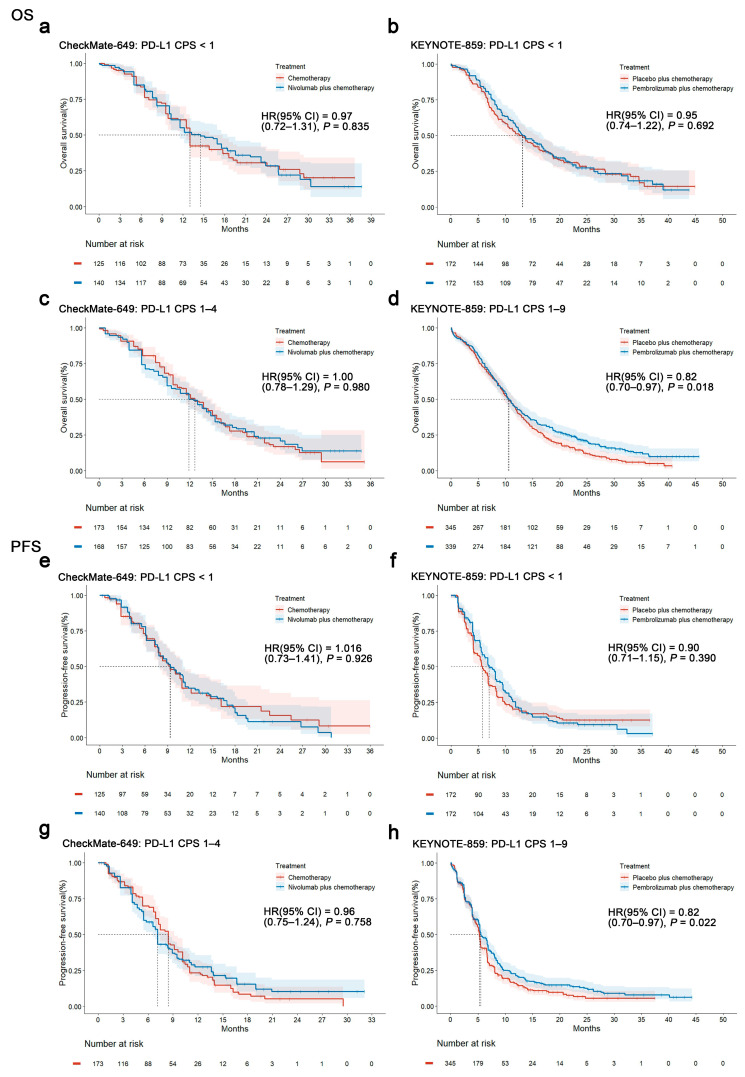
Kaplan–Meier plots for overall survival and progression-free survival in low programmed death ligand 1 (PD-L1) subgroups derived with KMSubtraction. Overall survival–Kaplan–Meier plots derived by KMSubtraction: (**a**) CheckMate-649 PD-L1 CPS of <1. (**b**) KEYNOTE-859 PD-L1 CPS of <1. (**c**) CheckMate-649 PD-L1 CPS of 1–4. (**d**) KEYNOTE-859 PD-L1 CPS of 1–9. Progression-free survival–Kaplan–Meier plots derived using KMSubtraction: (**e**) CheckMate-649 PD-L1 CPS of <1. (**f**) KEYNOTE-859 PD-L1 CPS of <1. (**g**) CheckMate-649 PD-L1 CPS of 1–4. (**h**) KEYNOTE-859 PD-L1 CPS of 1–9. CPS, combined positive score; HR, hazard ratio; PD-L1, programmed death ligand 1.

We conducted an OS analysis based on the original survival curve data of subgroups with a negative or low PD-L1 expression in the different studies. In the CheckMate-649 cohort, nivolumab plus chemotherapy did not show a significant difference in OS compared to chemotherapy alone in patients with a negative PD-L1 expression (HR, 0.97; 95% CI, 0.72–1.31; *p* = 0.835, Figure 3a). Similarly, in the KEYNOTE-859 PD-L1-negative subgroup with pembrolizumab plus chemotherapy did not significantly improve OS compared to chemotherapy alone (HR, 0.95; 95% CI, 0.74–1.22; *p* = 0.692, Figure 3b). In the CheckMate-649 subgroup with a PD-L1 CPS of 1–4, no significant OS difference was observed with nivolumab–chemotherapy compared to chemotherapy alone (HR, 1.00; 95% CI, 0.78–1.29; *p* =0.980; Figure 3c). However, in the KEYNOTE-859 subgroup with a PD-L1 CPS of 1–9, pembrolizumab significantly extended OS compared with a placebo plus chemotherapy (HR, 0.82; 95% CI, 0.70–0.97; *p* = 0.018; Figure 3d). The RATIONALE-305 study showed no significant difference in OS for the subgroup with a TAP of <5% for tislelizumab plus chemotherapy (HR, 0.97; 95% CI, 0.78–1.20; *p =* 0.780; Supplementary Figure S2A) compared with a placebo plus chemotherapy. The ORIENT-16 study showed no significant OS difference for the subgroup with a CPS of <5 for sintilimab plus chemotherapy (HR, 0.90; 95% CI, 0.67–1.23; *p* = 0.540; Supplementary Figure S3A) compared to a placebo plus chemotherapy. In general, no difference in OS was observed between the ICI-plus-chemotherapy and chemotherapy-only groups across all studies in the PD-L1-negative or low-PD-L1-expression subgroups, determined by CPS or TAP. The KM curves for all patients with a CPS of 1–4 were not clinically helpful. The KEYNOTE-859 study, which only provided PD-L1 CPS scores of 1–9 (including segments with a low and moderate PD-L1 expression), showed an OS prolongation trend.

### 3.7. Progression-Free Survival

We further conducted a PFS analysis based on the original survival curve data of subgroups with a negative or low PD-L1 expression in the different studies. Nivolumab plus chemotherapy did not show a significant PFS difference compared to chemotherapy alone for patients with a PD-L1 CPS of <1 in CheckMate-649 (HR, 1.016; 95% CI, 0.73–1.41; *p* = 0.926; Figure 3e). Similarly, in the KEYNOTE-859 subgroup with a PD-L1 CPS of <1, pembrolizumab plus chemotherapy did not significantly improve PFS compared to chemotherapy alone (HR, 0.90; 95% CI, 0.71–1.15; *p* = 0.390; Figure 3f). In the CheckMate-649 subgroup with a PD-L1 CPS of 1–4, no significant PFS difference was observed between nivolumab plus chemotherapy and chemotherapy alone (HR, 0.96; 95% CI, 0.75–1.24; *p* = 0.758; Figure 3g). Furthermore, in the KEYNOTE-859 subgroup with a PD-L1 CPS of 1–9, pembrolizumab significantly extended PFS (HR, 0.82; 95% CI, 0.70–0.97; *p* = 0.022; Figure 3h). The RATIONALE-305 study showed no significant difference in PFS for the subgroup with a TAP of <5% for tislelizumab plus chemotherapy (HR, 0.86; 95% CI, 0.71–1.06; *p* = 0.145; Supplementary Figure S2B) compared to a placebo plus chemotherapy. However, the ORIENT-16 study showed a significant PFS difference in the subgroup with a CPS of <5 for sintilimab plus chemotherapy (HR, 0.65; 95% CI, 0.49–0.88; *p* = 0.005; Supplementary Figure S3B) compared to a placebo plus chemotherapy. In summary, no difference in PFS was observed between the ICI-plus-chemotherapy and chemotherapy-only groups in derived subgroups with a negative or low PD-L1 expression, determined by CPS or TAP in CheckMate-649, KEYNOTE-859 (also including segments with a mid-PD-L1 expression: CPS 5–9), and RATIONALE-305. Except for the ORIENT-16 study, which only provided 0–5 scores of PD-L1 (including negative and low expressions), a PFS prolongation trend was observed.

### 3.8. One-Stage IPD-Pooled Analysis Based on PD-L1 CPS Scoring System

**Figure 4:**
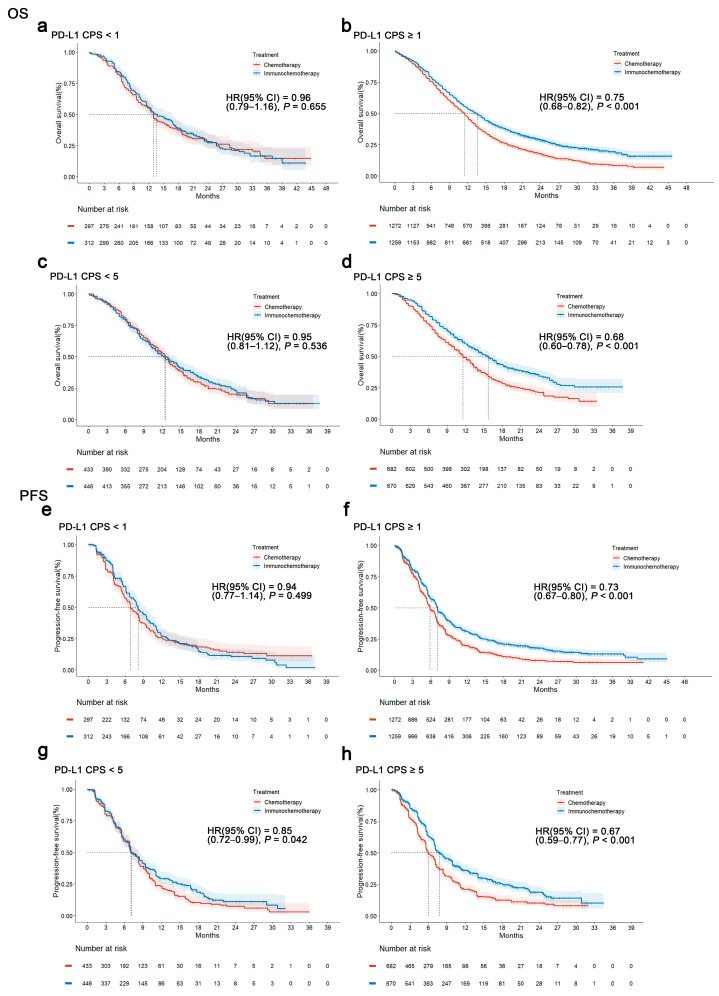
One-stage IPD-pooled analysis of first-line studies (CheckMate-649, KEYNOTE-859, and ORIENT-16). (**a**) Overall survival in first-line studies with a CPS of <1. (**b**) Overall survival in first-line studies with a CPS of ≥1. (**c**) Overall survival in first-line studies with a CPS of <5. (**d**) Overall survival in first-line studies with a CPS of ≥5. (**e**) Progression-free survival in first-line studies with a CPS of <1. (**f**) Progression-free survival in first-line studies with a CPS of ≥1. (**g**) Progression-free survival in first-line studies with a CPS of <5. (**h**) Progression-free survival in first-line studies with a CPS of ≥5.

An IPD-pooled analysis of first-line trials assessing PD-L1 expression based on CPS (CheckMate-649, KEYNOTE-859, and ORIENT-16) revealed OS outcomes comparing immunochemotherapy versus chemotherapy alone across various patient populations. In the intention-to-treat (ITT) population, immunochemotherapy demonstrated a significant improvement in OS compared to chemotherapy alone (HR, 0.78; 95% CI, 0.72–0.84; *p* < 0.001; Supplementary Figure S4A). Among patients with a PD-L1 CPS of <1, there was no significant difference in OS between immunochemotherapy and chemotherapy alone (HR, 0.96; 95% CI, 0.79–1.16; *p* = 0.655; Figure 4a). However, in patients with a PD-L1 CPS of ≥1, immunochemotherapy significantly improved OS compared to chemotherapy alone (HR, 0.75; 95% CI, 0.68–0.82; *p* < 0.001; Figure 4b). For patients with a PD-L1 CPS of <5, there was no significant difference in OS between immunochemotherapy and chemotherapy alone (HR, 0.95; 95% CI, 0.81–1.12; *p* = 0.536; Figure 4c). Among patients with a PD-L1 CPS of ≥5, immunochemotherapy significantly extended OS compared with chemotherapy alone (HR, 0.68; 95% CI, 0.60–0.78; *p* < 0.001; Figure 4d). The IPD-pooled analysis results confirm that non-HER2-positive/HER2-negative advanced or metastatic GC or GEJC with negative and low PD-L1 expressions had no significant OS benefits from first-line ICI immunotherapy.

In the ITT population, immunochemotherapy showed a significant improvement in PFS compared with chemotherapy alone (HR, 0.74; 95% CI, 0.69–0.80; *p* < 0.001; Supplementary Figure S4B). In patients with a PD-L1 CPS of <1, there was no significant difference in PFS between immunochemotherapy and chemotherapy alone (HR, 0.94; 95% CI, 0.77–1.14; *p* = 0.499; Figure 4e). However, in patients with a PD-L1 CPS of ≥1, immunochemotherapy significantly improved PFS compared to chemotherapy alone (HR, 0.73; 95% CI, 0.67–0.80; *p* < 0.001; Figure 4f). For patients with a PD-L1 CPS of <5, immunochemotherapy also showed a significant benefit in PFS compared to chemotherapy alone, albeit with a more modest effect (HR, 0.85; 95% CI, 0.72–0.99; *p* = 0.042; Figure 4g). Similarly, among patients with a PD-L1 CPS of ≥5, immunochemotherapy significantly improved PFS compared to chemotherapy alone (HR, 0.67; 95% CI, 0.59–0.77; *p* < 0.001; Figure 4h). The IPD-pooled analysis results confirm that non-HER2-positive/HER2-negative advanced or metastatic GC or GEJC with a negative PD-L1 expression had no significant PFS benefit from first-line immunotherapy. However, a low PD-L1 expression may have weak statistical significance for PFS benefits from first-line ICI immunotherapy.

### 3.9. One-Stage IPD-Pooled Analysis Based on Chemo Programs

The pooled IPD analysis of all first-line trials (CheckMate-649, KEYNOTE-859, ORIENT-16, and RATIONALE-305) showed a significant OS difference for immunochemotherapy (HR, 0.79; 95% CI, 0.74–0.84; *p* < 0.001; Supplementary Figure S5A) compared to chemotherapy alone. There was also a significant PFS difference for immunochemotherapy (HR, 0.75; 95% CI, 0.70–0.80; *p* < 0.001; Supplementary Figure S5B) compared to chemotherapy alone.

**Figure 5:**
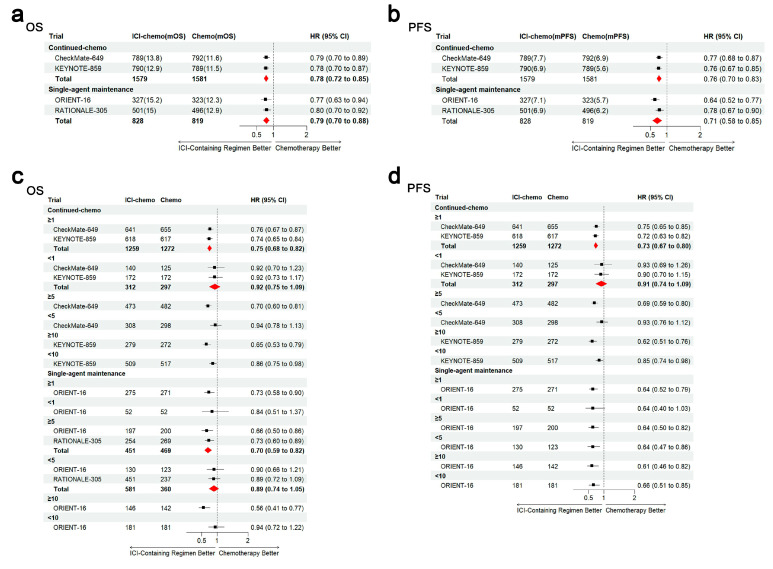
Forest plot results of overall survival and progression-free survival between ICI immunotherapy combined with different chemotherapy models. CheckMate-649 and KEYNOTE-859: continuous combination of ICI immunotherapy and two-drug chemotherapy. ORIENT-16 and RATIONALE-305: ICI immunotherapy and chemotherapy with two stages: induction of two-drug chemotherapy and single-drug maintenance. (**a**) Overall survival; (**b**) progression-free survival; (**c**) subgroup analysis of overall survival; (**d**) subgroup analysis of progression-free survival. The red diamond represents the result of the pooled analysis.

Next, we will carefully analyze the impact of the chemotherapy programs of these four studies on our analysis results, in which the main difference lies in the chemotherapy framework regimen. The CheckMate-649 and KEYNOTE-859 study design included the following (continued combined chemotherapy): a continuous combination of ICI immunotherapy and doublet chemotherapy. The ORIENT-16 and RATIONALE-305 study design included (single-agent maintenance) continuous ICI immunotherapy, while the chemotherapy framework was divided into two stages: the induction of doublet chemotherapy and follow-up single-drug maintenance. Furthermore, we calculated the HRs and 95% CIs. The results show no significant difference in OS (Figure 5a) and PFS (Figure 5b) between the continued combined chemo model (OS: pooled HR, 0.78; 95% CI, 0.72–0.85; PFS: pooled HR, 0.76; 95% CI, 0.70–0.83) and the single-agent maintenance model (OS: pooled HR, 0.79; 95% CI, 0.70–0.88; PFS: pooled HR, 0.71; 95% CI, 0.58–0.85). Other subgroup analyses (Figure 5c,d) suggested a comparable magnitude of benefits between immunotherapy combined with a continuous chemotherapy regimen and immunotherapy combined with induction chemotherapy followed by maintenance therapy.

We further compared the differences in adverse events between the two types of chemotherapy models: CheckMate-649/KEYNOTE-859 (continued combined chemo) and ORIENT-16/RATIONALE-305 (single-agent maintenance). The occurrence rates of adverse events for all grades (97% vs. 97%) were similar. The occurrence rate of grade 3–5 treatment-related adverse events was higher for CheckMate-649/KEYNOTE-859 (continued combined chemotherapy) than for ORIENT-16/RATIONALE-305 (single-agent maintenance) (59% vs. 56%). The occurrence rate of leading to discontinuation and death, CheckMate-649/KEYNOTE-859 (continued combined chemotherapy), was more common (31% vs. 16% and 5% vs. 1%) than ORIENT-16/RATIONALE-305 (single-agent maintenance) (Supplementary Table S9). These results imply that the maintenance-treatment model of monotherapy chemotherapy might have better tolerance than the continuous model of doublet chemotherapy.

## 4. Discussion

Gastric and gastroesophageal cancer are frequently diagnosed at advanced stages, with limited treatment options and a poor prognosis [22]. The emergence of immune checkpoint inhibitors has brought new hope, but the optimal use of these therapies remains challenging due to the complex interplay between the tumor and immune system. The integration of chemotherapy (or targeted therapy) with immune checkpoint inhibitors is rapidly emerging as a promising strategy against several tumor types [23]. In an effort to further optimize treatment regimens for patients, PD-L1 expression has been extensively investigated as a predictive marker for the response and efficacy of programmed cell death protein-1 inhibitors. However, due to the dynamic interplay between these antibodies and the immune microenvironment, as well as the variability in the immune milieu across different tumor types, the available evidence surrounding the predictiveness of PD-L1 expression is fraught with contradiction and ambiguity [24,25,26,27]. Moreover, gastroesophageal cancer is notoriously known for its heterogeneity, exhibiting both intra- and intertumoral heterogeneity, which further hinders advancements in precision oncology for this disease [28,29].

Several phase 3 clinical trials of first-line immune checkpoint inhibitors (ICIs) in non- HER2-positive or HER2-negative advanced or metastatic GC or GEJC have shown a superior objective response rate (ORR), PFS, and OS [3,4,5,6]. However, evidence suggests that patients with a higher PD-L1 expression achieve better therapeutic effects and survival benefits [14,30,31]. We aimed to analyze the currently approved ICIs, including CheckMate-649, KEYNOTE-859, ORIENT-16, and RATIONALE-305 deeply to determine which populations with PD-L1 expression truly benefit. We also focus on variation among the included trials: tumor heterogeneity, patient population, methodology enrolled populations, study designs, and PD-L1 assays.

The OS analysis via KMSubtraction showed no significant improvement in the PD-L1-negative or -low subgroups in CheckMate-649 (CPS < 1) and KEYNOTE-859 (CPS < 1), as well as CheckMate-649 (CPS 1–4), ORIENT-16 (CPS 0–4), and RATIONALE-305 (TAP 0–4%). However, KEYNOTE-859 showed a statistically significant OS benefit, with an 18% reduction in the risk of death among patients with low to moderate PD-L1 expression (CPS 1–9, HR, 0.82; 95% CI, 0.70–0.97; *p* = 0.018) based on the original data. This may be due to differences in the following factors, especially in KEYNOTE-859: (1) the OS analysis covered most patients with a moderate expression of PD-L1; (2) the PD-L1-detection method and interpretation of the positive population are different from other studies; (3) all included patients were HER2-negative; and (4) there were differences in the regimens with platinum drugs combined with chemotherapy.

The PFS analysis via KMSubtraction showed no significant improvement in the PD-L1-negative or -low subgroups in CheckMate-649 (CPS < 1), KEYNOTE-859 (CPS < 1), CheckMate-649 (CPS 1–4), and RATIONALE-305 (TAP 0–4%). Conversely, ORIENT-16 showed a significant PFS improvement, with a 35% reduction in the risk of progression in the PD-L1-negative or -low subgroup (CPS 0–4, HR, 0.65; 95% CI, 0.49–0.88; *p* = 0.005). The inconsistency between the results of the ORIENT-16 PFS analysis and other studies may be due to the following factors, especially in ORIENT-16: (1) ORIENT-16 was completed only in China, resulting in relative regional limitations; (2) the proportion of people with a PD-L1 CPS of ≥10 exceeded 44% (Supplementary Table S10); (3) the proportion of patients with primary GC was higher than that in other studies; (4) the proportion of locally advanced GC or GEJC patients was higher than that in other studies, while the number of metastatic GC or GEJC patients was lower than other studies; and (5) the proportion of patients with liver and peritoneal metastases was lower than that in other studies [5].

The one-stage IPD-pooled analysis, based on the PD-L1 CPS scoring system, confirmed that non-HER2-positive/HER2-negative advanced or metastatic GC or GEJC with a negative PD-L1 expression (CPS < 1) and a low expression (CPS < 5) had no significant OS and PFS benefits from first-line immunotherapy. Patients with a PD-L1 CPS of ≥5 had a significantly extended OS, with a 32% reduction in the risk of death (HR, 0.68; 95% CI, 0.60–0.78; *p* < 0.001), and an extended PFS, with a 33% reduction in the risk of progression (HR, 0.67; 95% CI, 0.59–0.77; *p* < 0.001), compared to chemotherapy alone. Furthermore, a pooled-HR analysis highlighted that first-line ICI immunotherapy does not reduce the risk of disease progression and death in patients with a negative or low PD-L1 expression. However, the pooled-HR analysis indicated that patients with CPS scores of 5–9 may not have any survival benefits from first-line immunotherapy. While the NCCN guidelines suggest benefits for the PD-L1 5–9 group, our analysis is limited by the small sample size from the ORIENT-16 study. We recognize that other studies have shown benefits for a PD-L1 of >5, but the lack of data for the 5–9 subgroup in those studies limits our ability to draw definitive conclusions. We will elaborate on this point in our discussion. This suggests that future clinical research should refine the hierarchical design and data analysis for PD-L1 expression. Based on different research design schemes, CheckMate-649, KEYNOTE-859, ORIENT-16, and RATIONALE-305 were included in the pooled analysis. These results suggest that immunotherapy combined with the induction of doublet chemotherapy followed by maintenance single-agent chemotherapy offered equal survival benefits but better tolerance than continuous doublet chemotherapy.

These findings may potentially enhance the nuanced utilization of the PD-L1 expression score in clinical practice. Firstly, by employing PD-L1 as a negative predictive biomarker with a dichotomous score (positive and negative), clinicians may be able to avoid the unnecessary toxicity and economic burden associated with immunotherapy in GC or GEJC patients with a negative or low PD-L1 expression [32,33]. Secondly, when considering PD-L1 as a continuous variable within the PD-L1-positive population, clinicians may be better equipped to inform patients about the potential magnitude of benefit they may derive from the addition of immune checkpoint inhibitors (ICIs) [34].

### Limitations

The limitations of the KMSubtraction method mainly include the following: larger error ranges when the dataset is small or when there is a higher proportion of missing data in the unreported subgroup; applicability only to binary variables, which restricts the summarization of unreported-subgroup data across different cutoff values; and the inability to extract individual patient-level covariate data, precluding further causal inference analyses. Caution is needed when interpreting results from KMSubtraction, and combining it with other methods or data sources can enhance the reliability of the analysis [12]. It is important to acknowledge the limitations of this study when interpreting these findings and their implications for clinical practice. First, the sample size was limited to the subgroups with a negative or low PD-L1 expression that were included in the various studies analyzed. Second, even if the population of unreported subgroups can be reconstructed through individual patient data, there may still be data loss due to the absence of original data from individuals belonging to subgroups with a negative or low PD-L1 expression. Third, despite conducting an error analysis on the derived HRs, it is undeniable that some minor differences exist between the original HRs and the derived HRs. Finally, the survival benefits observed in the KEYNOTE-859 subgroup population with a PD-L1 CPS of 1–9 do not provide sufficient evidence to determine whether a higher proportion of a positive CPS (CPS 5–9) exists in the enrolled patients.

## 5. Conclusions

In conclusion, the overall evidence in our study demonstrates that first-line immunotherapy for non-HER2-positive/HER2-negative advanced or metastatic GC or GEJC may not provide additional OS or PFS benefits in the negative-PD-L1-expressing (CPS < 1) or PD-L1-low subgroups (CPS 1–4/TAP < 5%) as compared with chemotherapy alone. Patients with a high PD-L1 CPS score (CPS ≥ 10) have significant benefits in terms of OS and PFS. However, patients with a CPS score designating a moderate expression of PD-L1 (CPS 5–9) indicate unclear OS and PFS benefits due to a lack of sufficient evidence. It is very meaningful that first-line immunotherapy combined with the induction of doublet chemotherapy followed by single-agent chemotherapy may be more suitable for practical clinical applications than immunotherapy combined with a continuous induction of doublet chemotherapy. The findings of this study might help clinicians make optimal decisions and provide a scientific basis to design rational clinical research on first-line immunotherapy for advanced or metastatic GC or GEJC in the future.

## Data Availability

Data are contained within the article.

## Supplementary Materials

The following supporting information can be downloaded at: https://www.mdpi.com/article/10.3390/cancers17040657/s1, Supplementary Figure S1: Risk-of-bias assessment; Supplementary Figure S2: KMSubtraction of OS and PFS for low-PD-L1-expression subgroups in RATIONALE-305 (using TAP scores to record the level of PD-L1 expression); Supplementary Figure S3: KMSubtraction of OS and PFS for low-PD-L1-expression subgroups in trials: ORIENT-16 (using CPS scores to record the level of PD-L1 expression); Supplementary Figure S4: One-stage pooled analysis of first-line studies (CheckMate-649, KEYNOTE-859, and ORIENT-16); Supplementary Figure S5: one-stage pooled analysis of first-line studies (CheckMate-649, KEYNOTE-859, ORIENT-16, and RATIONALE-305); Supplementary Table S1: Search information; Supplementary Table S2: Search information and KMSubtraction implementations; Supplementary Table S3: Baseline characteristics and clinical information of the trials; Supplementary Table S4: Compared with original curves; Supplementary Table S5: Example of KMSubtraction outcomes compared with reported HRs for low PD-L1 expression subgroups; Supplementary Table S6: Evaluation of KMSubtraction bipartite matching; Supplementary Table S7: Simulated limits of errors of KMSubtraction per implementation; Supplementary Table S8: Convergence plots and histograms of simulations; Supplementary Table S9: Treatment-related adverse events; Supplementary Table S10: Subgroup analysis by PD-L1 CPS subpopulations.

## Abbreviations

ICI	Immune checkpoint inhibitor
GC	Gastric cancer
GEJC	Gastroesophageal junction cancer
HER2	Human epithelial growth factor receptor 2
PD-1	Programmed death 1
PD-L1	Programmed cell death ligand 1
CPS	Combined positive score
TAP	Tumor area positivity
PFS	Progression-free survival
OS	Overall survival
KM	Kaplan–Meier
FDA	The Food and Drug Administration
NMPA	The National Medical Products Administration
PRISMA	Preferred Reporting Items for Systematic Reviews and Meta-Analyses
IPD	Individual patient data
IRB	Institutional review board
RCTs	Randomized controlled trials
RoB 2	Cochrane Collaboration’s risk of bias
PH	Proportional hazards
HR	Hazard ratio
CIs	Confidence intervals
