# Supplementary material for: Deeper Analysis to Identify the True Benefit of ICIs Immunotherapy in First-Line Treatment for Non-HER2-Positive/HER2-Negative Advanced or Metastatic Advanced or Metastatic Gastric Cancer (GC) or Gastroesophageal Junction Cancer (GEJC)
Source: Cancers (Basel). 2025 Feb 15;17(4):657. doi: 10.3390/cancers17040657 (PMC11852535; doi:10.3390/cancers17040657)
Supplement: Supplementary file 1 [file cancers-17-00657-s001.zip › cancers-3446993-supplementary.pdf]

Data Supplementary

Supplementary Figure S1: Risk-of-Bias assessment
Supplementary Figure S2: KMSubtraction of OS and PFS for low PD-L1 expression subgroups in RATIONALE-305 (Using TAP scores to record the level of PD-L1 expression)
Supplementary Figure S3: KMSubtraction of OS and PFS for low PD-L1 expression subgroups in trials: ORIENT-16 (Using CPS scores to record the level of PD-L1 expression)
Supplementary Figure S4: One-Stage Pooled Analysis of First-line Studies. (CheckMate-649, KEYNOTE-859, ORIENT-16)
Supplementary Figure S5: One-Stage Pooled Analysis of First-line Studies (CheckMate-649, KEYNOTE-859, ORIENT-16 and RATIONALE-305)
Supplementary Table S1: Search information
Supplementary Table S2: Search information and KMSubtraction implementations
Supplementary Table S3: Baseline characteristics and clinical information of the trials
Supplementary Table S4: Compared with original curves
Supplementary Table S5: Example of KMSubtraction outcomes compared with reported HRs for low PD-L1 expression subgroups
Supplementary Table S6: Evaluation of KMSubtraction bipartite matching
Supplementary Table S7: Simulated limits of error of KMSubtraction per implementation
Supplementary Table S8: Convergence Plots and Histograms of Simulations
Supplementary Table S9: Treatment-related adverse events
Supplementary Table S10: Subgroup analysis by PD-L1 CPS subpopulations

Supplementary Figure S1: Risk-of-Bias assessment

Supplementary Figure S1

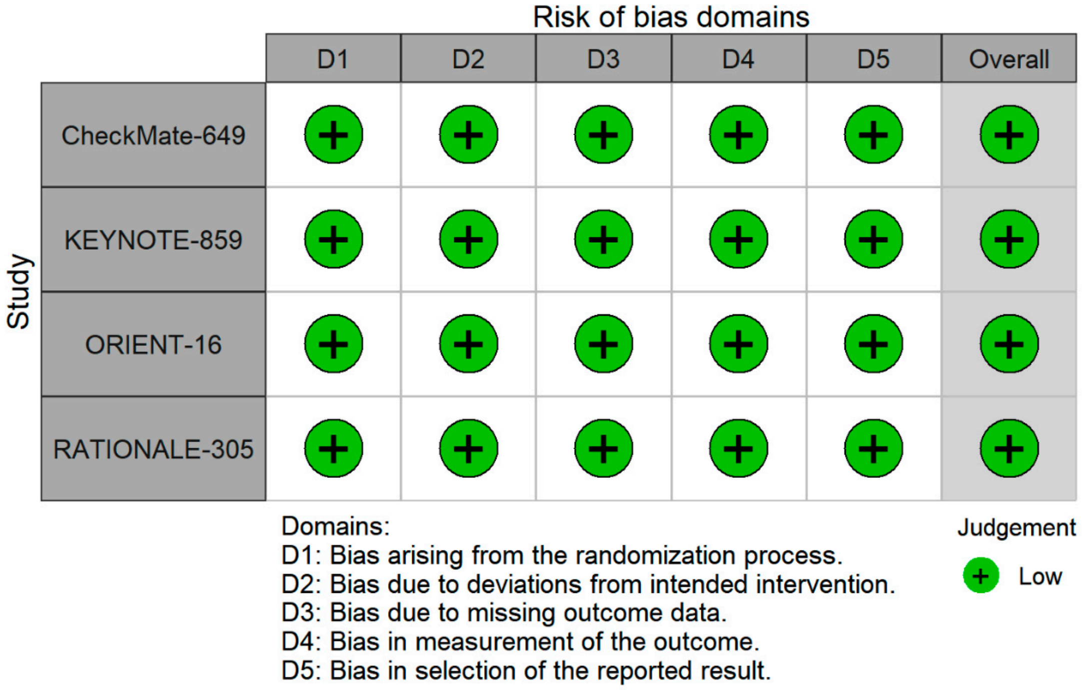

Abbreviations: D, domain.

Supplementary Figure S2: KMSubtraction of OS and PFS for low PD-L1 expression subgroups in RATIONALE-305 (Using TAP scores to record the level of PD-L1 expression)

Supplementary Figure S2

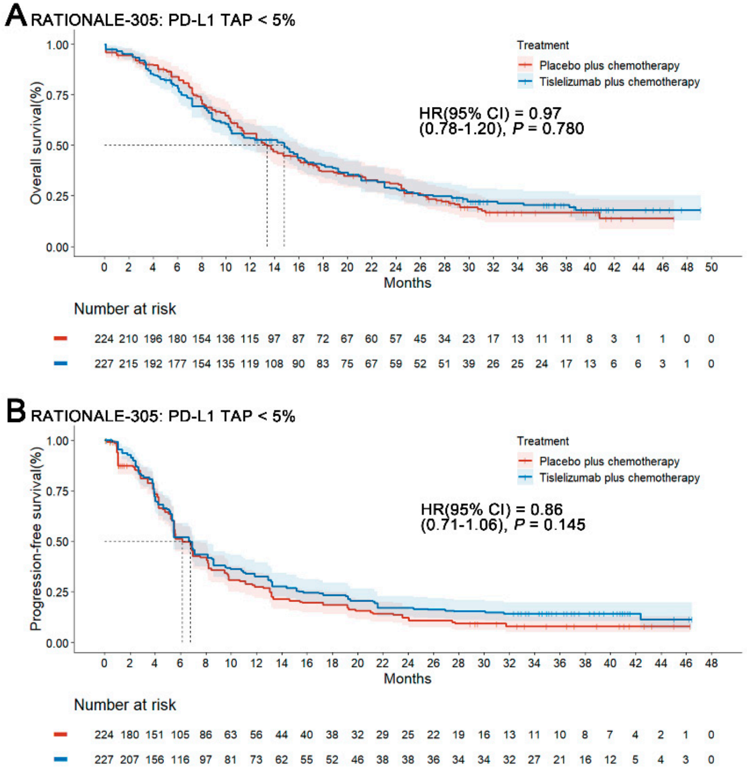

Supplementary Figure S2: Results of KMSubtraction from RATIONALE-305----OS and PFS: (A) OS PD-L1 TAP <5%; (B) PFS PD-L1 TAP <5%; TAP, tumor area positivity; HR, hazard ratio; CI, confidence interval; OS, overall survival; PFS, progression free survival; PD-L1, programmed death-ligand 1

Supplementary Figure S3: KMSubtraction of OS and PFS for low PD-L1 expression subgroups in trials: ORIENT-16 (Using CPS scores to record the level of PD-L1 expression)

Supplementary Figure S3

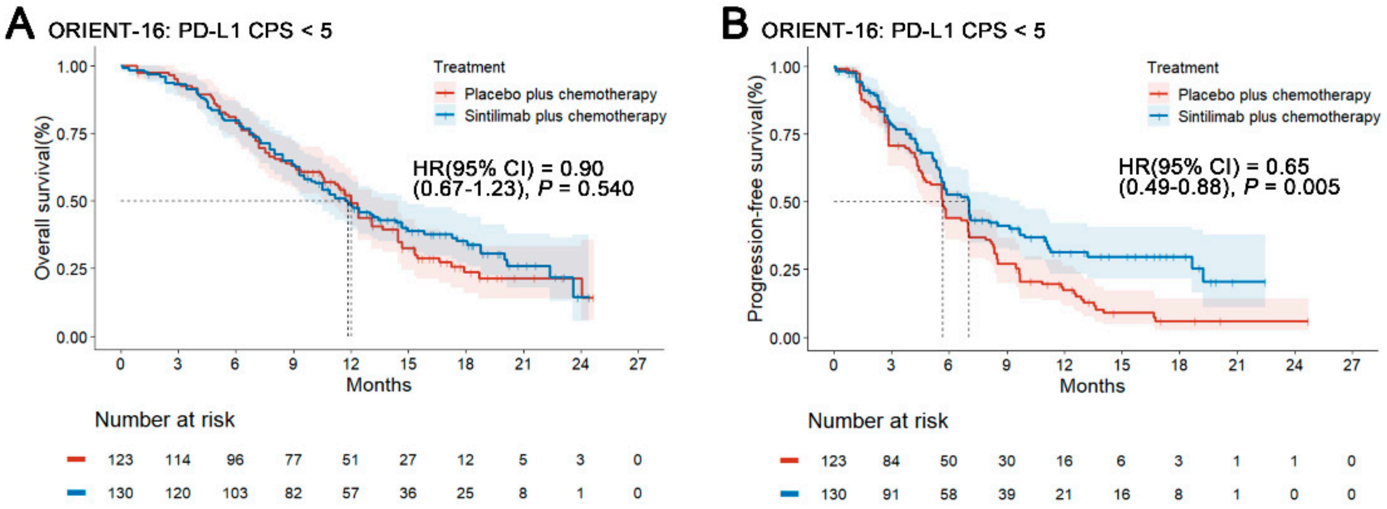

Supplementary Figure S3: Results of KMSubtraction from ORIENT-16—OS and PFS: (A) OS ORIENT-16 PD-L1 CPS<5; (B) PFS ORIENT-16 PD-L1 CPS<5 CPS combined positive score; HR, hazard ratio; CI, confidence interval; OS, overall survival; PFS, progression free survival; PD-L1, programmed death-ligand

Supplementary Figure S4: One-Stage Pooled Analysis of First-line Studies. (CheckMate-649, KEYNOTE-859, ORIENT-16)

Supplementary Figure S4

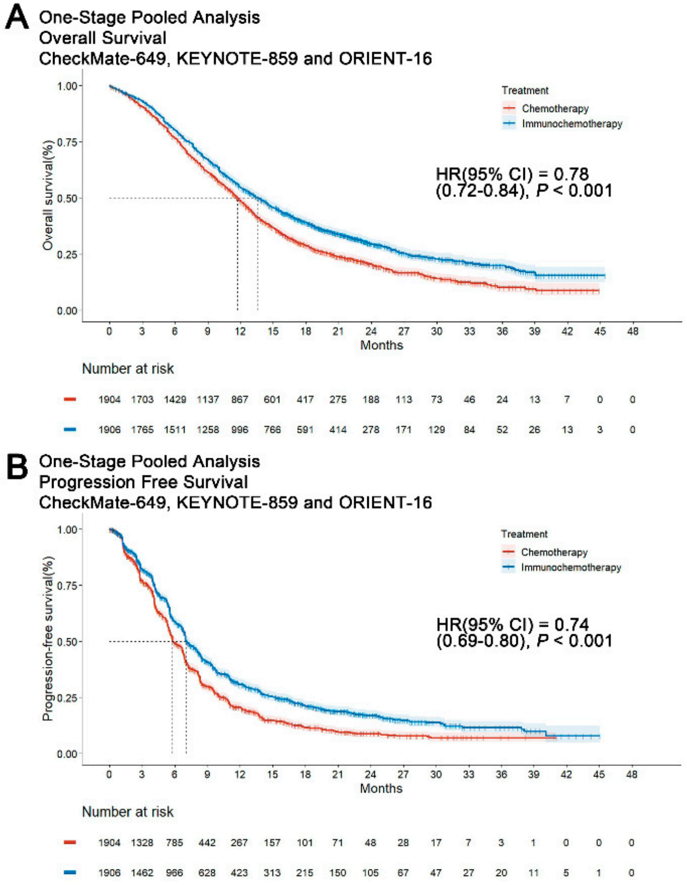

Supplementary Figure S4: (A) One stage Pooled-analyses of Overall Survival in first-line studies. (B) One stage Pooled-analyses of Progression Free Survival in first-line studies.

Supplementary Figure S5: One-Stage Pooled Analysis of First-line Studies (CheckMate-649, KEYNOTE-859, ORIENT-16 and RATIONALE-305)

Supplementary Figure S5

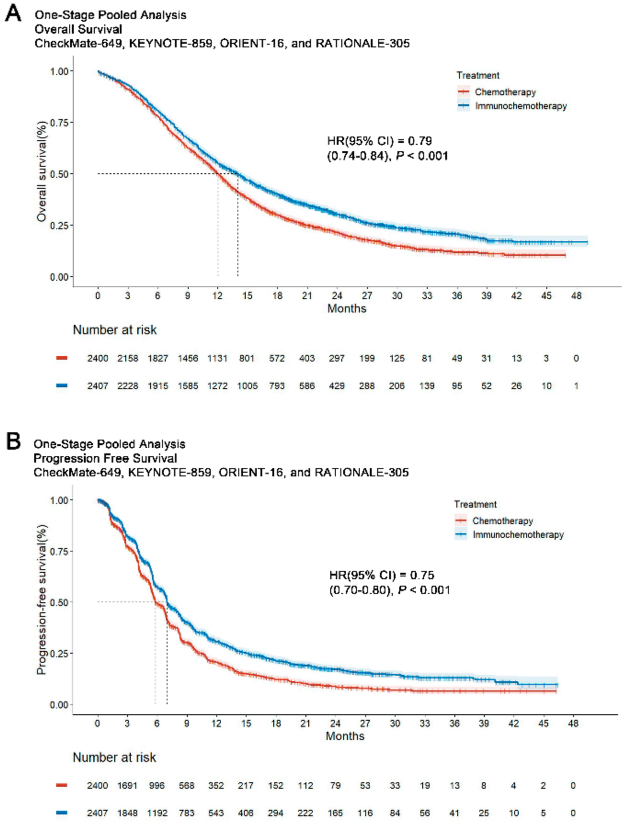

Supplementary Figure S5: One-Stage Pooled Analysis of First-line Studies (CheckMate-649, KEYNOTE-859, ORIENT-16 and RATIONALE-305). (A) One stage Pooled-analyses of Overall Survival in first-line studies. (B) One stage Pooled-analyses of Progression Free Survival in first-line studies.

Supplementary Table S1: Search information

Date of search	18 May 2024
**Databases**	**PubMed, Embase, Web of Science and Cochrane**
**Search example of Pubmed**	((((("Neoplasms"[Mesh]) OR (Neoplasms[Title/Abstract])) OR ((((((((((((Tumor[Title/Abstract]) OR (Neoplasm[Title/Abstract])) OR (Tumors[Title/Abstract])) OR (Neoplasias[Title/Abstract])) OR (Cancer[Title/Abstract])) OR (Cancers[Title/Abstract])) OR (Malignant Neoplasm[Title/Abstract])) OR (Malignancy[Title/Abstract])) OR (Malignancies[Title/Abstract])) OR (Malignant Neoplasms[Title/Abstract])) OR (Neoplasm, Malignant[Title/Abstract])) OR (Neoplasms, Malignant[Title/Abstract]))) AND ((((((("Stomach"[Mesh]) OR (Stomach[Title/Abstract])) OR (Stomachs[Title/Abstract])) OR ("Esophagogastric Junction"[Mesh])) OR (Esophagogastric Junction[Title/Abstract])) OR (((((Junction, Esophagogastric[Title/Abstract]) OR (Gastroesophageal Junction[Title/Abstract])) OR (Gastroesophageal Junctions[Title/Abstract])) OR (Junction, Gastroesophageal[Title/Abstract])) OR (Junctions, Gastroesophageal[Title/Abstract]))) OR (Gastric))) AND (((((((((((((((((((((((("Immune Checkpoint Inhibitors"[Mesh]) OR (Immune Checkpoint Inhibitors[Title/Abstract])) OR (((((((((((((((((((((((((((((((((Checkpoint Inhibitors, Immune[Title/Abstract]) OR (Immune Checkpoint Inhibitor[Title/Abstract])) OR (Checkpoint Inhibitor, Immune[Title/Abstract])) OR (Immune Checkpoint Blockers[Title/Abstract])) OR (Checkpoint Blockers, Immune[Title/Abstract])) OR (Immune Checkpoint Blockade[Title/Abstract])) OR (Checkpoint Blockade, Immune[Title/Abstract])) OR (Immune Checkpoint Inhibition[Title/Abstract])) OR (Checkpoint Inhibition, Immune[Title/Abstract])) OR (PD-L1 Inhibitors[Title/Abstract])) OR (PD L1 Inhibitors[Title/Abstract])) OR (PD-L1 Inhibitor[Title/Abstract])) OR (PD L1 Inhibitor[Title/Abstract])) OR (Programmed Death-Ligand 1 Inhibitors[Title/Abstract])) OR (Programmed Death Ligand 1 Inhibitors[Title/Abstract])) OR (PD-1-PD-L1 Blockade[Title/Abstract])) OR (Blockade, PD-1-PD-L1[Title/Abstract])) OR (PD 1 PD L1 Blockade[Title/Abstract])) OR (CTLA-4 Inhibitors[Title/Abstract])) OR (CTLA 4 Inhibitors[Title/Abstract])) OR (CTLA-4 Inhibitor[Title/Abstract])) OR (CTLA 4 Inhibitor[Title/Abstract])) OR (Cytotoxic T-Lymphocyte-Associated Protein 4 Inhibitors[Title/Abstract])) OR (Cytotoxic T Lymphocyte Associated Protein 4 Inhibitors[Title/Abstract])) OR (Cytotoxic T-Lymphocyte-Associated Protein 4 Inhibitor[Title/Abstract])) OR (Cytotoxic T Lymphocyte Associated Protein 4 Inhibitor[Title/Abstract])) OR (PD-1 Inhibitors[Title/Abstract])) OR (PD 1 Inhibitors[Title/Abstract])) OR (PD-1 Inhibitor[Title/Abstract])) OR (Inhibitor, PD-1[Title/Abstract])) OR (PD 1 Inhibitor[Title/Abstract])) OR (Programmed Cell Death Protein 1 Inhibitor[Title/Abstract])) OR (Programmed Cell Death Protein 1 Inhibitors[Title/Abstract]))) OR ("Immunotherapy"[Mesh])) OR (Immunotherapy[Title/Abstract])) OR (Immunotherapies[Title/Abstract])) OR ("Nivolumab"[Mesh])) OR (Nivolumab[Title/Abstract])) OR ("sintilimab" [Supplementary Concept])) OR (sintilimab[Title/Abstract])) OR ("tislelizumab" [Supplementary Concept])) OR (tislelizumab[Title/Abstract])) OR ("toripalimab" [Supplementary Concept])) OR (toripalimab[Title/Abstract])) OR ("pembrolizumab" [Supplementary Concept])) OR (pembrolizumab[Title/Abstract])) OR ("camrelizumab" [Supplementary Concept])) OR (camrelizumab[Title/Abstract])) OR ("Ipilimumab"[Mesh])) OR (Ipilimumab[Title/Abstract])) OR ("avelumab" [Supplementary Concept])) OR (avelumab[Title/Abstract])) OR ("durvalumab" [Supplementary Concept])) OR (durvalumab[Title/Abstract]))) AND (((("Randomized Controlled Trials as Topic"[Mesh]) OR (Randomized Controlled Trials as Topic[Title/Abstract])) OR ("Randomized Controlled Trial" [Publication Type])) OR (Randomized Controlled Trial[Title/Abstract]))

Supplementary Table S2: Search information and KMSubtraction implementations

Trial	Comparison	Outcome	Intention-to-treat curve	"Overall" curve	"Subgroup" curve	Derived curves, from "subtracting" "subgroup" curve from "overall" curve	Did the original publication report the corresponding KM curve?	Did the original publication report the corresponding hazard ratio?
CheckMate-649	Nivolumab plus chemotherapy vs. chemotherapy	OS	Intention-to-treat population	All-randomized	PD-L1 CPS≥1	PD-L1 CPS<1	NO	YES
CheckMate-649	Nivolumab plus chemotherapy vs. chemotherapy	PFS	Intention-to-treat population	All-randomized	PD-L1 CPS≥1	PD-L1 CPS<1	NO	YES
CheckMate-649	Nivolumab plus chemotherapy vs. chemotherapy	OS	Intention-to-treat population	PD-L1 CPS≥ 1	PD-L1 CPS≥5	PD-L1 CPS 1-4	NO	YES
CheckMate-649	Nivolumab plus chemotherapy vs. chemotherapy	PFS	Intention-to-treat population	PD-L1 CPS≥ 1	PD-L1 CPS≥5	PD-L1 CPS 1-4	NO	YES

KEYNOTE-859	Pembrolizumab plus chemotherapy vs. chemotherapy	OS	Intention-to-treat population	All-randomized	PD-L1 CPS⩾1	PD-L1 CPS<1	NO	YES
KEYNOTE-859	Pembrolizumab plus chemotherapy vs. chemotherapy	PFS	Intention-to-treat population	All-randomized	PD-L1 CPS⩾1	PD-L1 CPS<1	NO	YES
KEYNOTE-859	Pembrolizumab plus chemotherapy vs. chemotherapy	OS	Intention-to-treat population	PD-L1 CPS⩾ 1	PD-L1 CPS⩾ 10	PD-L1 CPS 1-9	NO	YES
KEYNOTE-859	Pembrolizumab plus chemotherapy vs. chemotherapy	PFS	Intention-to-treat population	PD-L1 CPS⩾ 1	PD-L1 CPS⩾ 10	PD-L1 CPS 1-9	NO	YES
ORIENT-16	Sintilimab plus chemotherapy vs. chemotherapy	OS	Intention-to-treat population	All-randomized	PD-L1 CPS⩾5	PD-L1 CPS<5	NO	YES

ORIENT-16	Sintilimab plus chemotherapy vs. chemotherapy	PFS	Intention-to-treat population	All-randomized	PD-L1 CPS⩾5	PD-L1 CPS<5	NO	YES
RATIONALE-305	Tislelizumab plus chemotherapy vs. chemotherapy	OS	Intention-to-treat population	All-randomized	PD-L1 TAP⩾5%	PD-L1 TAP<5%	NO	NO
RATIONALE-305	Tislelizumab plus chemotherapy vs. chemotherapy	PFS	Intention-to-treat population	All-randomized	P PD-L1 TAP ⩾5%	PD-L1 TAP<5%	NO	NO

Supplementary Table S3: Baseline characteristics and clinical information of the trials

Trial, Year, ClinicalTrials.gov Identifier	Cancer Location	No. of Countries	Key Eligibility Criteria	Intervention Groups, Dosing	PD-L1 expression scoring systems, Assay and Reporting Method	Reported Outcomes (as KM plots)	Derived Outcomes of Interest Unreported as KM Plots in the Original Study	Gastric Cancer or Esophageal Cancer Cases
CheckMate-649 Janjigian,2021 NCT02872116	Gastric, gastroesophageal, and esophageal adenocarcinoma	29 (North America, South America, Europe, Asia, and Oceania)	(1) Age 18 years or older (2) Previously untreated, unresectable, advanced, or metastatic gastric or gastroesophageal junction or esophageal junction adenocarcinoma (3) Evaluable disease as per RECIST 1.1 (4) Adequate organ function (5) Fresh or archival tumor sample (6) ECOG PS 0-1	Chemotherapy XELOX: capecitabine 1,000 mg/m2 twice daily (days 1-14) and oxaliplatin 130 mg/m2 once daily (day 1) each 3-week; or FOLFOX: leucovorin 400 mg/m2 once daily (day 1), FU 400 mg/m2 once daily (day 1), 1,200 mg/m2 once daily (days 1-2) and oxaliplatin 85 mg/m2 once daily (day 1) each 2-week *v* Immunochemotherapy Nivolumab 360 mg each 3-week or 240 mg each 2-week plus chemotherapy (as above)	CPS, IHC 28-8 pharmDx assay (Dako, Santa Clara, CA)	OS:(1) All patients (2) Tumor PD-L1 CPS ≥ 1 (3) Tumor PD-L1 CPS ≥ 5 PFS:(1) All patients (2) Tumor PD-L1 CPS ≥1 (3) Tumor PD-L1 CPS ≥5 All patients' PD-L1 status evaluated	OS ;(1) Tumor PD-L1 CPS <1 (2) Tumor PD-L1 CPS 1-4 PFS:(1) Tumor PD-L1 CPS <1 (2) Tumor PD-L1 CPS 1-4	1581
KEYNOTE-859 Rha ,2023 NCT03675737	Gastric, gastroesophageal adenocarcinoma	30 (North America, South America, Europe, Asia, and Oceania)	(1) Age 18 years or older (2) Previously untreated, unresectable, advanced, or metastatic gastric or gastroesophageal adenocarcinoma (3) Evaluable disease as per RECIST 1.1 (4) Adequate organ function (5) Adequate tumor tissue (6) ECOG PS 0-1	Chemotherapy FU 800 mg/m² once daily (days 1-5) and cisplatin 80 mg/m² once daily (day 1) each 3-week; or Capecitabine 1,000 mg/m² twice daily (days 1-14) and oxaliplatin 130 mg/m² (day 1) each 3-week *v* Immunochemotherapy Pembrolizumab 200 mg each 3-week plus chemotherapy (as above)	CPS, IHC 22C3 pharmDx (Agilent Technologies, Carpinteria, CA)	OS:(1) All patients (2) Tumor PD-L1CPS ≥ 1 (3) Tumor PD-L1CPS ≥10 PFS:(1) All patients (2) Tumor PD-L1 CPS ≥1 (3) Tumor PD-L1 CPS ≥10 All patients' PD-L1 status evaluated	OS :(1) Tumor PD-L1 CPS <1 (2) Tumor PD-L1 CPS 1-9 PFS:(1) Tumor PD-L1 CPS <1 (2) Tumor PD-L1 CPS 1-9	1579

(Supplementary Table 3 continued on following page)

Trial, Year, ClinicalTrials.gov Identifier	Cancer Location	No. of Countries	Key Eligibility Criteria	Intervention Groups, Dosing	PD-L1 expression scoring systems, Assay and Reporting Method	Reported Outcomes (as KM plots)	Derived Outcomes of Interest Unreported as KM Plots in the Original Study	Gastric Cancer or Esophageal Cancer Cases
ORIENT-16<br>Jianming ,2023<br>NCT03745170	Gastric, gastroesophageal adenocarcinoma	1 (China)	(1) Age 18 years or older<br>(2) Previously unresectable, advanced, or metastatic gastric or gastroesophageal junction adenocarcinoma<br>(3) Untreated at least 6 months<br>(4) Evaluable disease as per RECIST 1.1<br>(5) Adequate organ function<br>(6) Adequate tumor tissue<br>(7) ECOG PS 0-1	Chemotherapy<br>XELOX: capecitabine 1,000 mg/m2 twice daily (days 1-14) and oxaliplatin<br>130 mg/m2 once daily (day 1) each 3-week; Capecitabine 1,000 mg/m² twice daily (days 1-14) and oxaliplatin 130 mg/m² (day 1) each 3-week<br>*v*<br>Immunochemotherapy<br>Sintilimab (3mg/kg for body weight <60 kg, 200mg for ≥60 kg) plus chemotherapy (as above)	CPS, IHC 22C3 pharmDx assay (Agilent Technologies, Carpinteria, CA)	OS:(1) All patients<br>(2) Tumor PD-L1 CPS ≥ 5<br>PFS:(1) All patients<br>(2) Tumor PD-L1 CPS ≥5<br>All patients' PD-L1 status evaluated	OS;(1) Tumor PD-L1 CPS <5<br>PFS :(1) Tumor PD-L1 CPS <5	650
RATIONALE-305<br>Markus H.,2024<br>NCT03777657	Gastric, gastroesophageal adenocarcinoma	13 (North America,South America,Europe, Asia,and Oceania)	(1) Age 18 years or older<br>(2) Previously untreated, unresectable, advanced, or metastatic gastric or gastroesophageal junction adenocarcinoma<br>(3) No HER-2 positive disease<br>(3) Evaluable disease as per RECIST 1.1<br>(4) Adequate organ function<br>(5) Fresh or archival tumor sample<br>(6) ECOG PS 0-1	Chemotherapy<br>XELOX: capecitabine 1,000 mg/m2 twice daily (days 1-14) and oxaliplatin<br>130 mg/m2 once daily (day 1) each 3-week; or<br>FP: Cisplatin 80 mg/m² once daily (day 1), FU 800 mg/m2 once daily (day 1-5) each 2-week<br>*v*<br>Immunochemotherapy<br>Tislelizumab 200 mg each 3-week plus chemotherapy (as above)	TAP, VENTANA SP263 IHC Assay (Ventana Medical Systems)	OS:(1) All patients<br>(2) Tumor PD-L1 TAP ≥ 5%<br>PFS:(1) All patients<br>(2) Tumor PD-L1 TAP ≥ 5%<br>All patients' PD-L1 status evaluated	OS:(1) Tumor PD-L1 TAP < 5%<br>PFS:(2) Tumor PD-L1 TAP < 5%	997

Abbreviations: CPS, combined positive score; ECOG PS, Eastern Cooperative Oncology Group performance status; XELOX, oxaliplatin and capecitabine; FOLFOX, folinic acid, fluorouracil, and oxaliplatin; PD-L1, programed death-ligand 1; IHC, immunohistochemistry; KM, Kaplan-Meier; OS, overall survival; PFS, progression-free survival; FU, fluorouracil; FP, cisplatin and fluorouracil; TAP, tumor area positivity

Supplementary Table S4: Reconstructed curves Compared with original curves

Study, outcome, cohort	Original	Reconstructed
**CheckMate-649, overall survival, overall patients**	The original figure can be located within the primary trial manuscript.<br><br>Janjigian YY, Shitara K, Moehler M, et al: First-line nivolumab plus chemotherapy versus chemotherapy alone for advanced gastric, gastro-oesophageal junction, and oesophageal adenocarcinoma (CheckMate 649): A randomised, open-label phase 3 trial. Lancet 398:27-40, 2021<br>Figure 2C	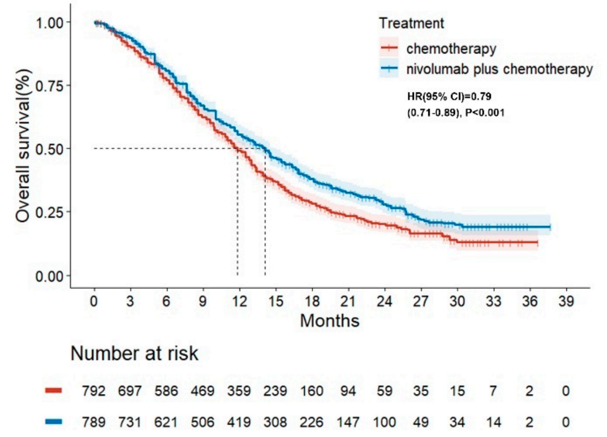
**CheckMate-649, overall survival, CPS≥1**	The original figure can be located within the primary trial manuscript.<br><br>Janjigian YY, Shitara K, Moehler M, et al: First-line nivolumab plus chemotherapy versus chemotherapy alone for advanced gastric, gastro-oesophageal junction, and oesophageal adenocarcinoma (CheckMate 649): A randomised, open-label phase 3 trial. Lancet 398:27-40, 2021<br>Figure 2B	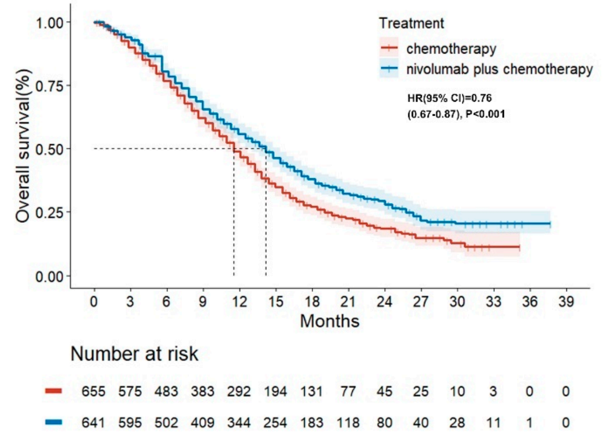

**CheckMate-649, overall survival, CPS≥5**	The original figure can be located within the primary trial manuscript.<br><br>Janjigian YY, Shitara K, Moehler M, et al: First-line nivolumab plus chemotherapy versus chemotherapy alone for advanced gastric, gastro-oesophageal junction, and oesophageal adenocarcinoma (CheckMate 649): A randomised, open-label phase 3 trial. Lancet 398:27-40, 2021<br>Figure 2A	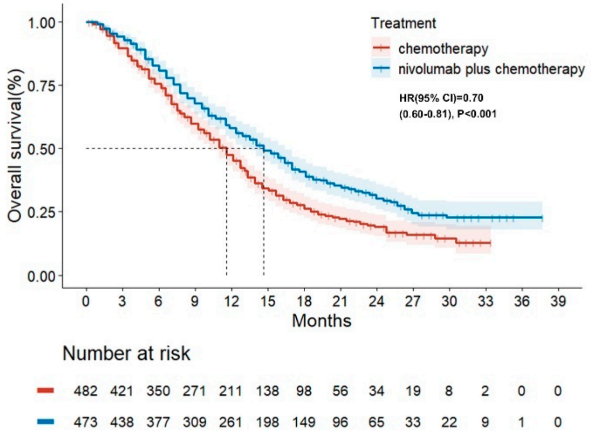
**CheckMate-649, progression free survival, overall patients**	The original figure can be located within the primary trial manuscript.<br><br>Janjigian YY, Shitara K, Moehler M, et al: First-line nivolumab plus chemotherapy versus chemotherapy alone for advanced gastric, gastro-oesophageal junction, and oesophageal adenocarcinoma (CheckMate 649): A randomised, open-label phase 3 trial. Lancet 398:27-40, 2021<br>Figure 3C	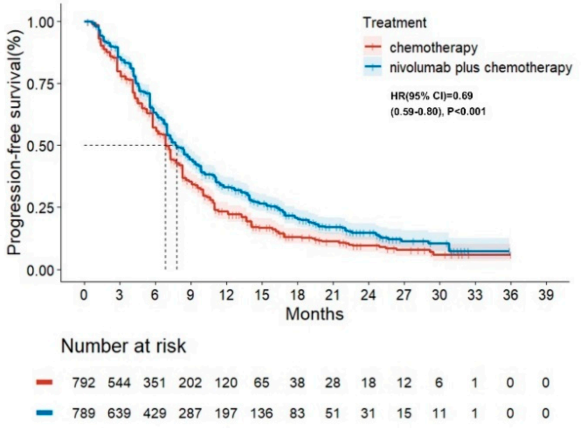

<table>
<tr>
<td>CheckMate-649, progression free survival, CPS≥1</td>
<td>The original figure can be located within the primary trial manuscript.

Janjigian YY, Shitara K, Moehler M, et al: First-line nivolumab plus chemotherapy versus chemotherapy alone for advanced gastric, gastro-oesophageal junction, and oesophageal adenocarcinoma (CheckMate 649): A randomised, open-label phase 3 trial. Lancet 398:27-40, 2021
Figure 3B</td>
<td>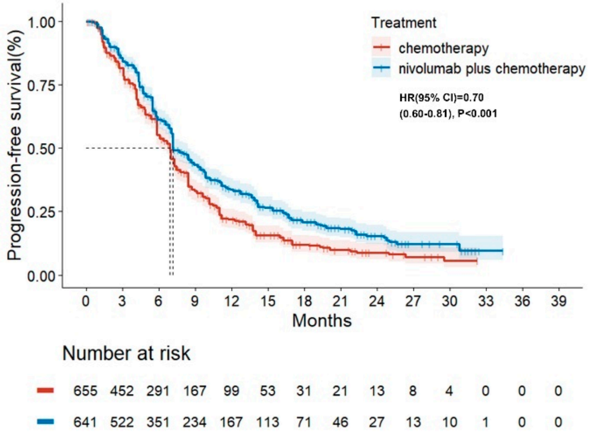
</td>
</tr>
<tr>
<td>CheckMate-649, progression free survival, CPS≥5</td>
<td>The original figure can be located within the primary trial manuscript.

Janjigian YY, Shitara K, Moehler M, et al: First-line nivolumab plus chemotherapy versus chemotherapy alone for advanced gastric, gastro-oesophageal junction, and oesophageal adenocarcinoma (CheckMate 649): A randomised, open-label phase 3 trial. Lancet 398:27-40, 2021
Figure 3A</td>
<td>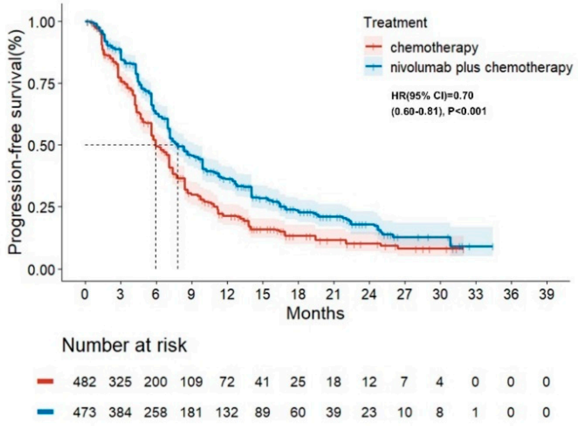
</td>
</tr>
</table>

<table>
<tr>
<td>KEYNOTE-859, overall survival, overall patients</td>
<td>The original figure can be located within the primary trial manuscript.

Rha SY, Oh DY, Yañez P, et al: Pembrolizumab plus chemotherapy versus placebo plus chemotherapy for HER2-negative advanced gastric cancer (KEYNOTE-859): a multicentre, randomised, double-blind, phase 3 trial. Lancet Oncol 24: 1181–95, 2023
Figure 2A</td>
<td>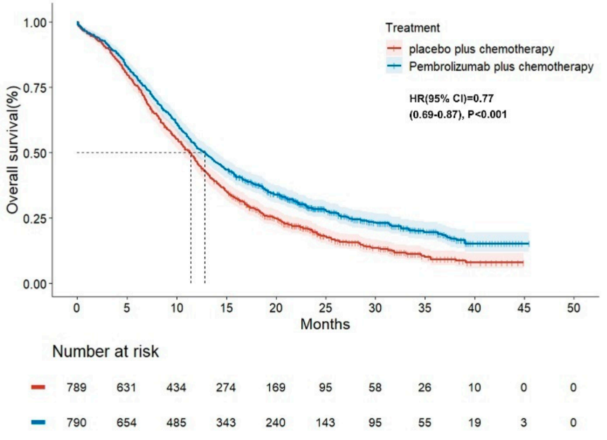
</td>
</tr>
<tr>
<td>KEYNOTE-859, overall survival, CPS≥1</td>
<td>The original figure can be located within the primary trial manuscript.

Rha SY, Oh DY, Yañez P, et al: Pembrolizumab plus chemotherapy versus placebo plus chemotherapy for HER2-negative advanced gastric cancer (KEYNOTE-859): a multicentre, randomised, double-blind, phase 3 trial. Lancet Oncol 24: 1181–95, 2023
Figure 2B</td>
<td>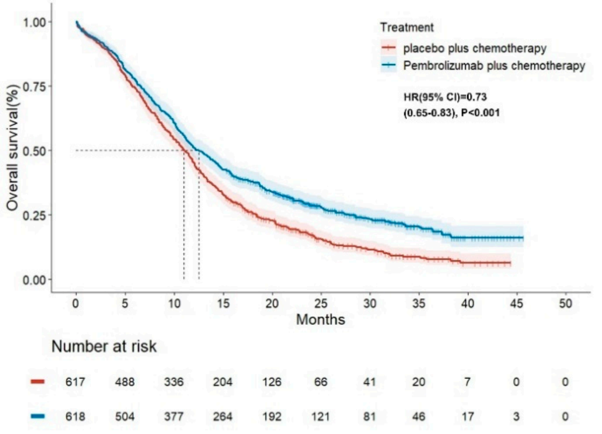
</td>
</tr>
</table>

**KEYNOTE-859, overall survival, CPS≥10**	The original figure can be located within the primary trial manuscript.<br><br>Rha SY, Oh DY, Yañez P, et al: Pembrolizumab plus chemotherapy versus placebo plus chemotherapy for HER2-negative advanced gastric cancer (KEYNOTE-859): a multicentre, randomised, double-blind, phase 3 trial. Lancet Oncol 24: 1181–95, 2023<br>Figure 2C	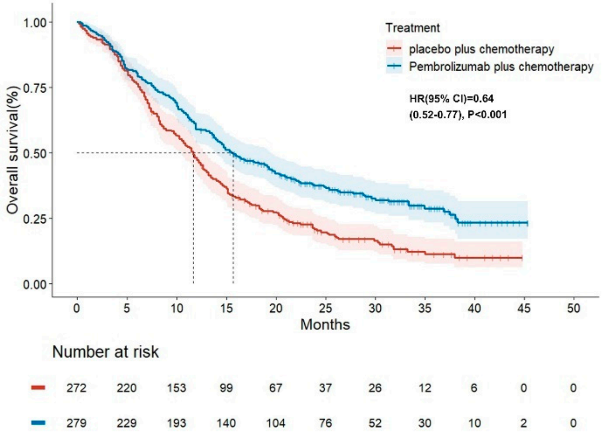
**KEYNOTE-859, progression free survival, overall patients**	The original figure can be located within the primary trial manuscript.<br><br>Rha SY, Oh DY, Yañez P, et al: Pembrolizumab plus chemotherapy versus placebo plus chemotherapy for HER2-negative advanced gastric cancer (KEYNOTE-859): a multicentre, randomised, double-blind, phase 3 trial. Lancet Oncol 24: 1181–95, 2023<br>Figure 3A	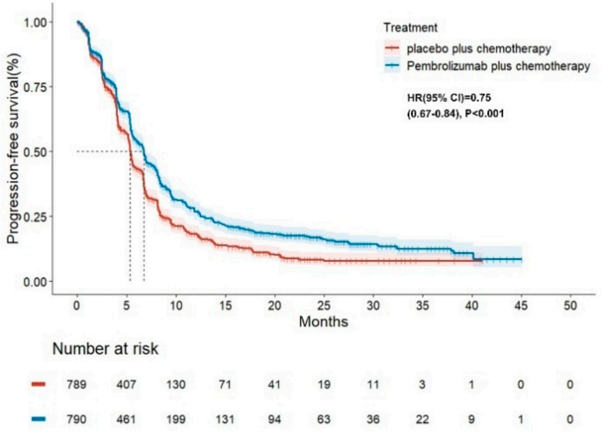

**KEYNOTE-859, progression free survival, CPS≥1**	The original figure can be located within the primary trial manuscript.<br><br>Rha SY, Oh DY, Yañez P, et al: Pembrolizumab plus chemotherapy versus placebo plus chemotherapy for HER2-negative advanced gastric cancer (KEYNOTE-859): a multicentre, randomised, double-blind, phase 3 trial. Lancet Oncol 24: 1181–95, 2023<br>Figure 3A	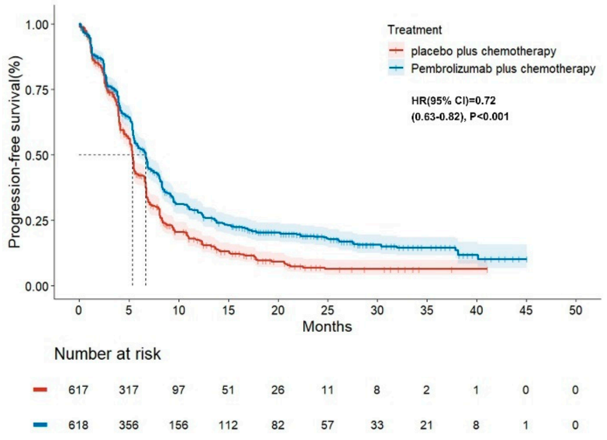
**KEYNOTE-859, progression free survival, CPS≥10**	The original figure can be located within the primary trial manuscript.<br><br>Rha SY, Oh DY, Yañez P, et al: Pembrolizumab plus chemotherapy versus placebo plus chemotherapy for HER2-negative advanced gastric cancer (KEYNOTE-859): a multicentre, randomised, double-blind, phase 3 trial. Lancet Oncol 24: 1181–95, 2023<br>Figure 3B	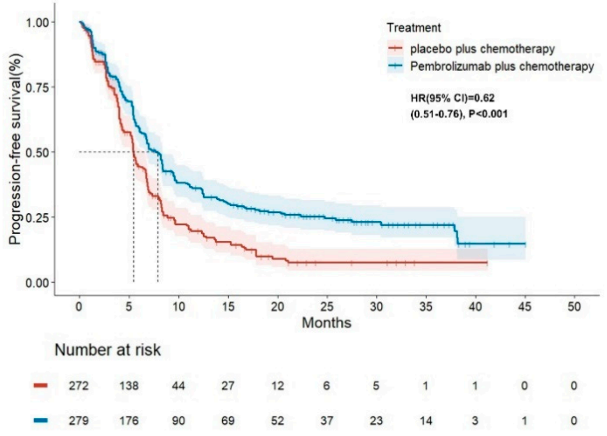

<table>
<tr>
<td>ORIENT-16, overall survival, overall patients</td>
<td>The original figure can be located within the primary trial manuscript.

Xu JM, Jiang HP, Pan YY, et al: Sintilimab Plus Chemotherapy for Unresectable Gastric or Gastroesophageal Junction CancerThe ORIENT-16 Randomized Clinical Trial. JAMA 330(21):2064-2074, 2023
Figure 2B</td>
<td>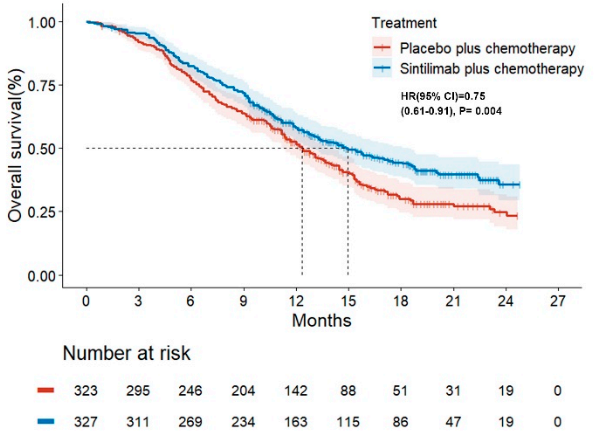
</td>
</tr>
<tr>
<td>ORIENT-16, overall survival, CPS≥5</td>
<td>The original figure can be located within the primary trial manuscript.

Xu JM, Jiang HP, Pan YY, et al: Sintilimab Plus Chemotherapy for Unresectable Gastric or Gastroesophageal Junction CancerThe ORIENT-16 Randomized Clinical Trial. JAMA 330(21):2064-2074, 2023
Figure 2A</td>
<td>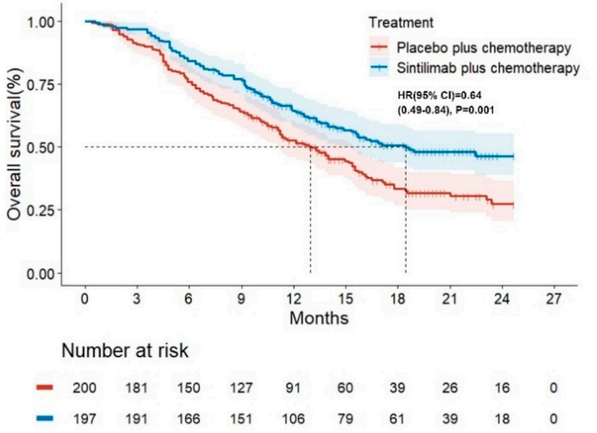
</td>
</tr>
</table>

<table>
<tr>
<td>ORIENT-16, progression free survival, overall patients (Provided in supplement 3 of main paper)</td>
<td>The original figure can be located within the primary trial manuscript.

Xu JM, Jiang HP, Pan YY, et al: Sintilimab Plus Chemotherapy for Unresectable Gastric or Gastroesophageal Junction CancerThe ORIENT-16 Randomized Clinical Trial. JAMA 330(21):2064-2074, 2023
eFigure 1B</td>
<td>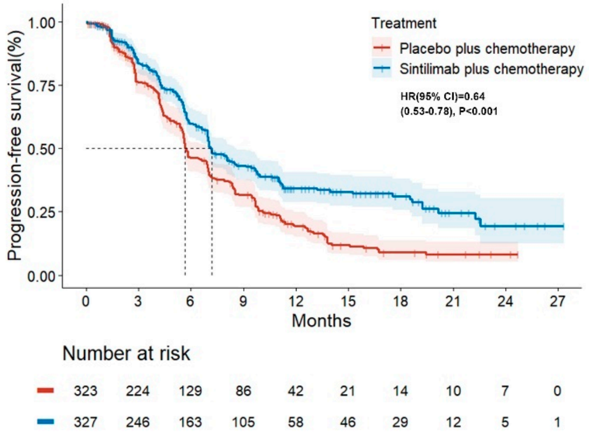
</td>
</tr>
<tr>
<td>ORIENT-16, progression free survival, CPS≥5 (Provided in supplement 3 of main paper)</td>
<td>The original figure can be located within the primary trial manuscript.

Xu JM, Jiang HP, Pan YY, et al: Sintilimab Plus Chemotherapy for Unresectable Gastric or Gastroesophageal Junction CancerThe ORIENT-16 Randomized Clinical Trial. JAMA 330(21):2064-2074, 2023
eFigure 1A</td>
<td>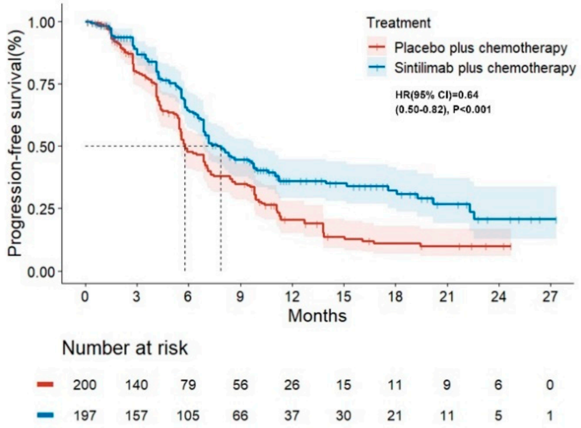
</td>
</tr>
</table>

<table>
<tr>
<td>RATIONALE-305, overall survival, overall patients</td>
<td>The original figure can be located within the primary trial manuscript.

Qiu MZ et al. “Tislelizumab plus chemotherapy versus placebo plus chemotherapy as first line treatment for advanced gastric or gastro-oesophageal junction adenocarcinoma: RATIONALE-305 randomised, double blind, phase 3 trial.” BMJ (Clinical research ed.) vol. 385 e078876. 28 May. 2024
Figure 2B</td>
<td>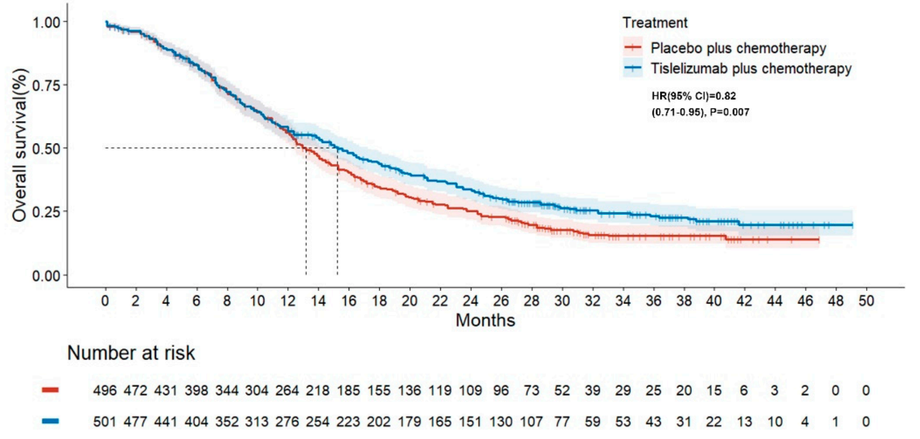
</td>
</tr>
<tr>
<td>RATIONALE-305, overall survival, TAP≥5%</td>
<td>The original figure can be located within the primary trial manuscript.

Qiu MZ et al. “Tislelizumab plus chemotherapy versus placebo plus chemotherapy as first line treatment for advanced gastric or gastro-oesophageal junction adenocarcinoma: RATIONALE-305 randomised, double blind, phase 3 trial.” BMJ (Clinical research ed.) vol. 385 e078876. 28 May. 2024
Figure 2A</td>
<td>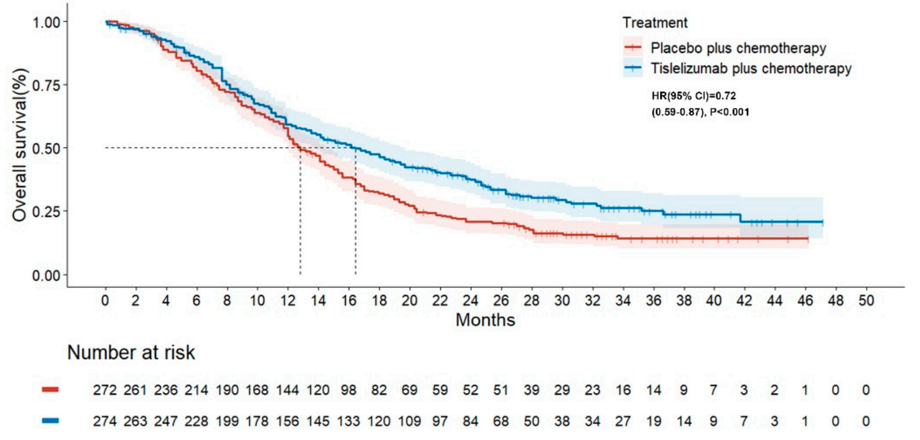
</td>
</tr>
</table>

<table>
<tr>
<td>RATIONALE-305, progression free survival, overall patients</td>
<td>The original figure can be located within the primary trial manuscript.

Qiu MZ et al. “Tislelizumab plus chemotherapy versus placebo plus chemotherapy as first line treatment for advanced gastric or gastro-oesophageal junction adenocarcinoma: RATIONALE-305 randomised, double blind, phase 3 trial.” BMJ (Clinical research ed.) vol. 385 e078876. 28 May. 2024
Figure 3B</td>
<td>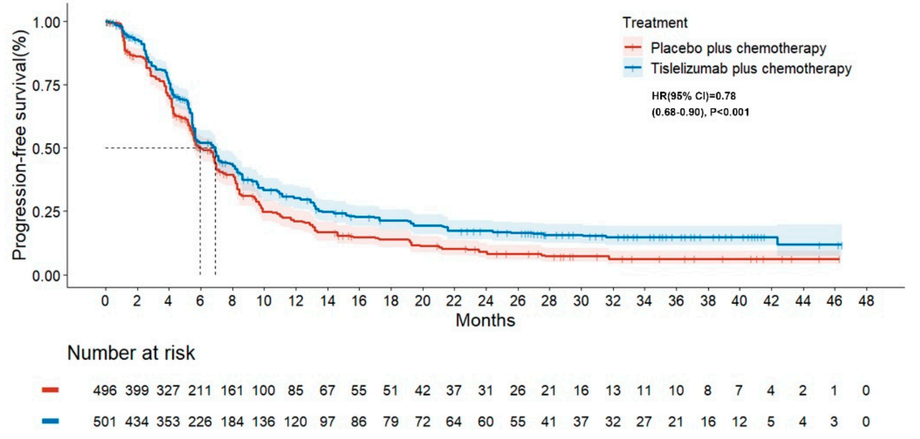
</td>
</tr>
<tr>
<td>RATIONALE-305, progression free survival, TAP≥5%</td>
<td>Qiu MZ et al. “Tislelizumab plus chemotherapy versus placebo plus chemotherapy as first line treatment for advanced gastric or gastro-oesophageal junction adenocarcinoma: RATIONALE-305 randomised, double blind, phase 3 trial.” BMJ (Clinical research ed.) vol. 385 e078876. 28 May. 2024
Figure 3A</td>
<td>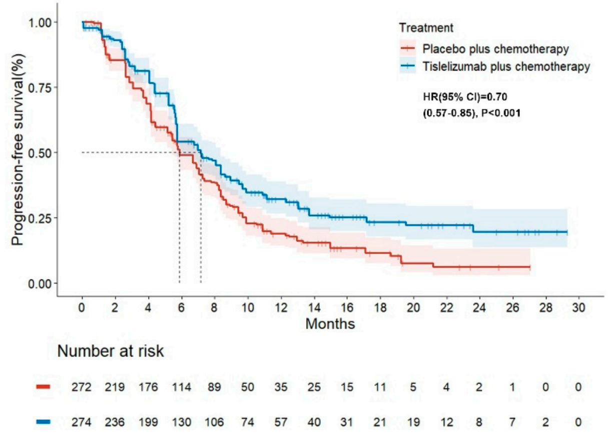
</td>
</tr>
</table>

Abbreviations: CPS, combined positive score; TAP, tumor area positivity; CI, confidence interval; HR, hazard ratioSupplementary

Supplementary Table S5: Example of KMSubtraction outcomes compared with reported HRs for low PD-L1 expression subgroups

Trial, comparison	PD-L1 expression subgroup, Outcome	Reported Hazard ratio (95%CI)	p-value	KMSubtraction with bipartite matching curve, hazard ratio (95%CI)
CheckMate-649 Nivolumab plus chemotherapy vs chemotherapy	PD-L1 CPS<1, OS	0.92 (0.70-1.23)	NR	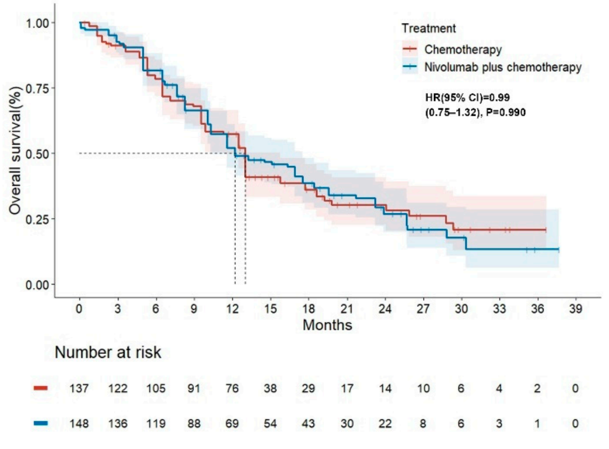
CheckMate-649 Nivolumab plus chemotherapy vs chemotherapy	PD-L1 CPS<1, PFS	0.93 (0.69-1.26)	NR	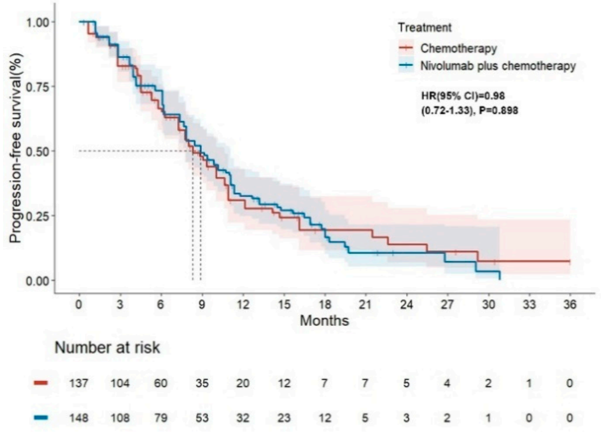

KEYNOTE-859 Pembrolizumab plus chemotherapy vs chemotherapy	PD-L1 CPS<1, OS	0·92 (0·73–1.17)	NR	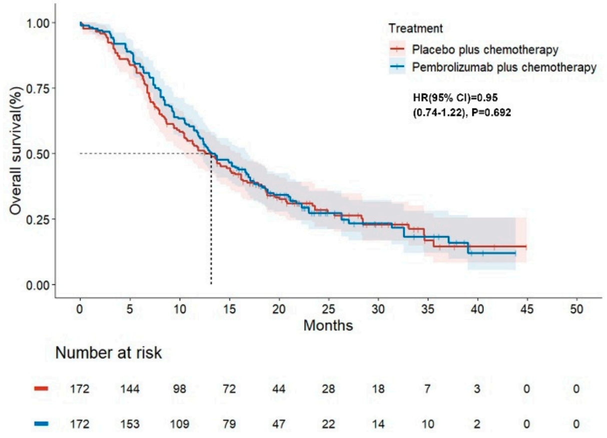
KEYNOTE-859 Pembrolizumab plus chemotherapy vs chemotherapy	PD-L1 CPS<1, PFS	0·90 (0·70–1.15)	NR	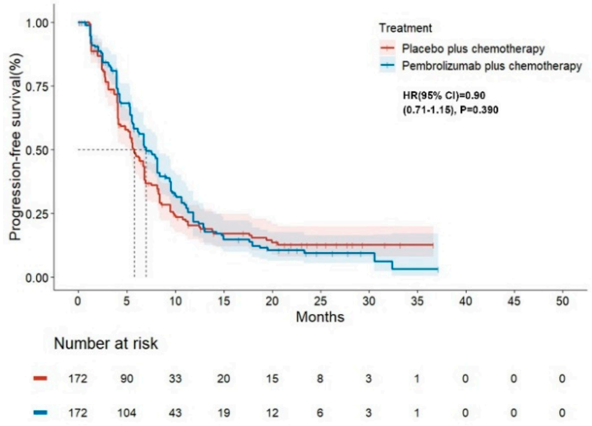

Abbreviations: CI, confidence interval; PD-L1, programmed death ligand 1; NR, not reported; CPS, combined positive score

Supplementary Table S6: Evaluation of KMSubtraction bipartite matching

CheckMate-649 OS PD-L1 CPS≥1 in chemotherapy group<br>Utilizing empirical cumulative distributions and Bland-Altman plots to investigate discrepancies among matched pairs	Kaplan Meier Curves and Cox-proportional hazard's model comparing matched pairs

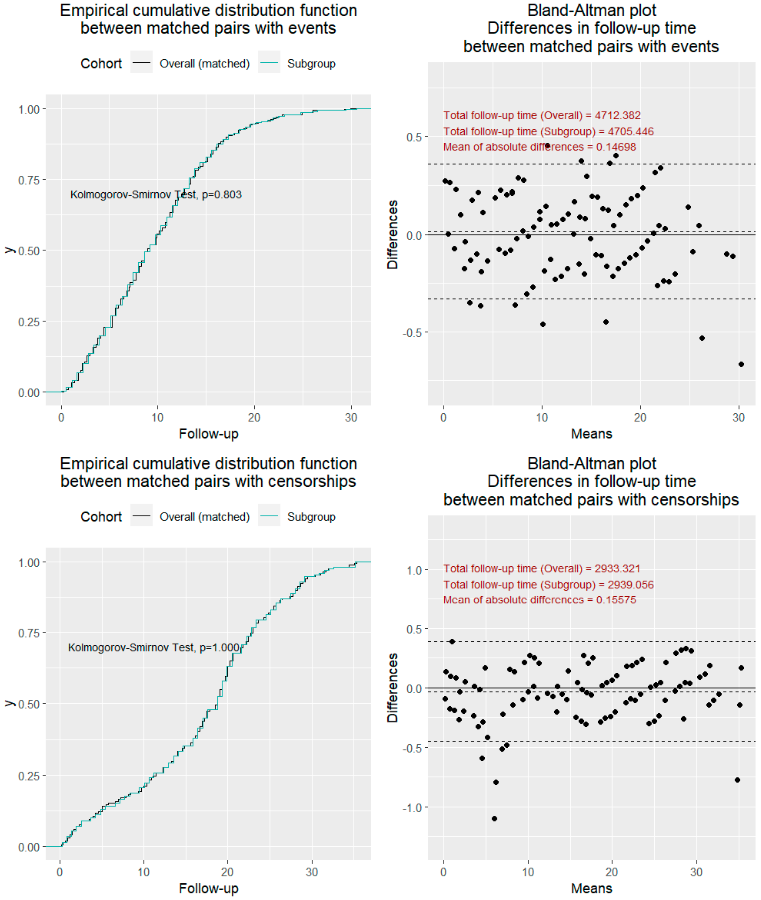

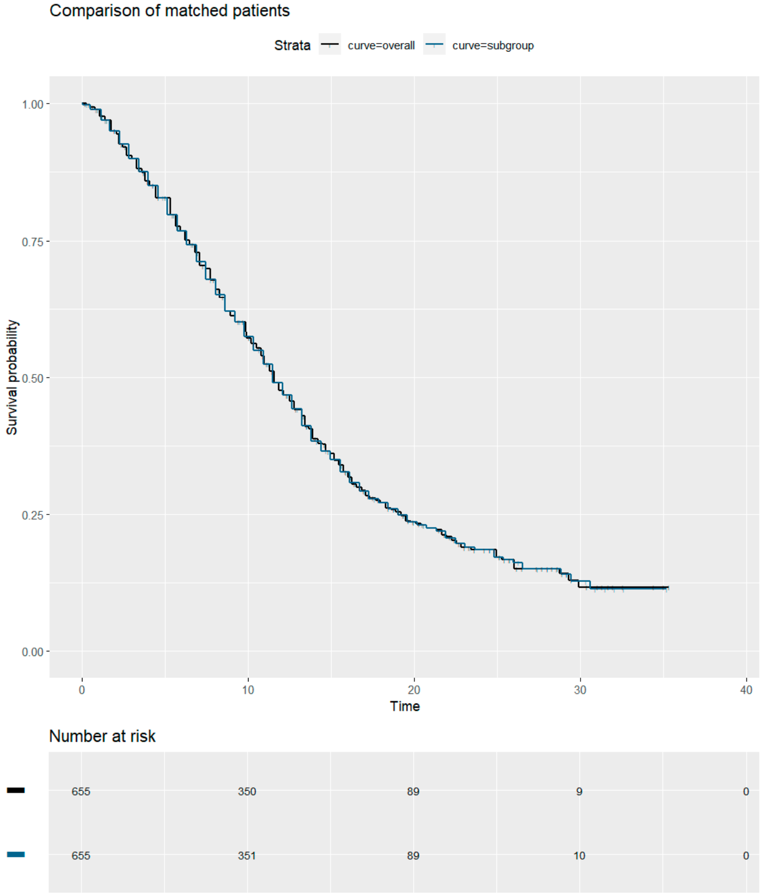

CheckMate-649 PFS PD-L1 CPS≥1 in chemotherapy group<br>Utilizing empirical cumulative distributions and Bland-Altman plots to investigate discrepancies among matched pairs	Kaplan Meier Curves and Cox-proportional hazard's model comparing matched pairs

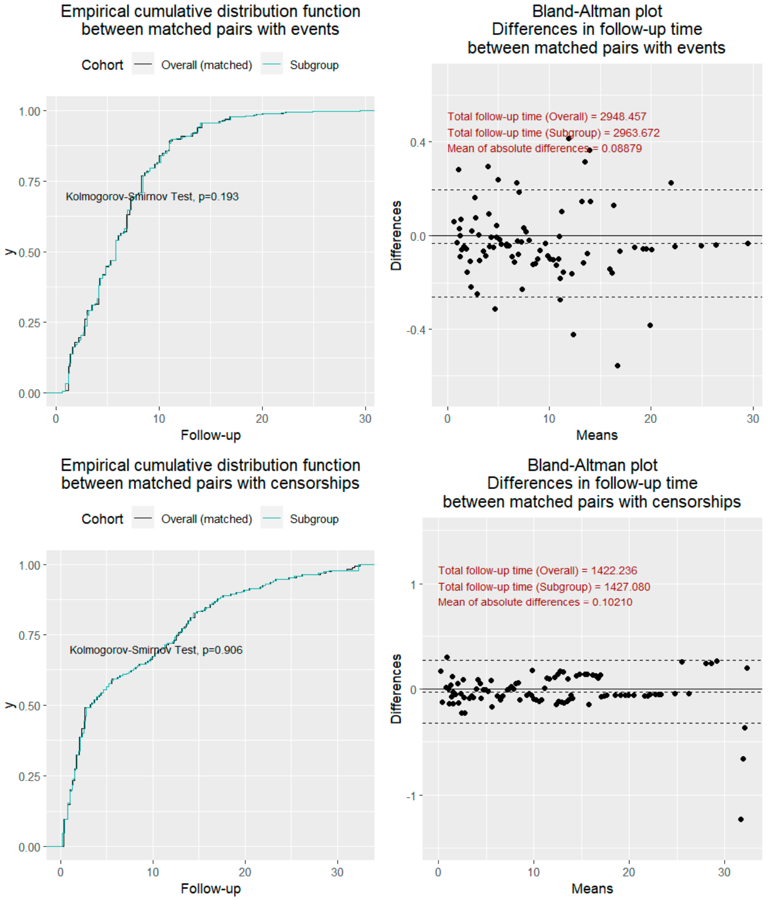

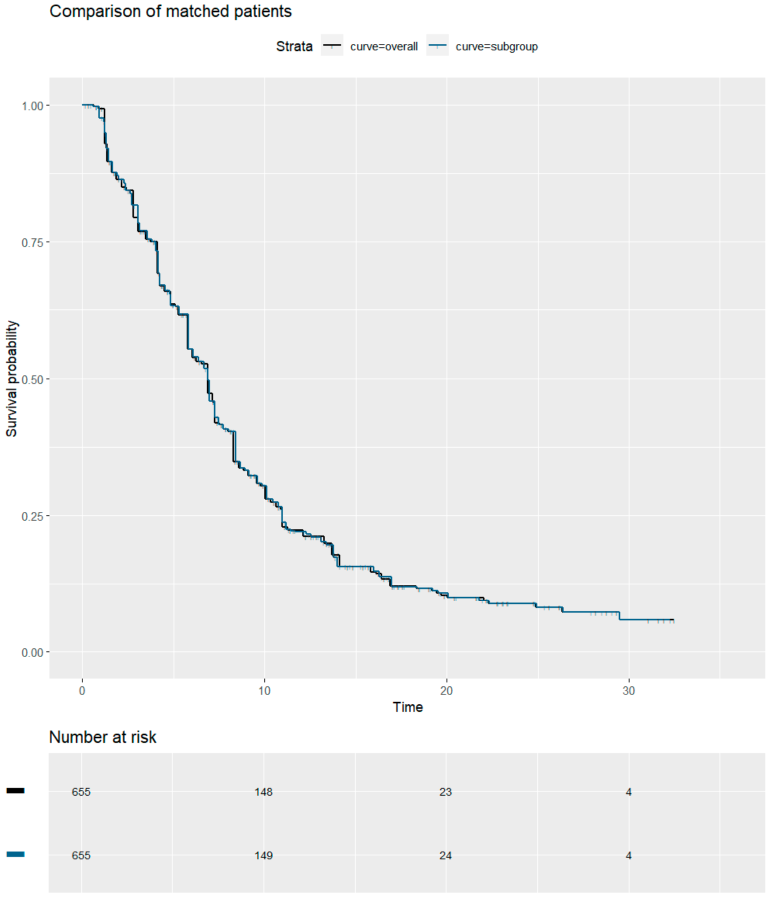

CheckMate-649 OS PD-L1 CPS≥1 in Nivolumab plus chemotherapy group Utilizing empirical cumulative distributions and Bland-Altman plots to investigate discrepancies among matched pairs	Kaplan Meier Curves and Cox-proportional hazard's model comparing matched pairs

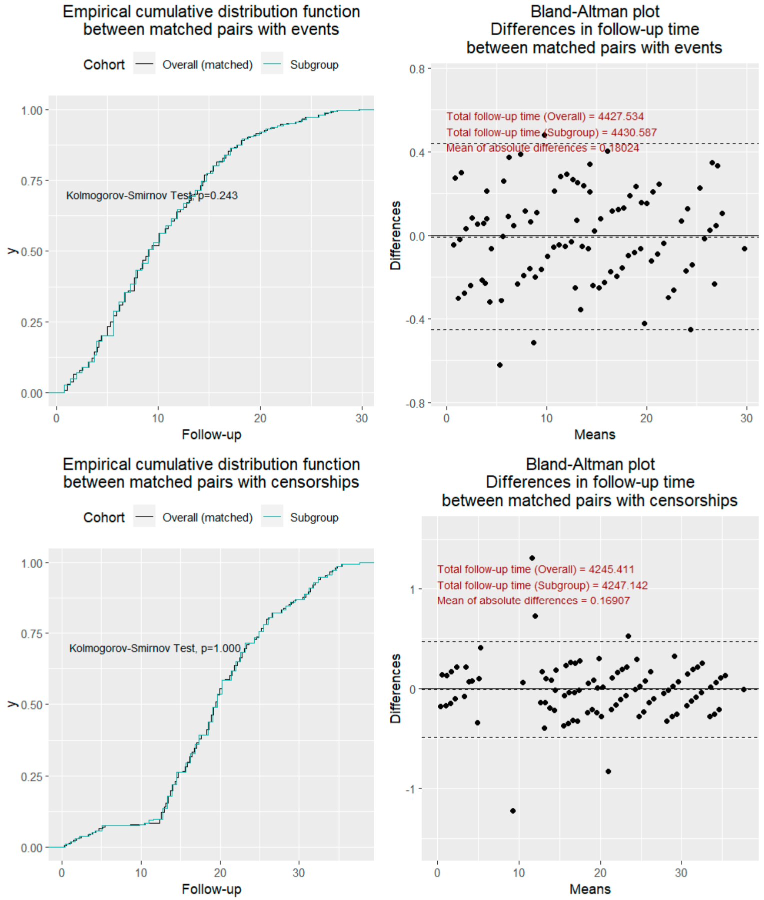

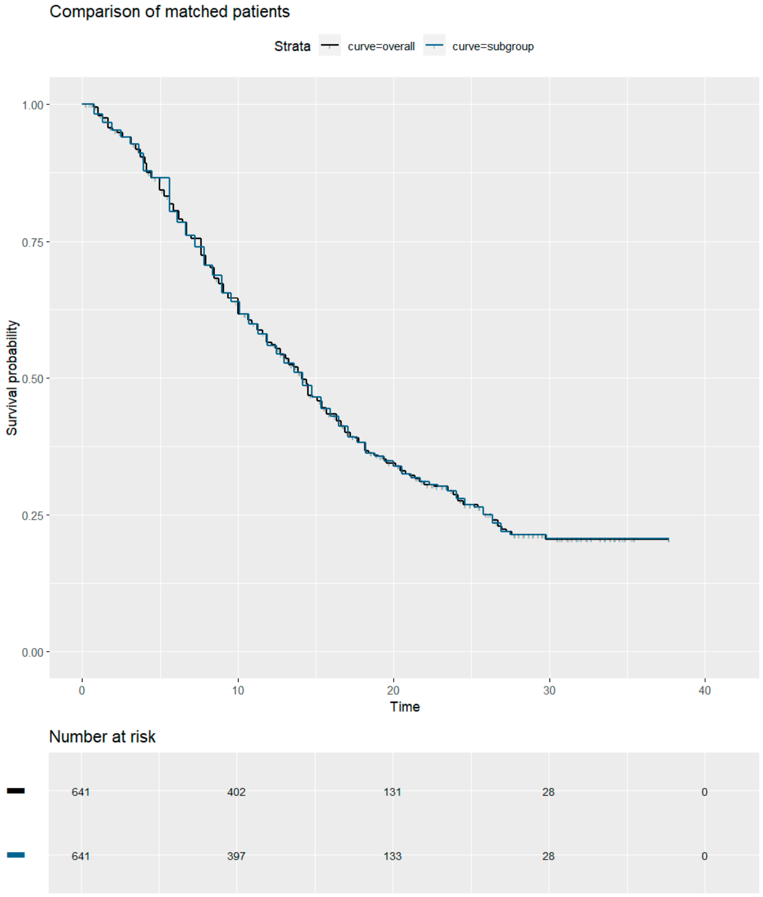

CheckMate-649 PFS PD-L1 CPS≥1 in Nivolumab plus chemotherapy group Utilizing empirical cumulative distributions and Bland-Altman plots to investigate discrepancies among matched pairs	Kaplan Meier Curves and Cox-proportional hazard's model comparing matched pairs

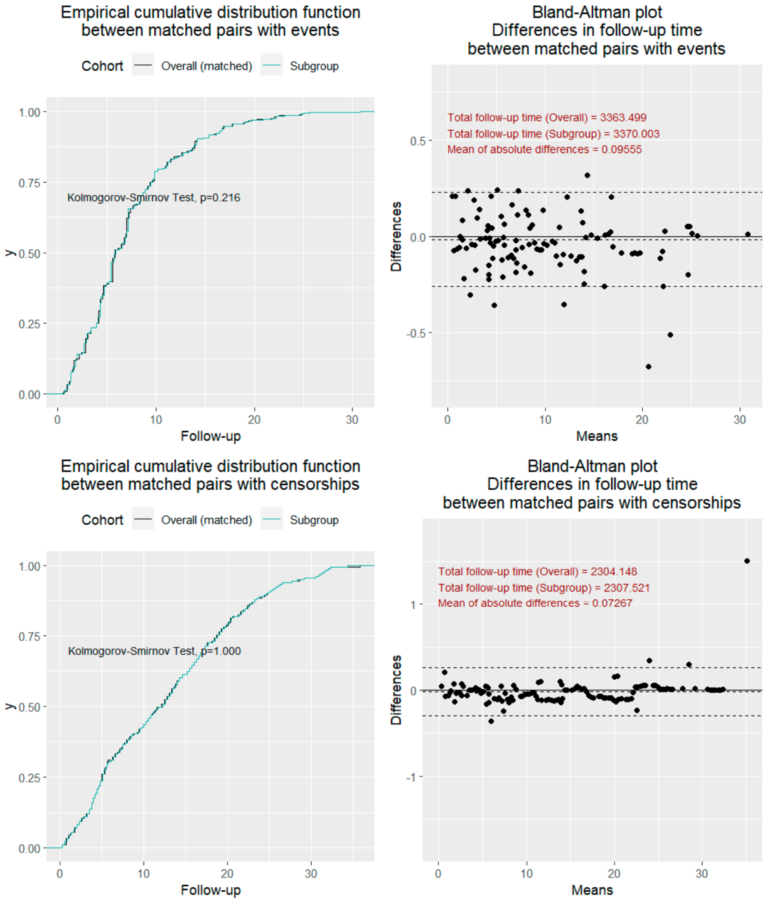

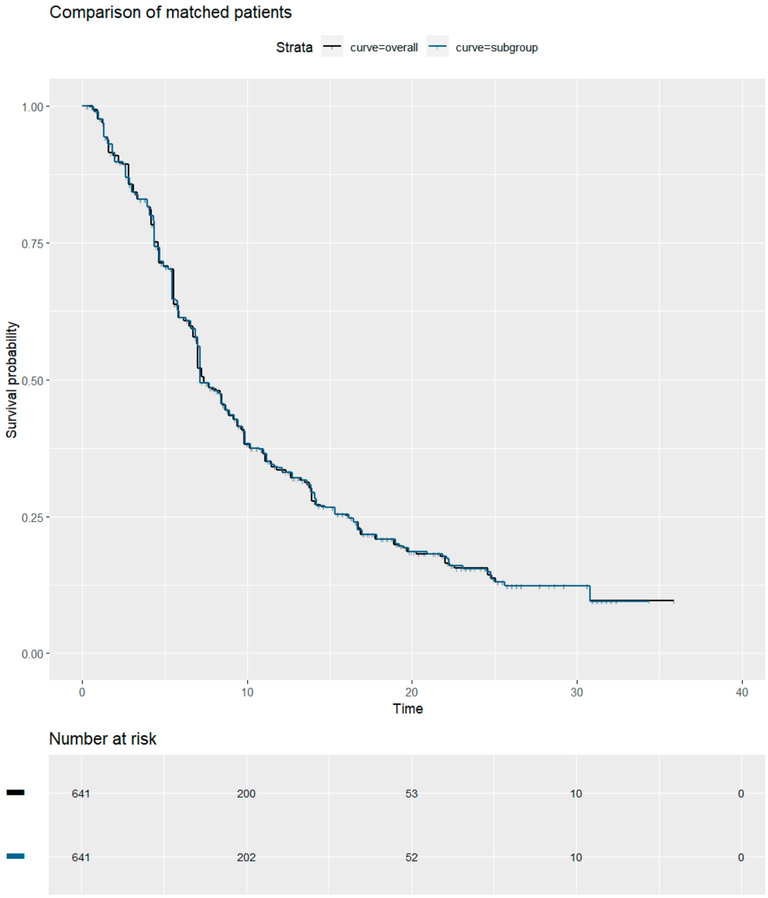

KEYNOTE-859 OS PD-L1 CPS≥1 in chemotherapy group<br>Utilizing empirical cumulative distributions and Bland-Altman plots to investigate discrepancies among matched pairs	Kaplan Meier Curves and Cox-proportional hazard’s model comparing matched pairs

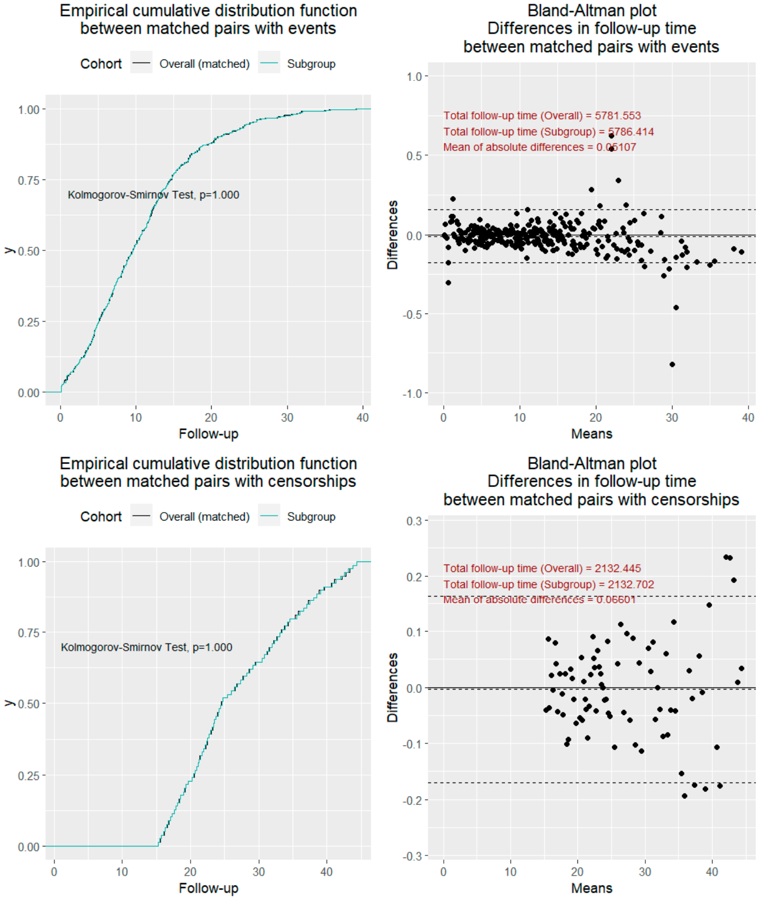

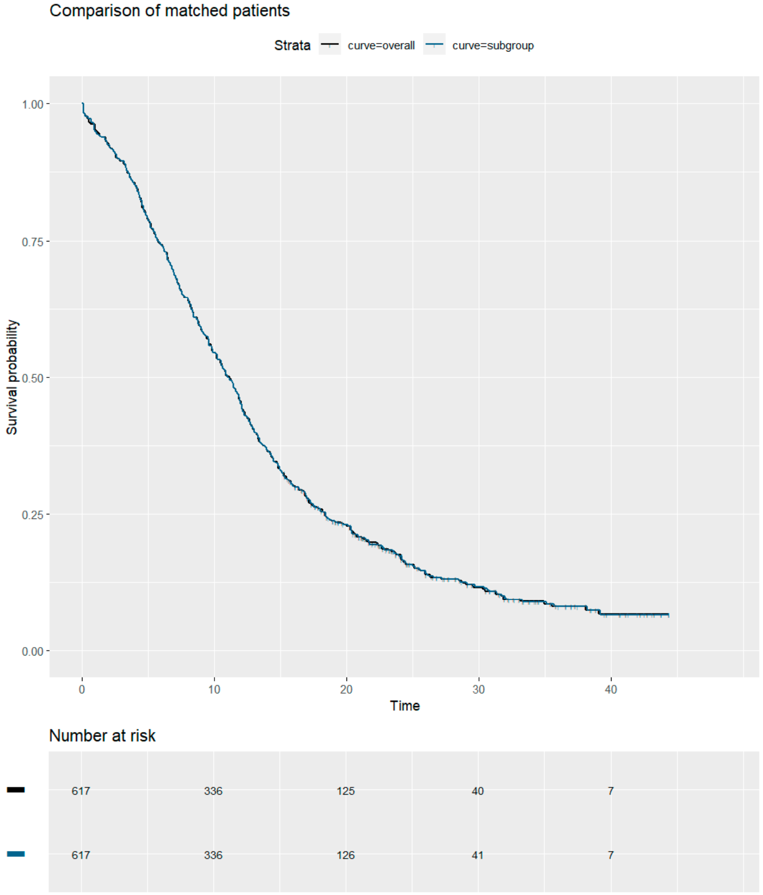

KEYNOTE-859 PFS PD-L1 CPS≥1 in chemotherapy group<br>Utilizing empirical cumulative distributions and Bland-Altman plots to investigate discrepancies among matched pairs	Kaplan Meier Curves and Cox-proportional hazard's model comparing matched pairs

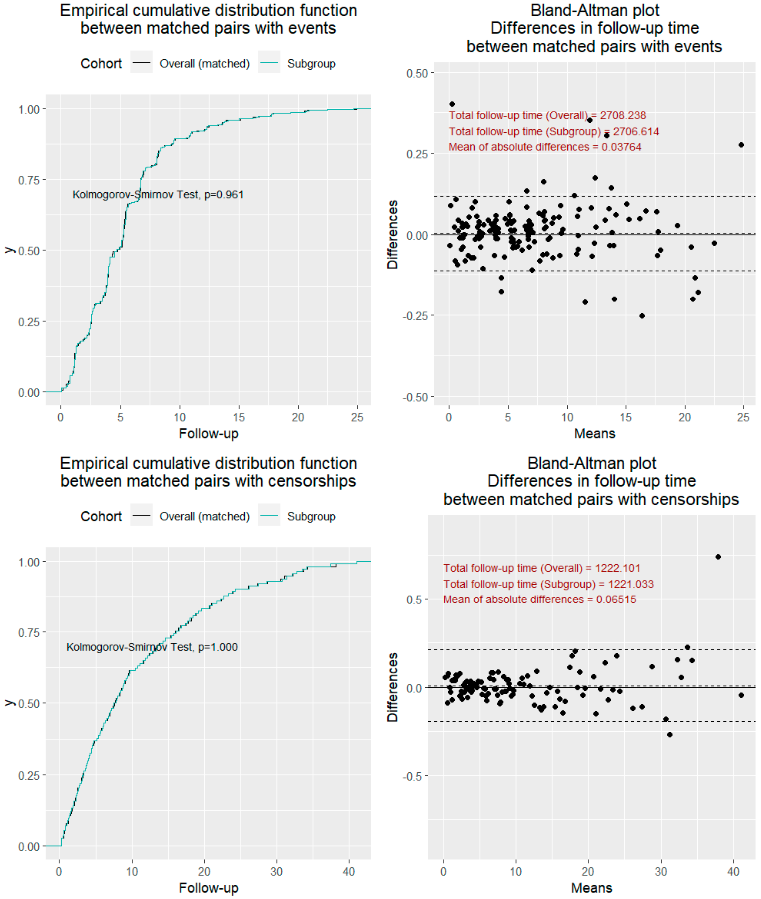

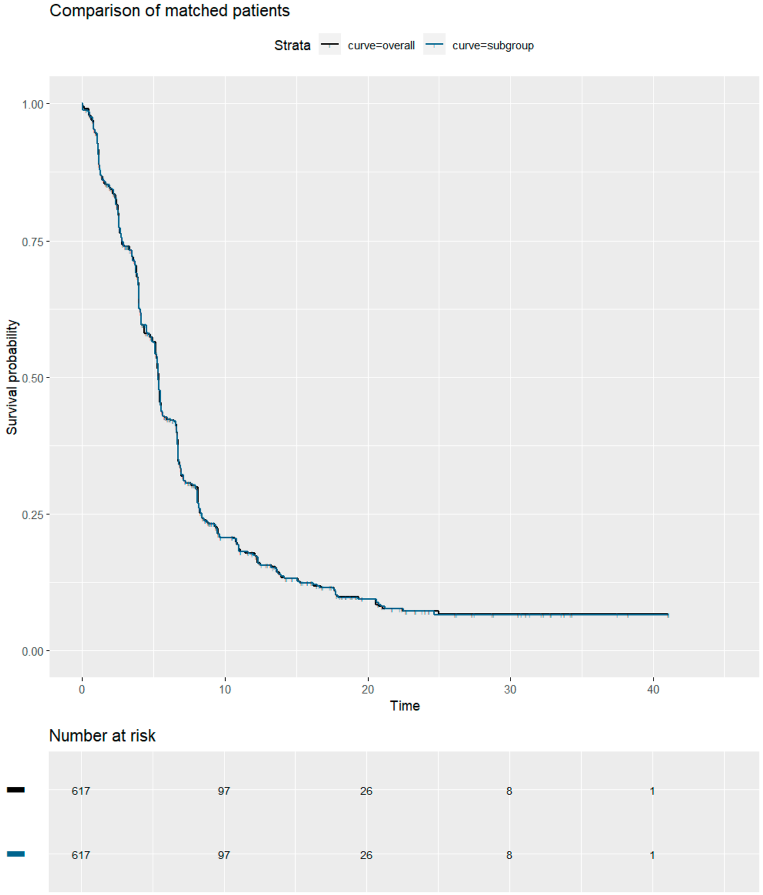

KEYNOTE-859 OS PD-L1 CPS≥1 in Pembrolizumab plus chemotherapy group<br>Utilizing empirical cumulative distributions and Bland-Altman plots to investigate discrepancies among matched pairs	Kaplan Meier Curves and Cox-proportional hazard's model comparing matched pairs
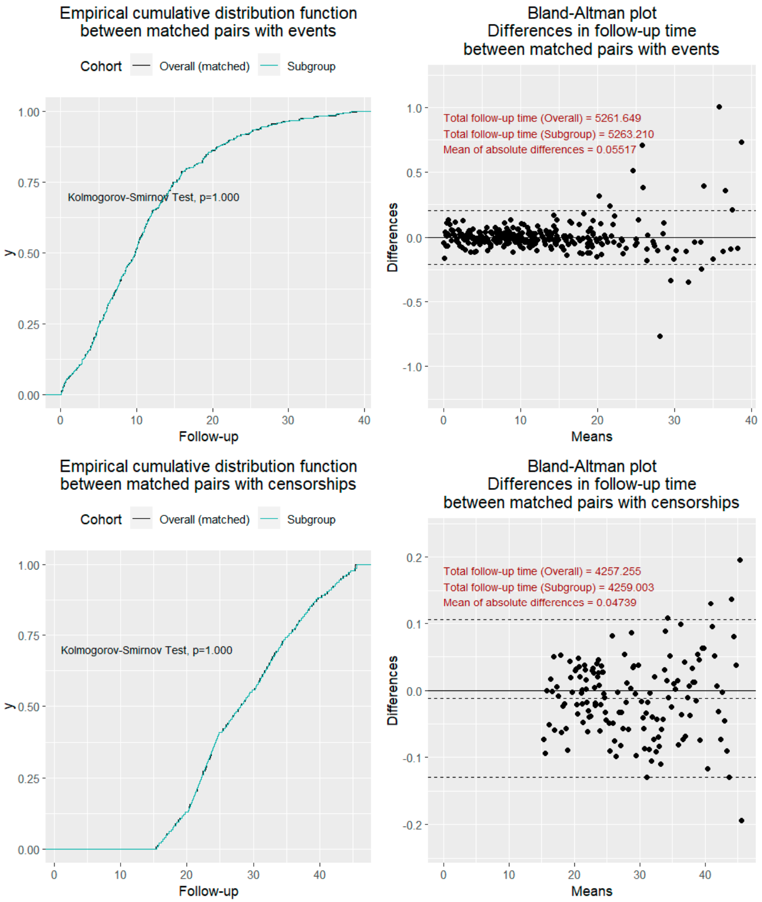	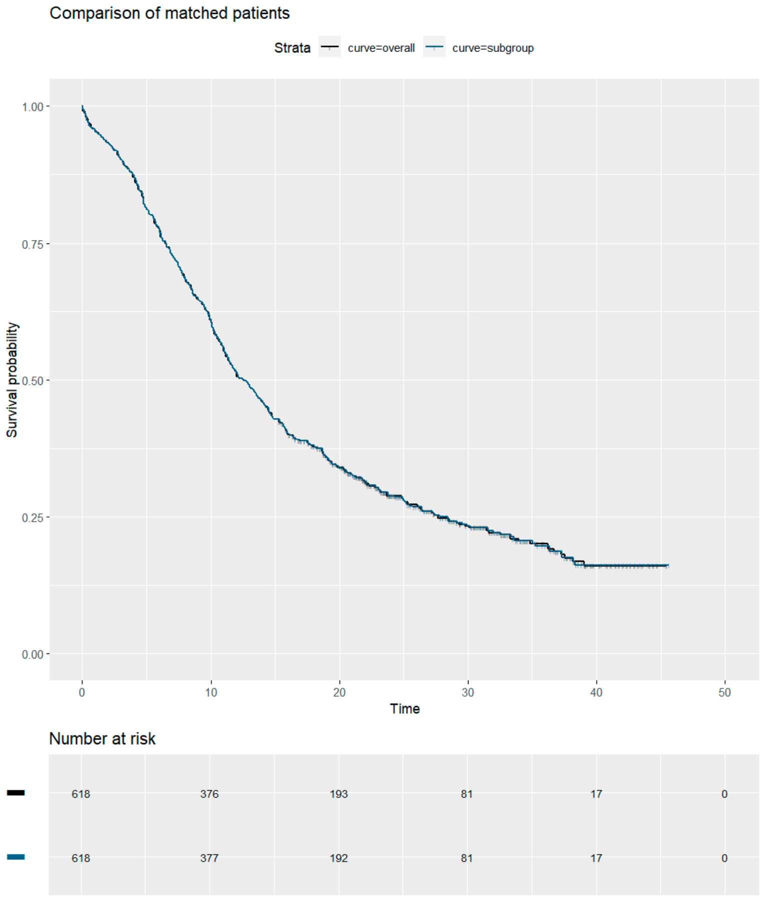

**KEYNOTE-859 PFS PD-L1 CPS≥1 in Pembrolizumab plus chemotherapy group**<br>**Utilizing empirical cumulative distributions and Bland-Altman plots to investigate discrepancies among matched pairs**	**Kaplan Meier Curves and Cox-proportional hazard’s model comparing matched pairs**
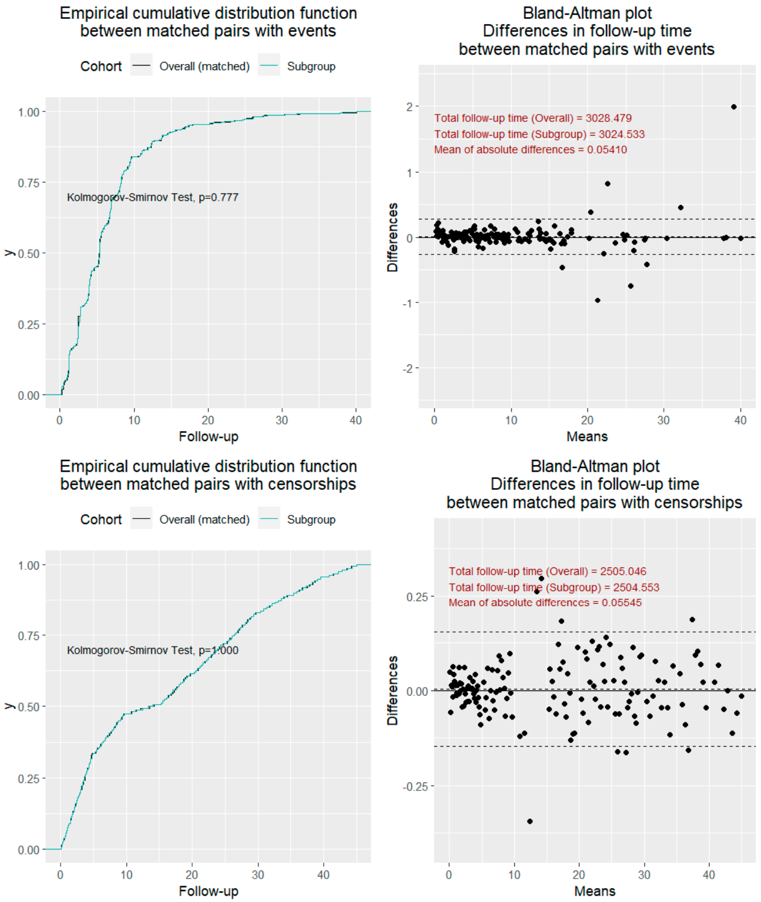	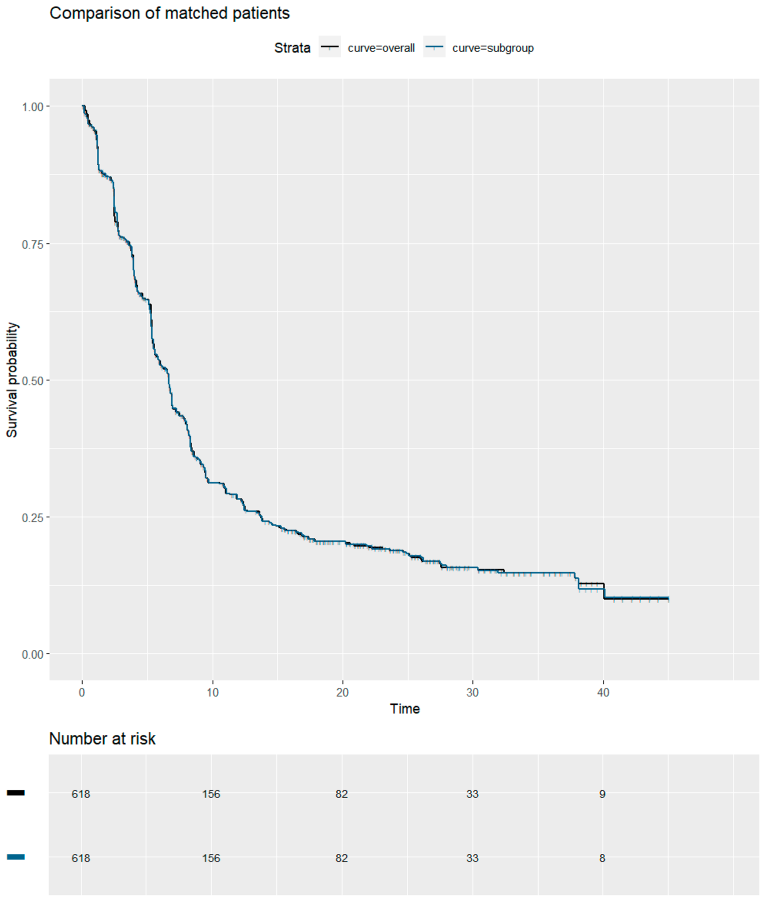

ORIENT-16 OS PD-L1 CPS≥5 in chemotherapy group<br>Utilizing empirical cumulative distributions and Bland-Altman plots to investigate discrepancies among matched pairs	Kaplan Meier Curves and Cox-proportional hazard's model comparing matched pairs

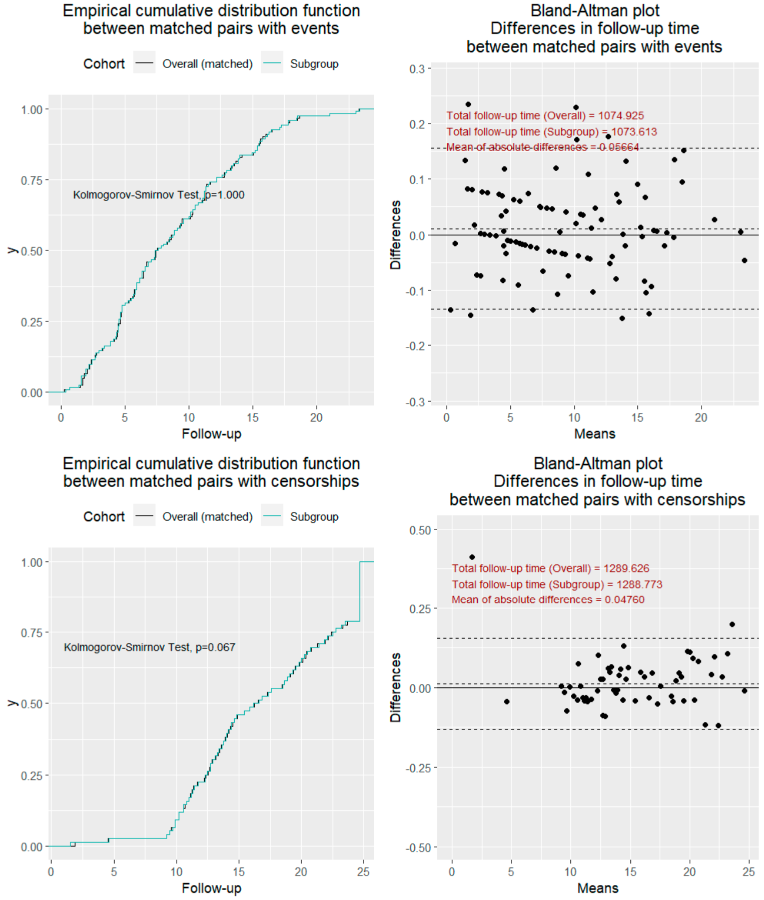

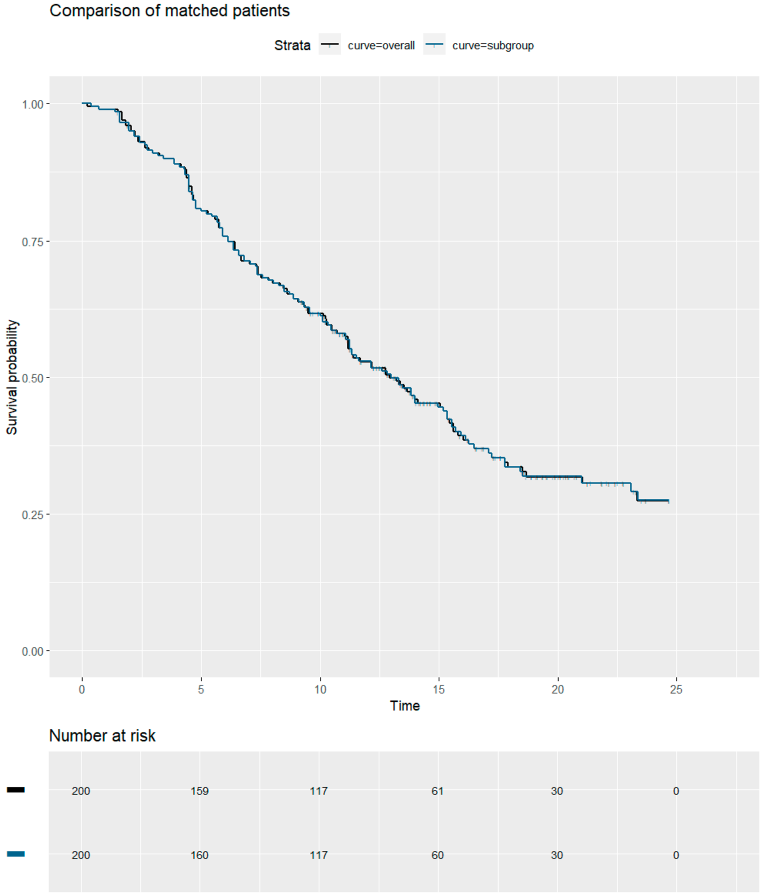

ORIENT-16 PFS PD-L1 CPS≥5 in chemotherapy group<br>Utilizing empirical cumulative distributions and Bland-Altman plots to investigate discrepancies among matched pairs	Kaplan Meier Curves and Cox-proportional hazard’s model comparing matched pairs

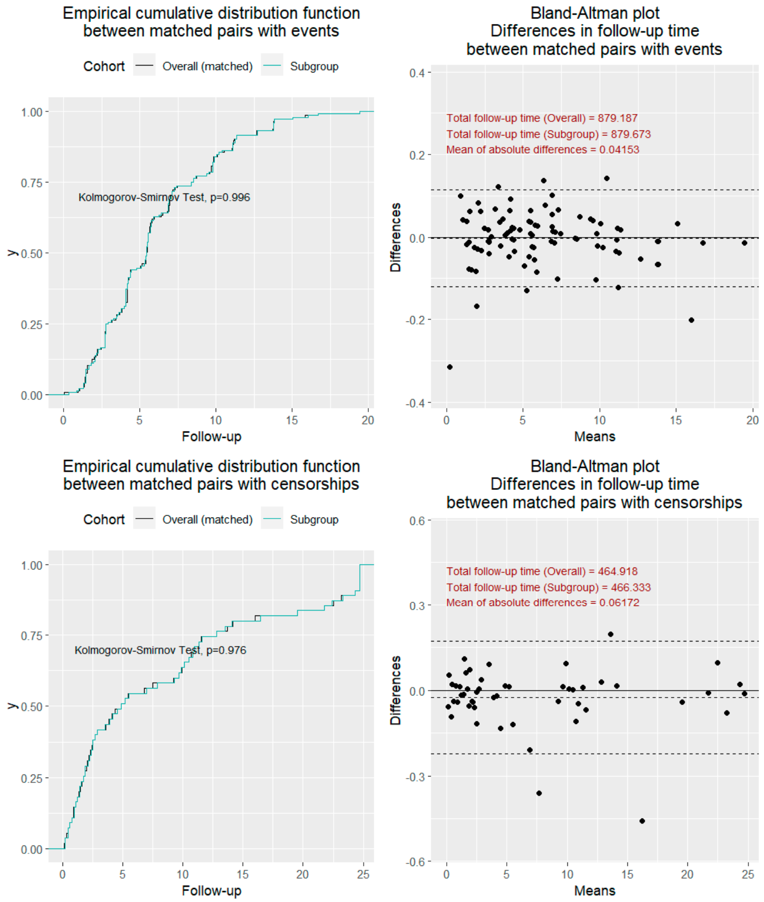

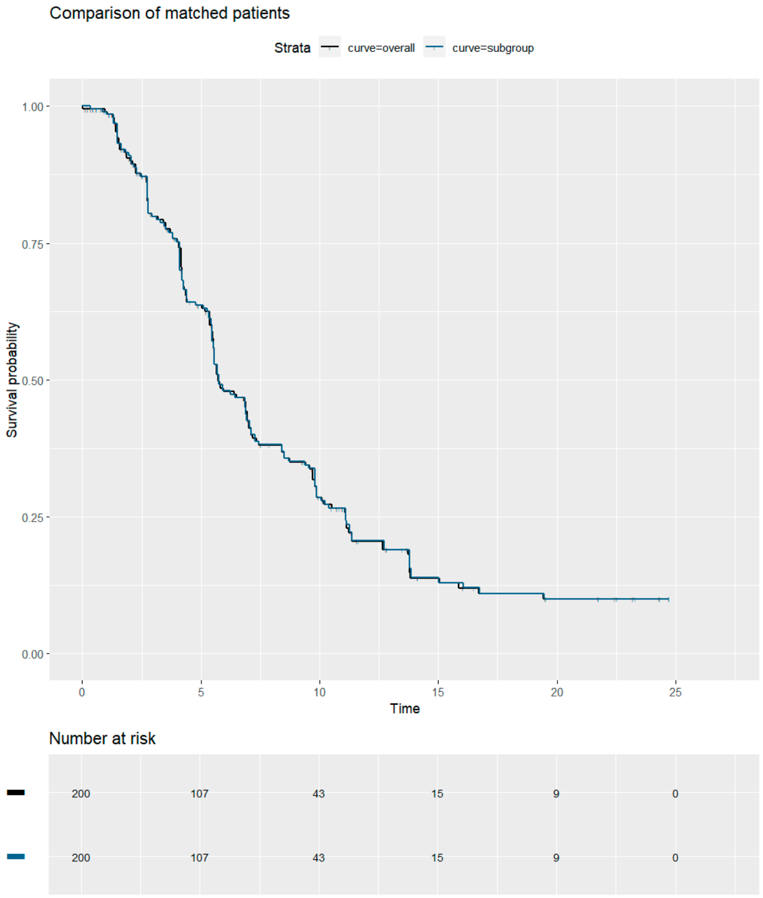

ORIENT-16 OS PD-L1 CPS≥5 in Sintilimab plus chemotherapy group Utilizing empirical cumulative distributions and Bland-Altman plots to investigate discrepancies among matched pairs	Kaplan Meier Curves and Cox-proportional hazard's model comparing matched pairs
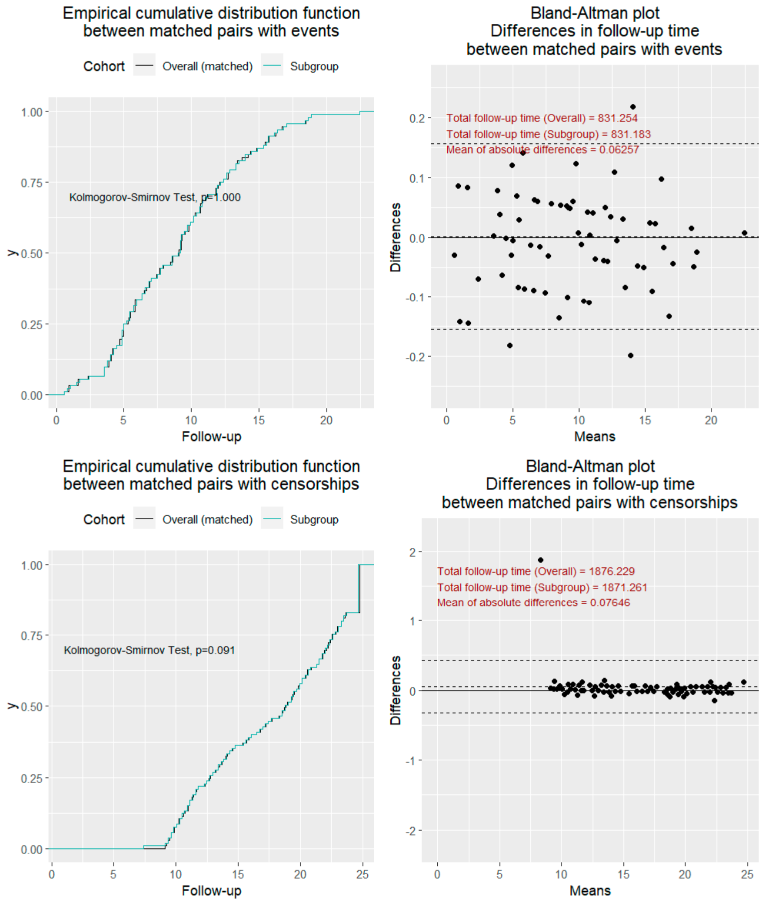	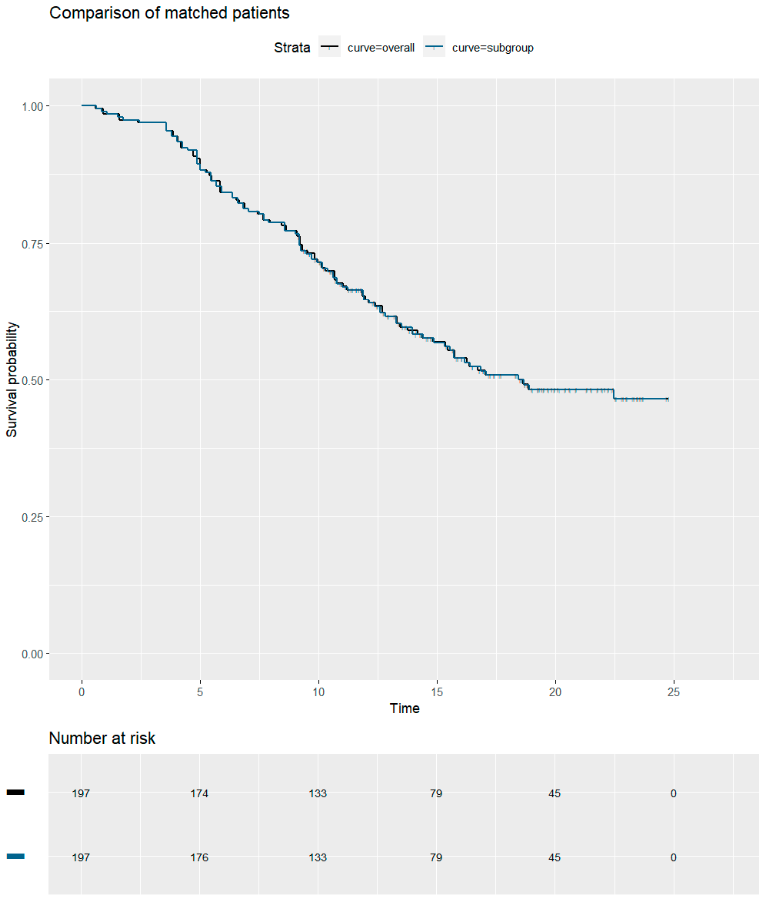

ORIENT-16 PFS PD-L1 CPS≥5 in Sintilimab plus chemotherapy group Utilizing empirical cumulative distributions and Bland-Altman plots to investigate discrepancies among matched pairs

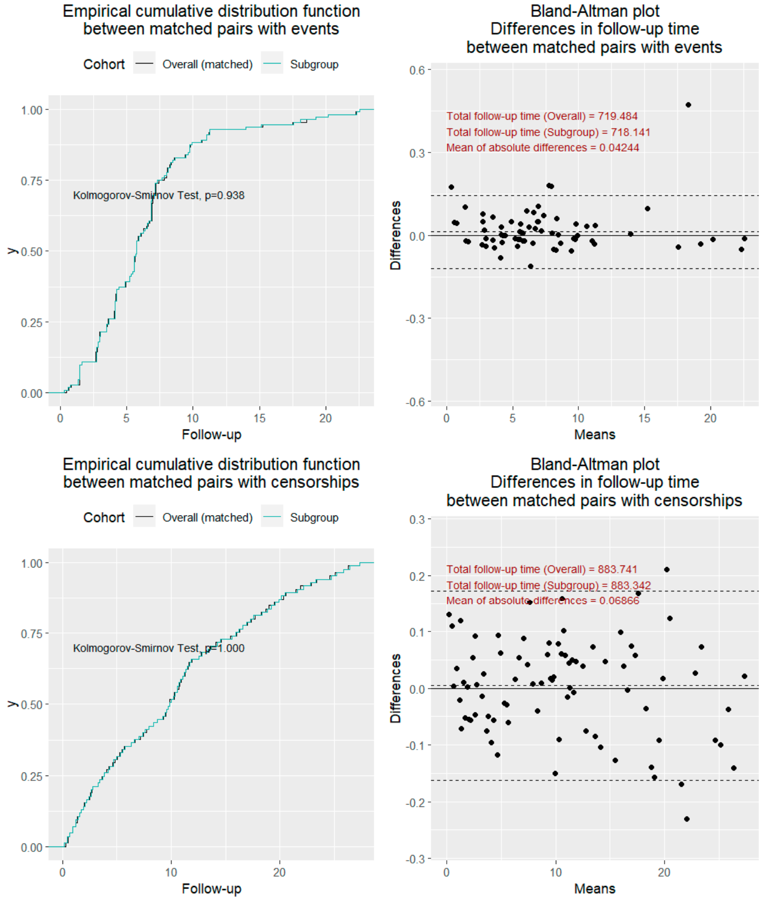

Kaplan Meier Curves and Cox-proportional hazard's model comparing matched pairs

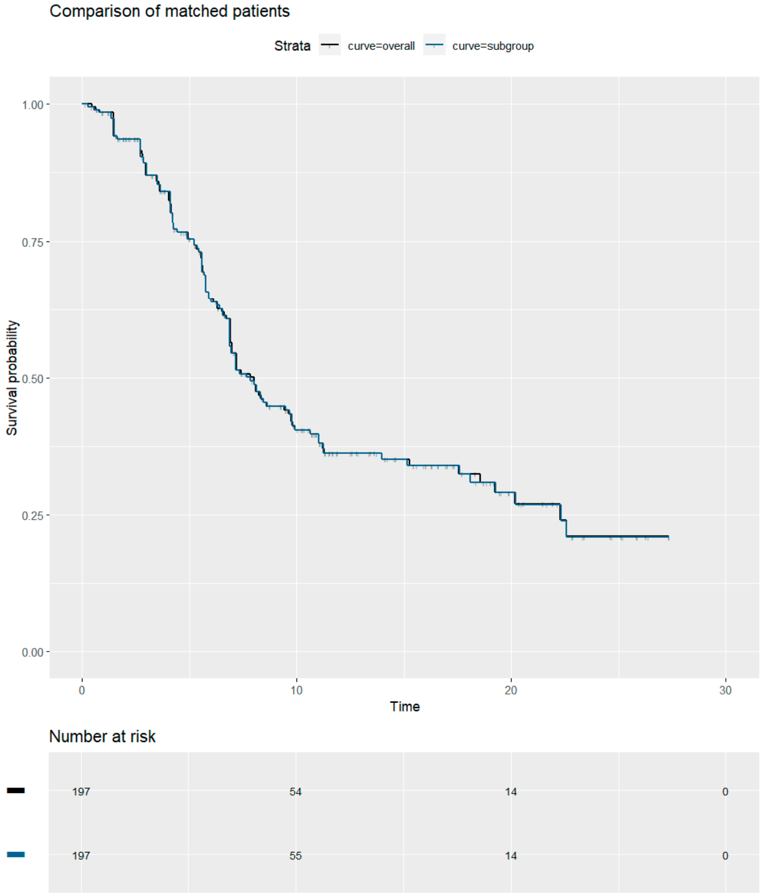

RATIONALE-305 OS PD-L1 TAP≥5% in chemotherapy group

Kaplan Meier Curves and Cox-proportional hazard's model comparing

**Utilizing empirical cumulative distributions and Bland-Altman plots to investigate discrepancies among matched pairs**	**matched pairs**

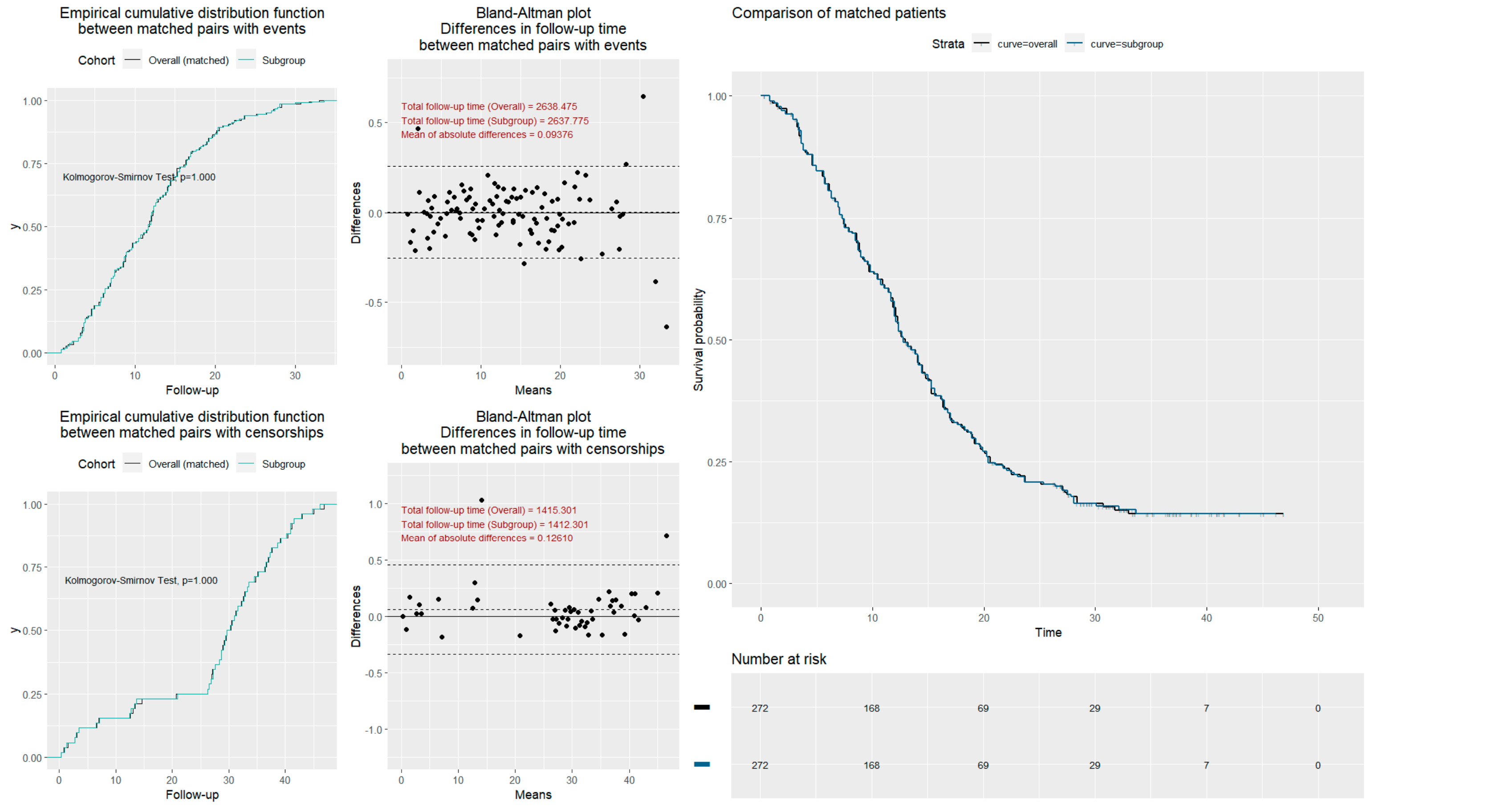

RATIONALE-305 PFS PD-L1 TAP≥5% in chemotherapy group Utilizing empirical cumulative distributions and Bland-Altman plots to investigate discrepancies among matched pairs

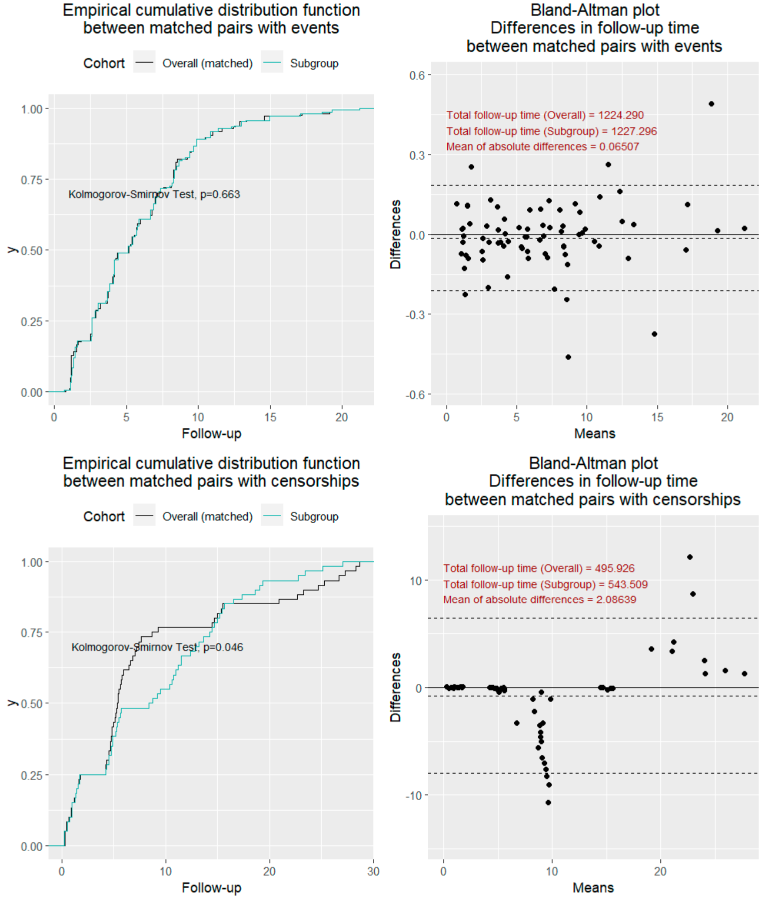

Kaplan Meier Curves and Cox-proportional hazard's model comparing matched pairs

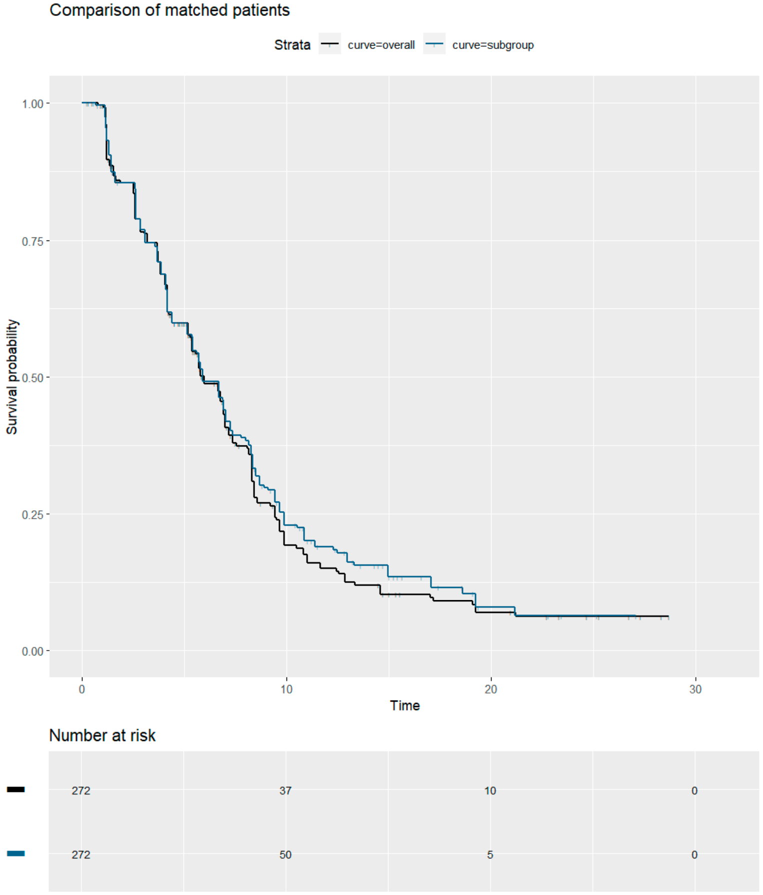

RATIONALE-305 OS PD-L1 TAP≥5% in Tislelizumab plus chemotherapy group<br>Utilizing empirical cumulative distributions and Bland-Altman plots to investigate discrepancies among matched pairs	Kaplan Meier Curves and Cox-proportional hazard's model comparing matched pairs
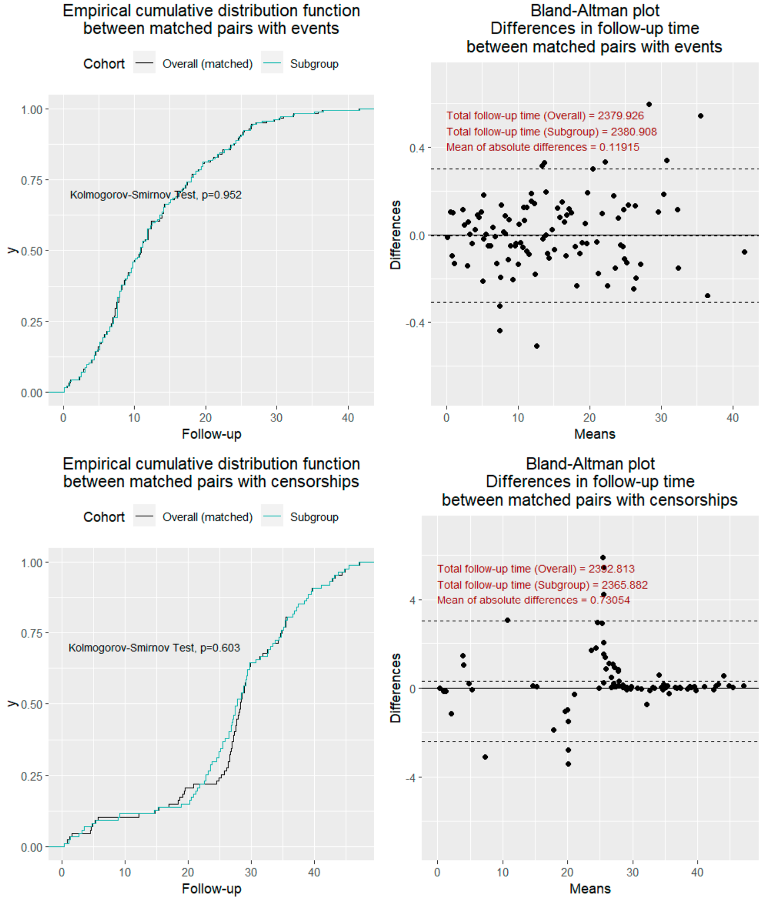	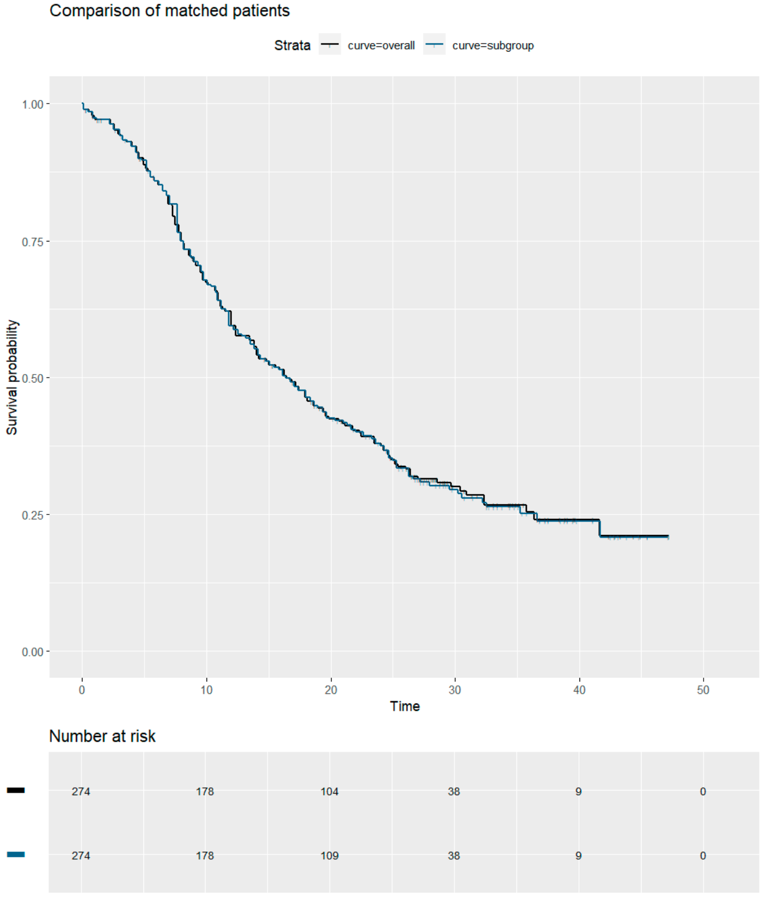

RATIONALE-305 PFS PD-L1 TAP≥5% in Tislelizumab plus chemotherapy group<br>Utilizing empirical cumulative distributions and Bland-Altman plots to investigate discrepancies among matched pairs	Kaplan Meier Curves and Cox-proportional hazard’s model comparing matched pairs

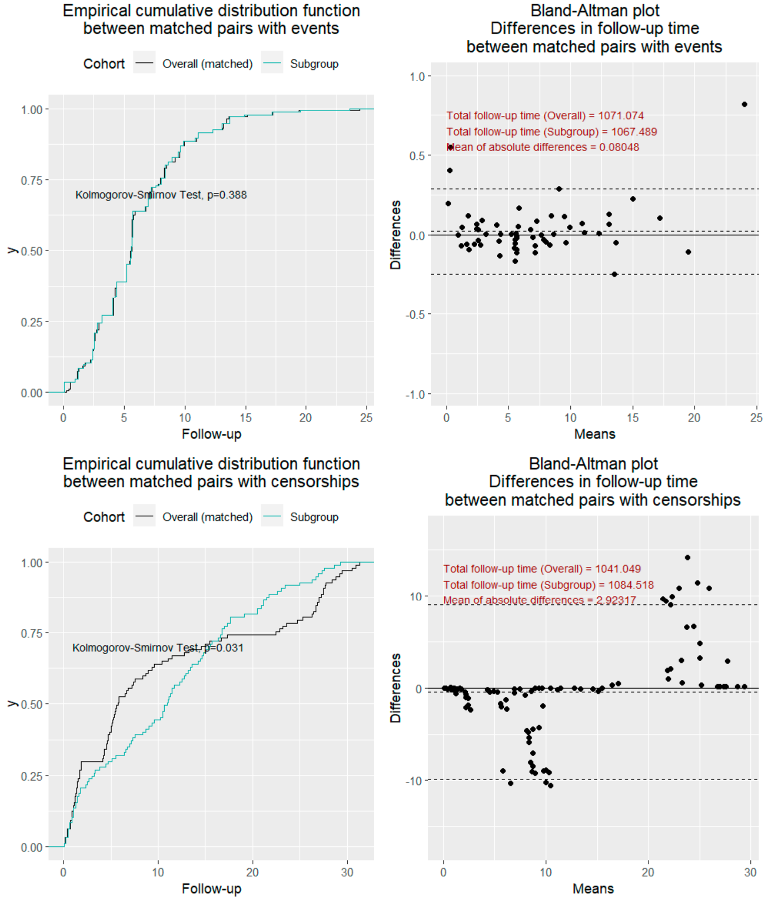

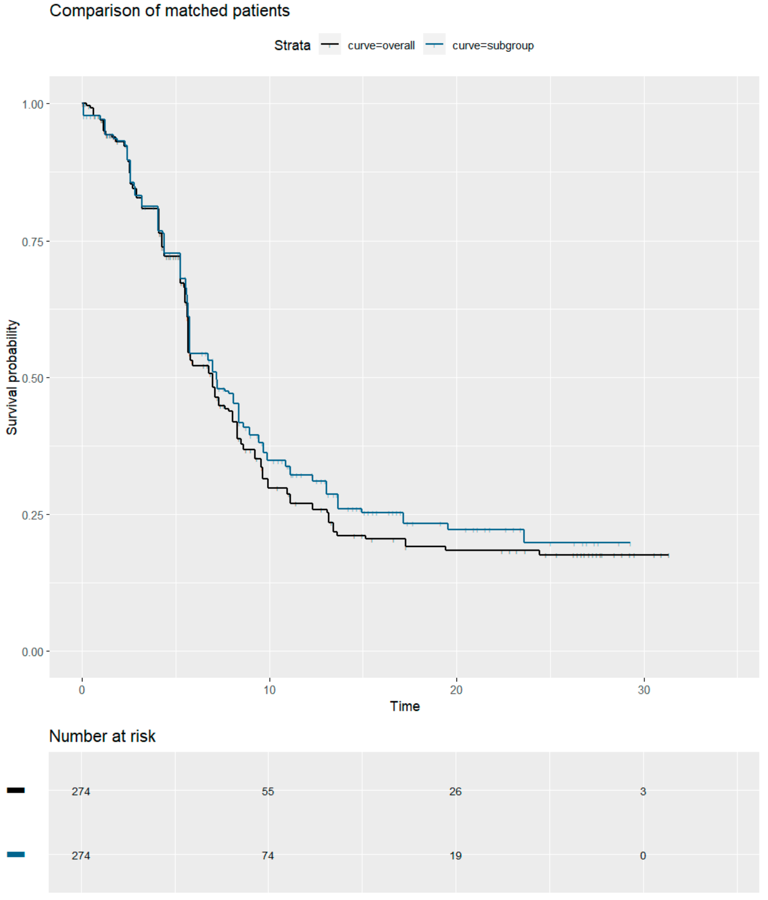

Supplementary Table S7: Simulated limits of error of KMSubtraction per implementation

Trial	Arm	Matched subgroup for the implementation of KMSubtraction	Outcome	|ln(HR)|, mean	ln(HR)|, sd
CheckMate-649	chemotherapy	PD-L1 CPS<1	OS	0.0148	0.0134
CheckMate-649	Nivolumab plus chemotherapy	PD-L1 CPS<1	OS	0.0127	0.0121
CheckMate-649	chemotherapy	PD-L1 CPS<1	PFS	0.0161	0.0131
CheckMate-649	Nivolumab plus chemotherapy	PD-L1 CPS<1	PFS	0.0146	0.0129
CheckMate-649	chemotherapy	PD-L1 CPS1-4	OS	0.0114	0.0111
CheckMate-649	Nivolumab plus chemotherapy	PD-L1 CPS1-4	OS	0.0116	0.0112
CheckMate-649	chemotherapy	PD-L1 CPS1-4	PFS	0.0114	0.0107
CheckMate-649	Nivolumab plus chemotherapy	PD-L1 CPS1-4	PFS	0.0116	0.0112
KEYNOTE-859	chemotherapy	PD-L1 CPS<1	OS	0.0111	0.0107
KEYNOTE-859	Pembrolizumab plus chemotherapy	PD-L1 CPS<1	OS	0.0111	0.0109
KEYNOTE-859	chemotherapy	PD-L1 CPS<1	PFS	0.0063	0.0063
KEYNOTE-859	Pembrolizumab plus chemotherapy	PD-L1 CPS<1	PFS	0.0071	0.0071
KEYNOTE-859	chemotherapy	PD-L1 CPS1-9	OS	0.0106	0.0104
KEYNOTE-859	Pembrolizumab plus chemotherapy	PD-L1 CPS1-9	OS	0.0109	0.0107

KEYNOTE-859	chemotherapy	PD-L1 CPS1-9	PFS	0.0063	0.0063
KEYNOTE-859	Pembrolizumab plus chemotherapy	PD-L1 CPS1-9	PFS	0.0067	0.0067
ORIENT-16	chemotherapy	PD-L1 CPS<5	OS	0.0145	0.0116
ORIENT-16	Sintilimab plus chemotherapy	PD-L1 CPS<5	OS	0.0148	0.0116
ORIENT-16	chemotherapy	PD-L1 CPS<5	PFS	0.0139	0.0127
ORIENT-16	Sintilimab plus chemotherapy	PD-L1 CPS<5	PFS	0.0151	0.0118
RATIONALE-305	chemotherapy	PD-L1 TAP<5%	OS	0.0090	0.0086
RATIONALE-305	Tislelizumab plus chemotherapy	PD-L1 TAP<5%	OS	0.0092	0.0088
RATIONALE-305	chemotherapy	PD-L1 TAP<5%	PFS	0.0084	0.0082
RATIONALE-305	Tislelizumab plus chemotherapy	PD-L1 TAP<5%	PFS	0.0088	0.0087

Supplementary Table S8: Convergence Plots and Histograms of Simulations

<table>
<tr><th>Histogram of ln (HR)</th><th>Histogram of |ln (HR)|</th><th>Convergence plot</th></tr>
<tr><td colspan="3">CheckMate-649 OS PD-L1 CPS<1 in chemotherapy</td></tr>
<tr><td>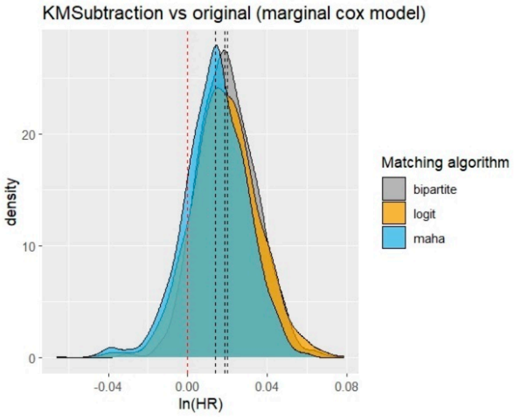
</td><td>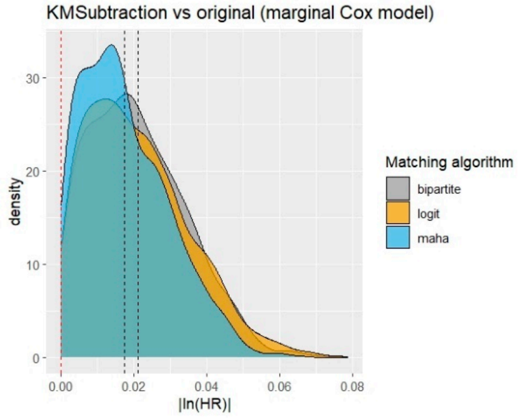
</td><td>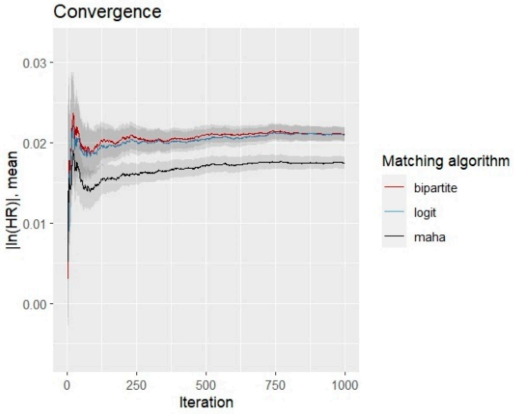
</td></tr>
<tr><td colspan="3">CheckMate-649 OS PD-L1 CPS<1 in Nivolumab plus chemotherapy</td></tr>
<tr><td>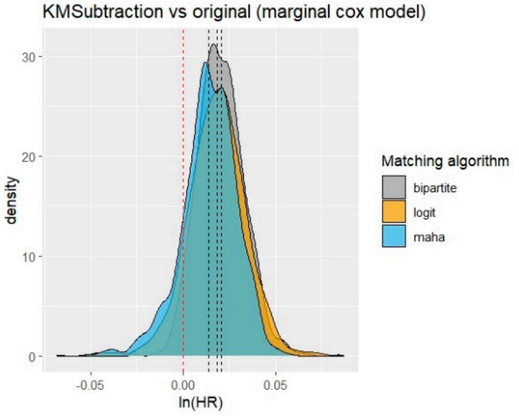
</td><td>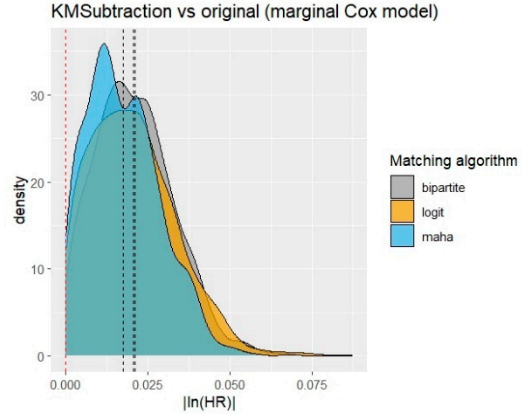
</td><td>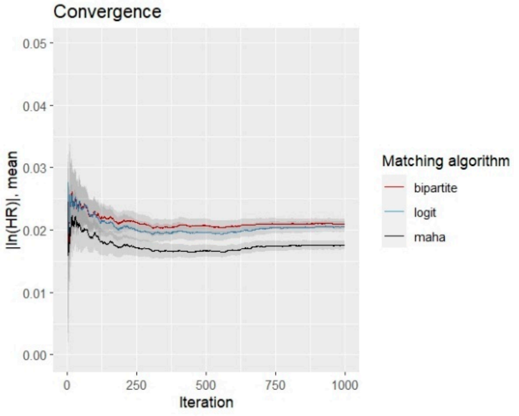
</td></tr>
</table>

CheckMate-649 PFS PD-L1 CPS<1 in chemotherapy

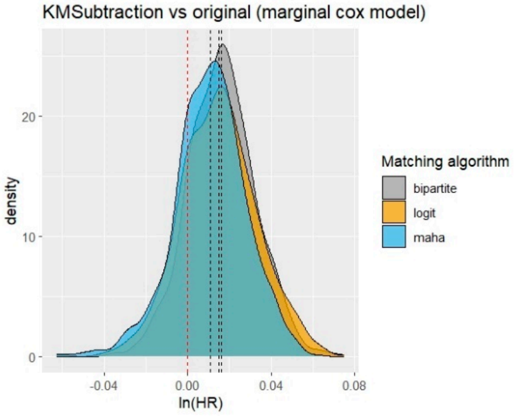

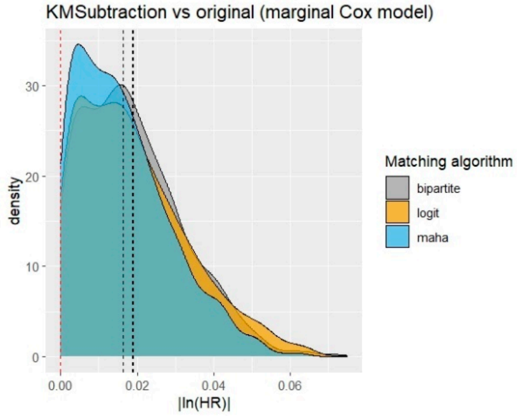

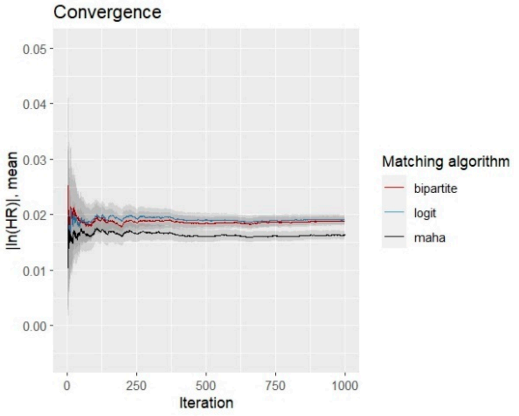

CheckMate-649 PFS PD-L1 CPS<1 in Nivolumab plus chemotherapy

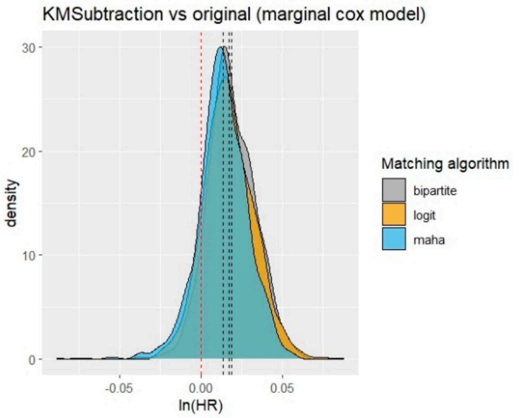

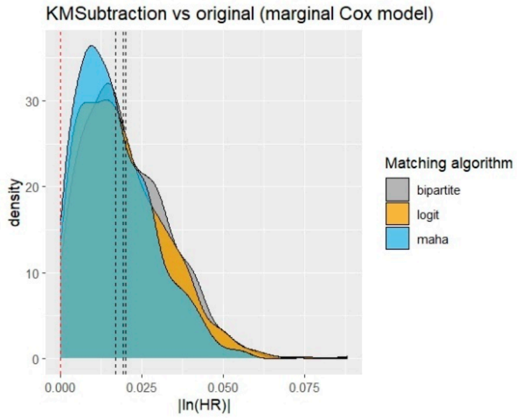

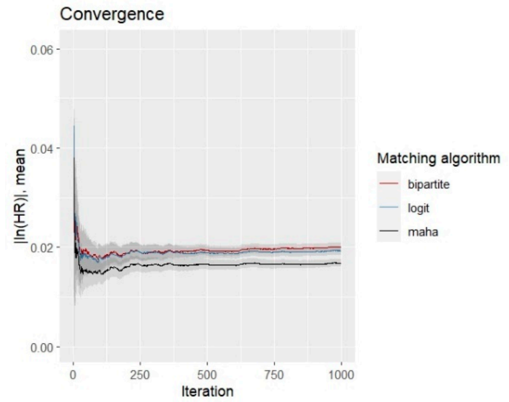

CheckMate-649 OS PD-L1 CPS1-4 in chemotherapy

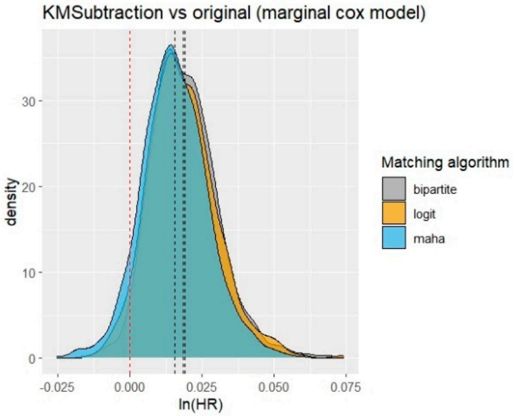

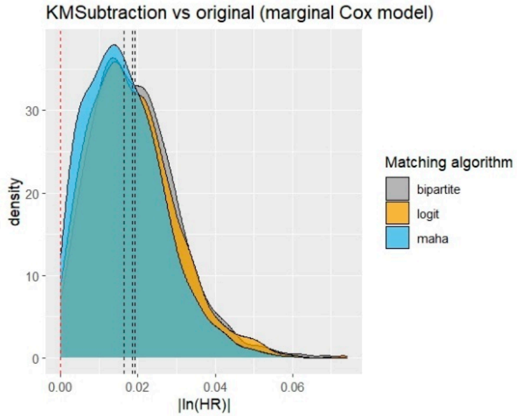

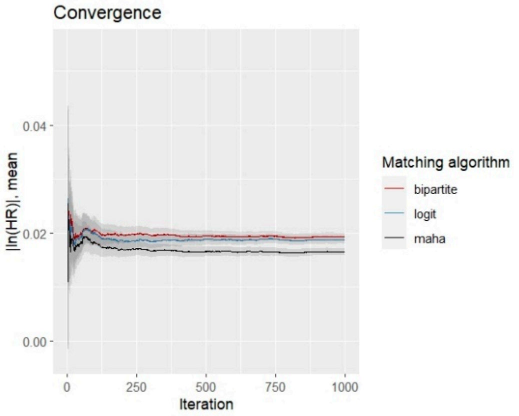

CheckMate-649 OS PD-L1 CPS1-4 in Nivolumab plus chemotherapy

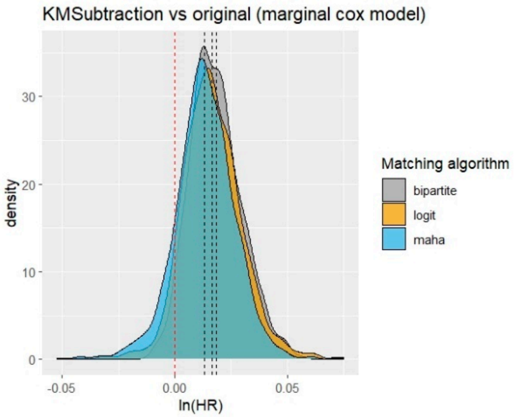

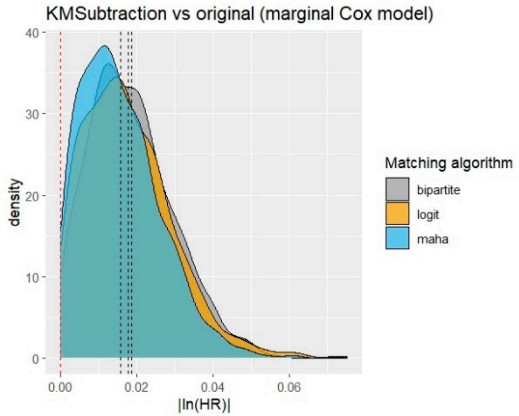

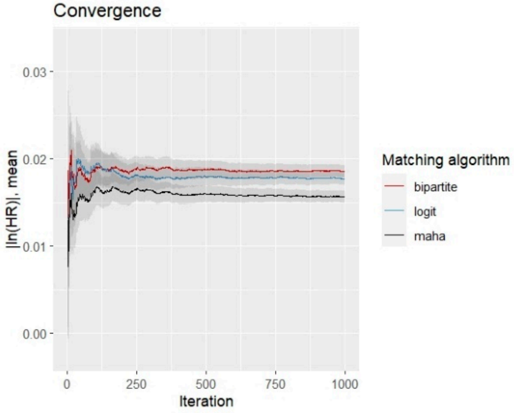

CheckMate-649 PFS PD-L1 CPS1-4 in chemotherapy

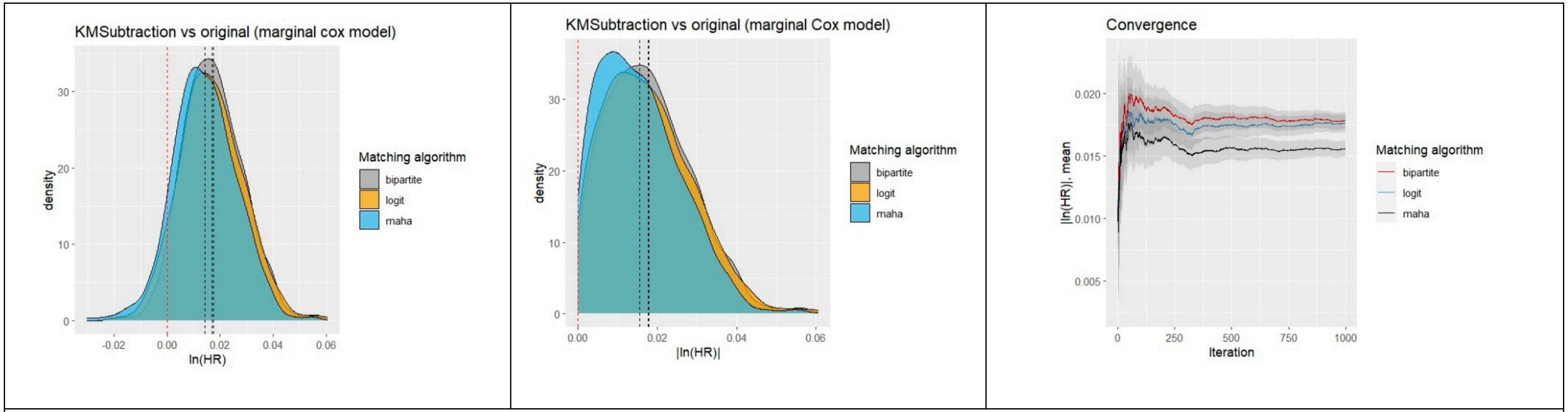

CheckMate-649 PFS PD-L1 CPS1-4 in Nivolumab plus chemotherapy

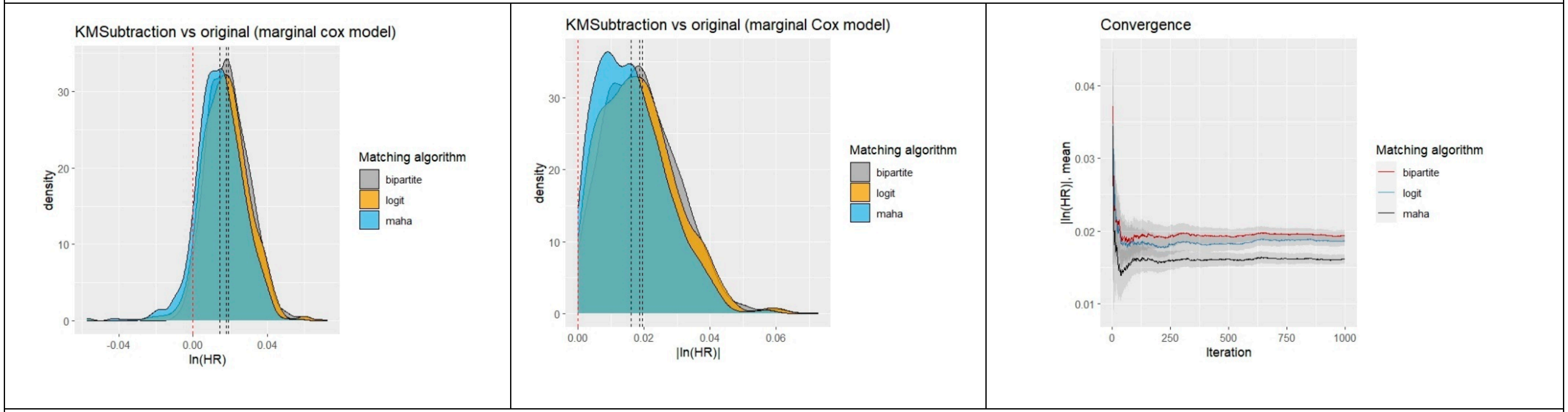

KEYNOTE-859 OS PD-L1 CPS<1 in chemotherapy

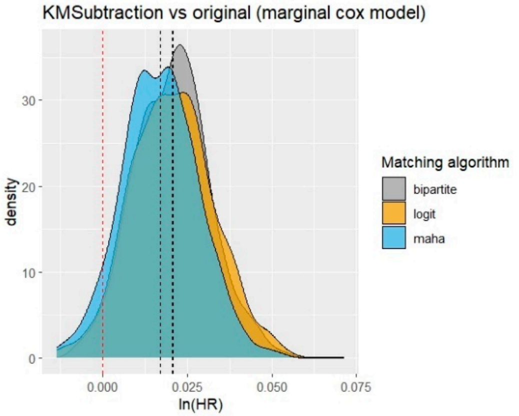

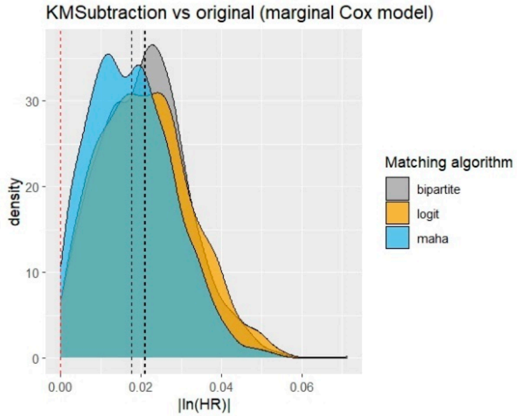

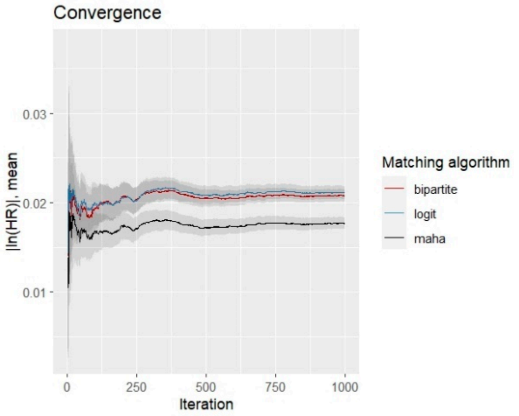

KEYNOTE-859 OS PD-L1 CPS<1 in Pembrolizumab plus chemotherapy

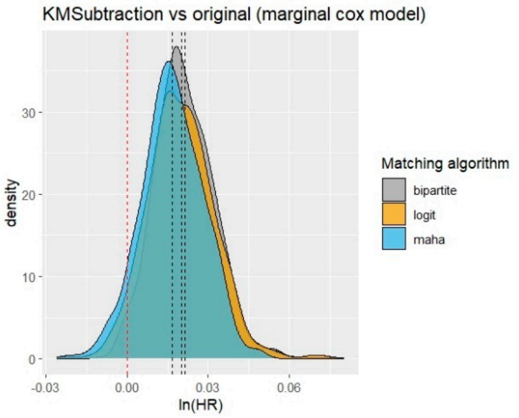

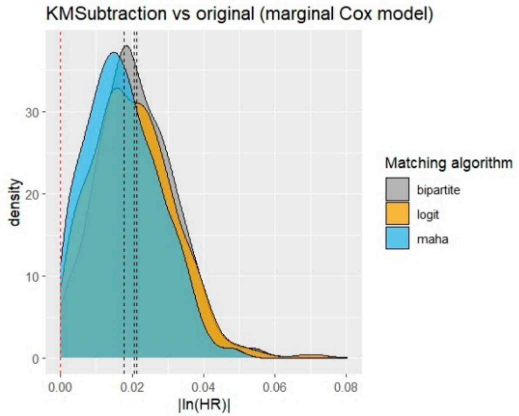

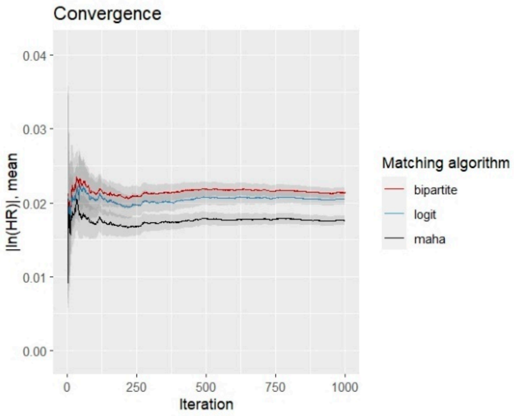

KEYNOTE-859 PFS PD-L1 CPS<1 in chemotherapy

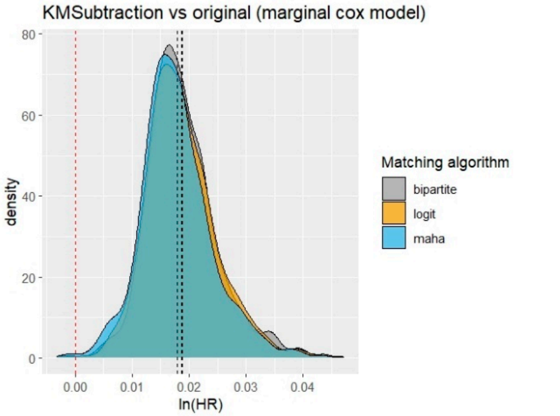

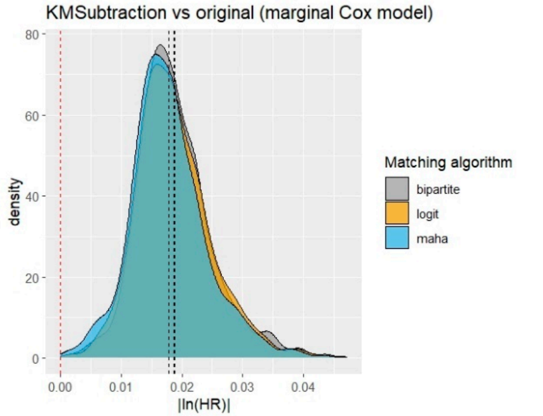

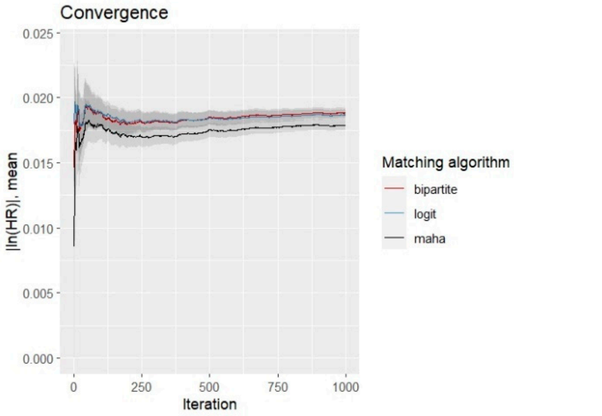

KEYNOTE-859 PFS PD-L1 CPS<1 in Pembrolizumab plus chemotherapy

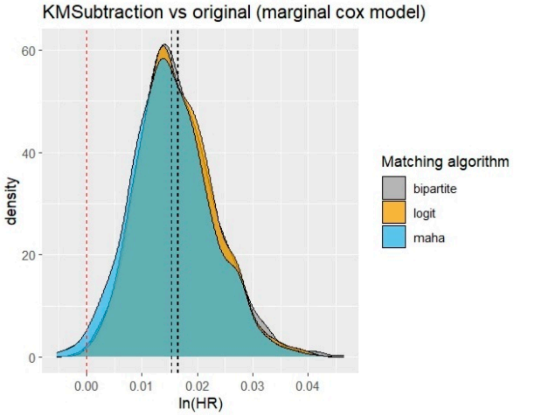

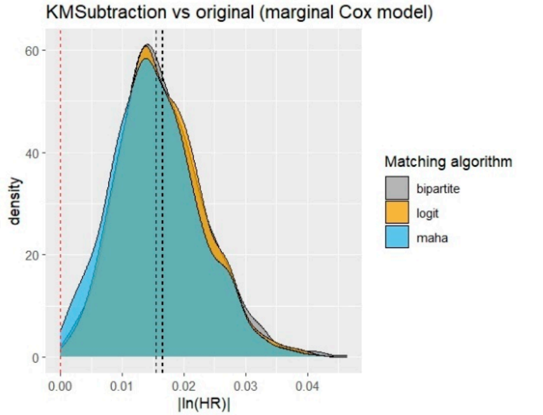

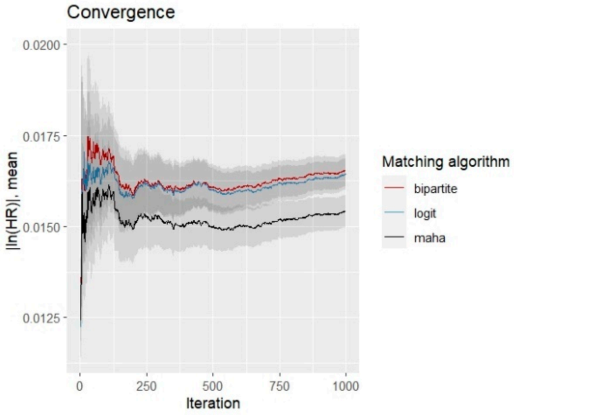

KEYNOTE-859 OS PD-L1 CPS1-9 in chemotherapy

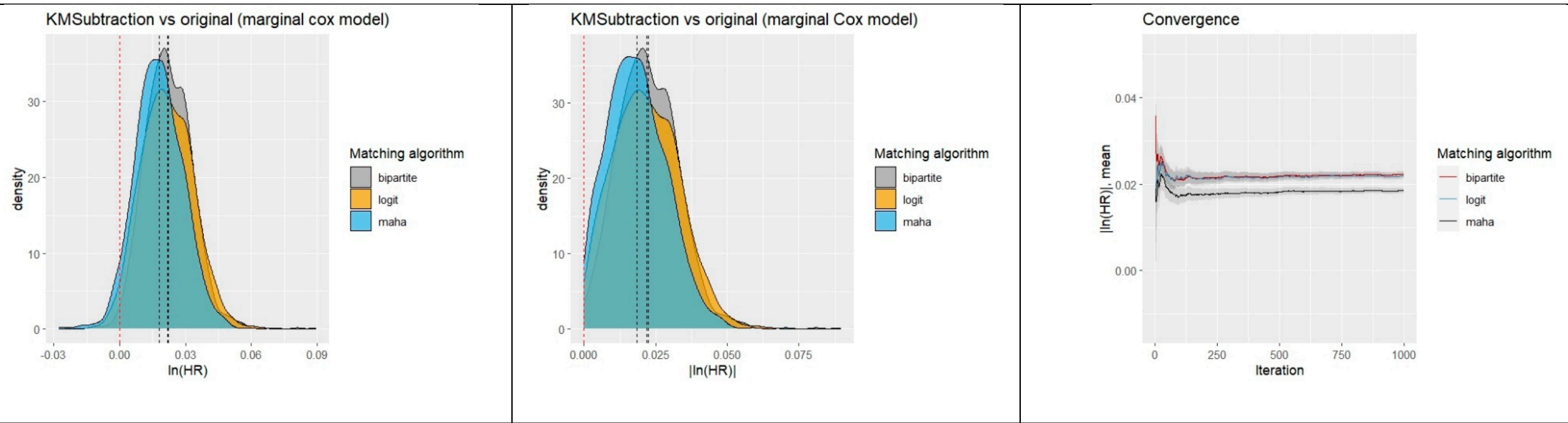

KEYNOTE-859 OS PD-L1 CPS1-9 in Pembrolizumab plus chemotherapy

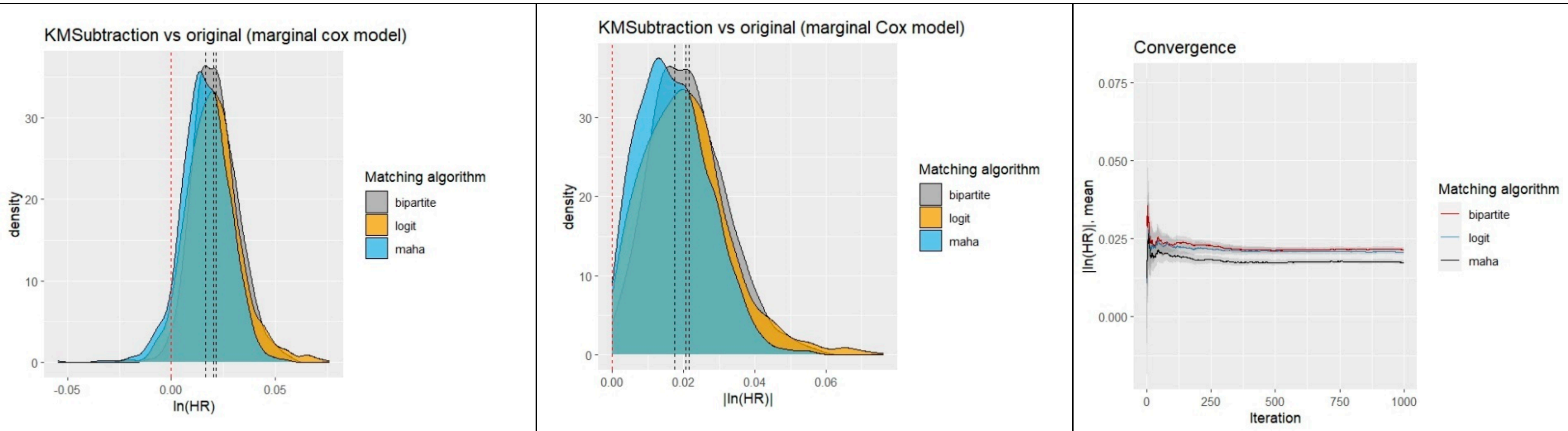

KEYNOTE-859 PFS PD-L1 CPS1-9 in chemotherapy

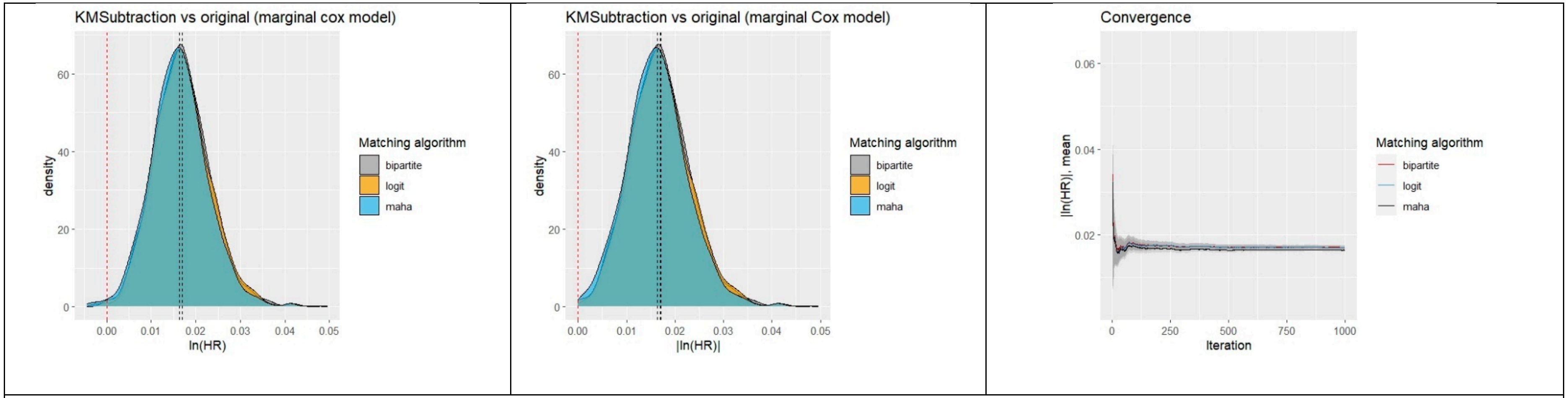

KEYNOTE-859 PFS PD-L1 CPS1-9 in Pembrolizumab plus chemotherapy

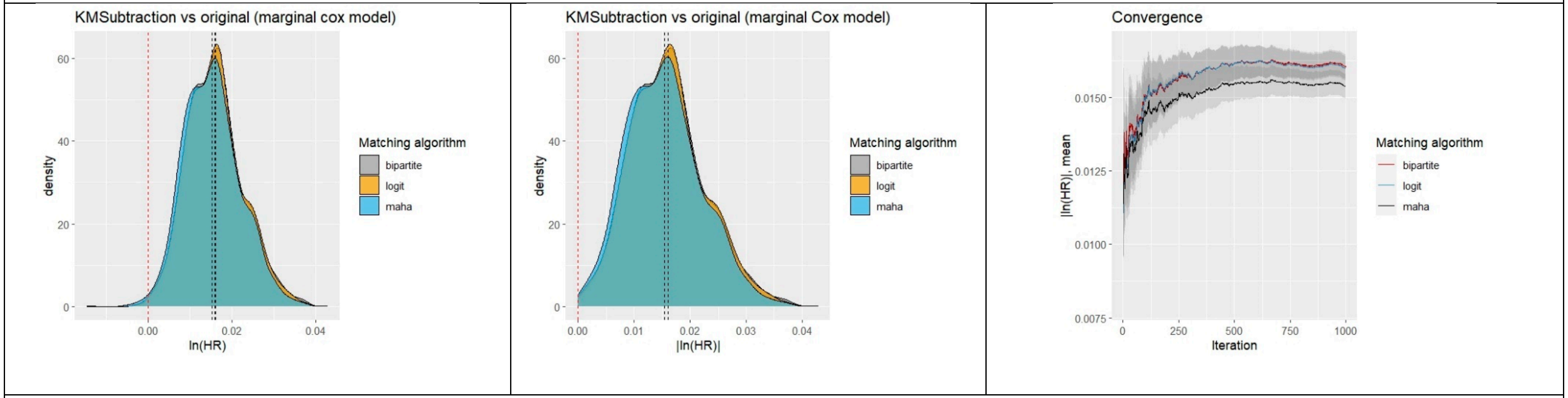

ORIENT-16 OS PD-L1 CPS<5 in chemotherapy

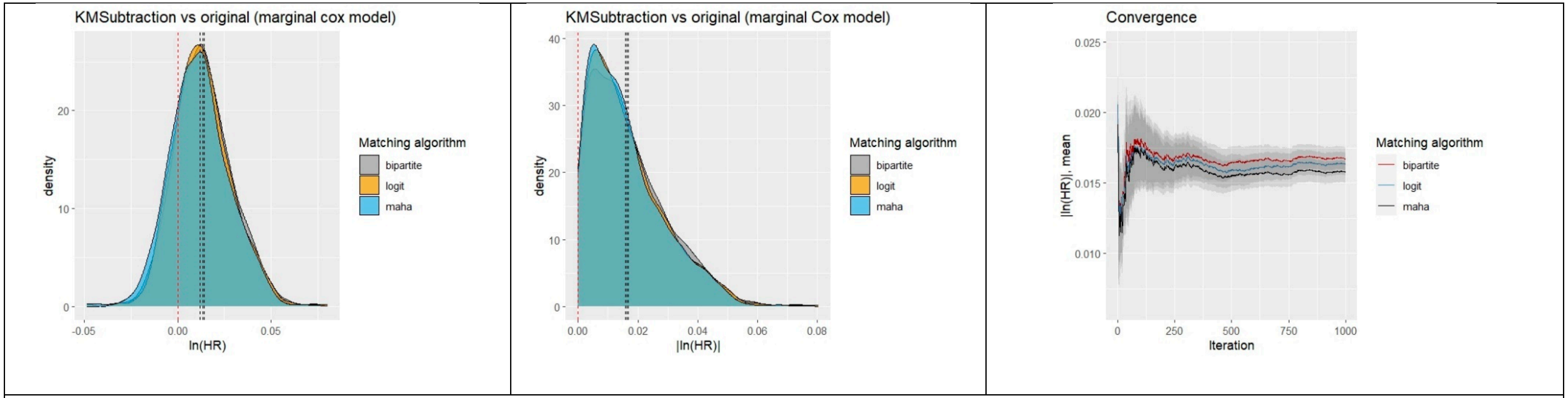

ORIENT-16 OS PD-L1 CPS<5 in Sintilimab plus chemotherapy

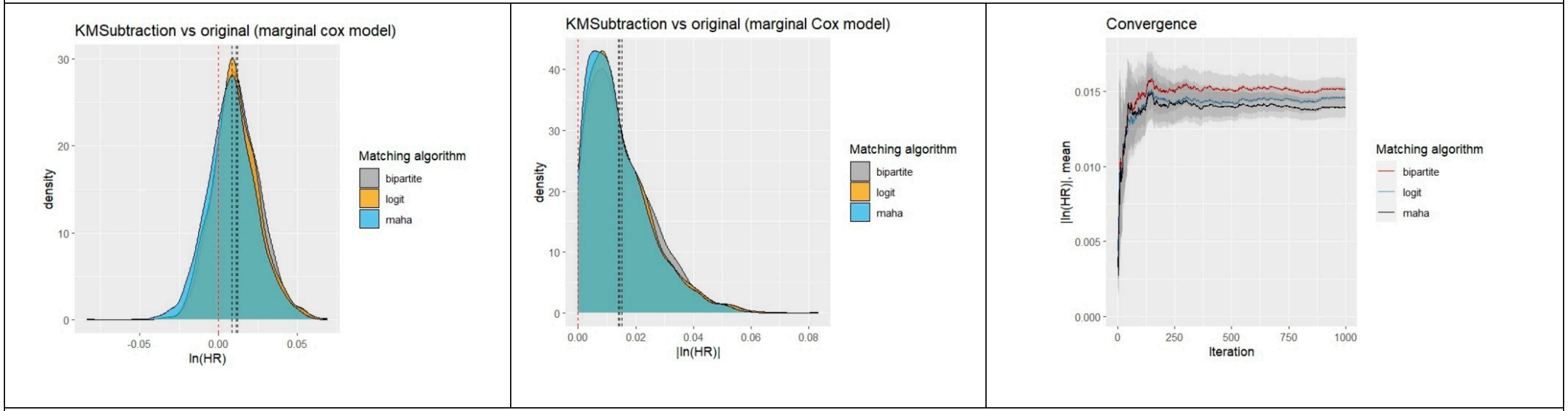

ORIENT-16 PFS PD-L1 CPS<5 in chemotherapy

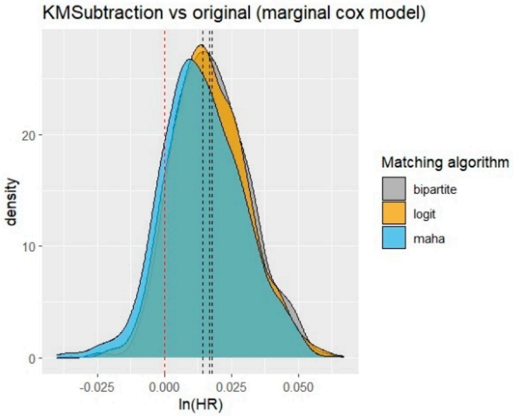

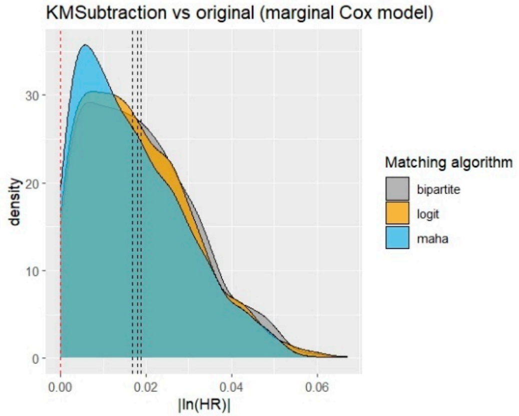

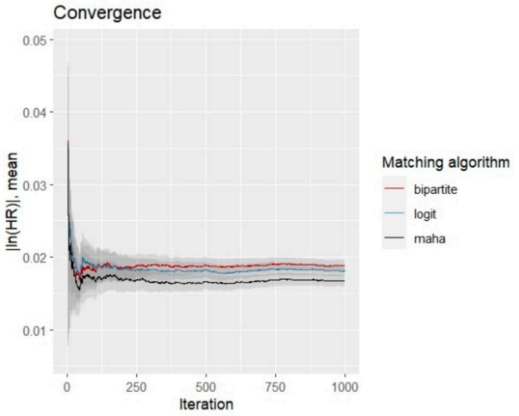

ORIENT-16 PFS PD-L1 CPS<5 in Sintilimab plus chemotherapy

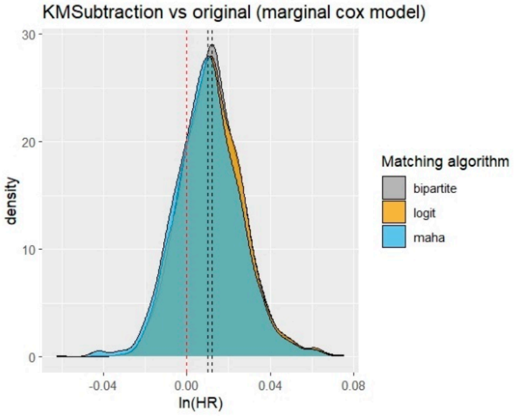

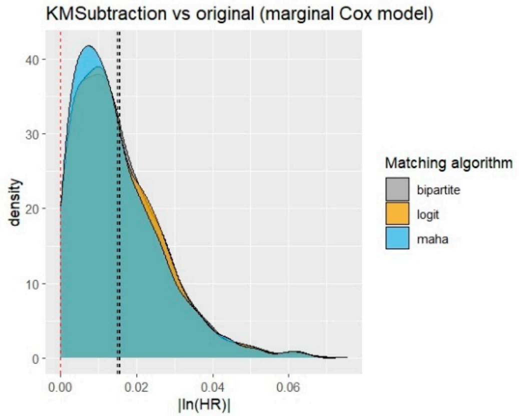

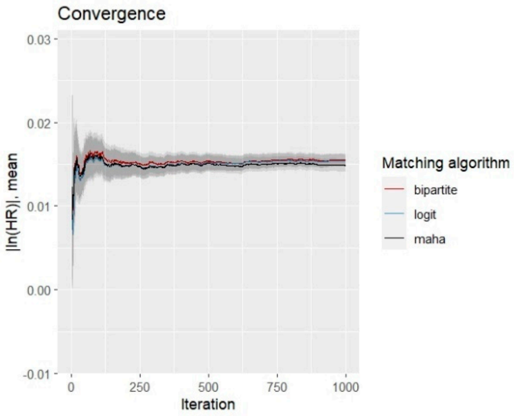

CheckMate-649 OS PD-L1 CPS<1 in Nivolumab plus chemotherapy

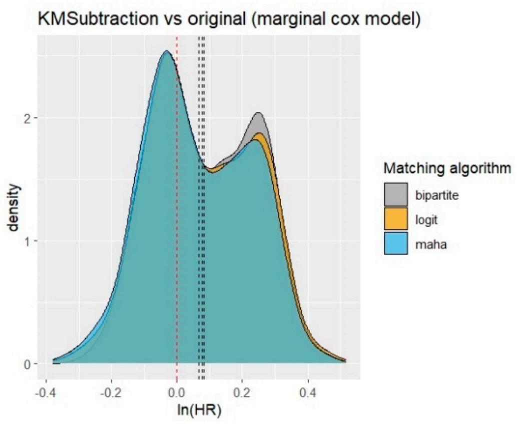

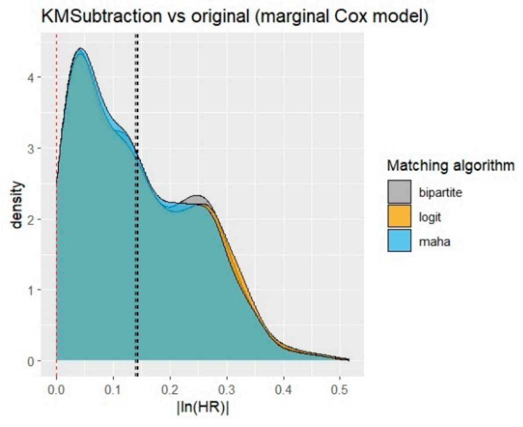

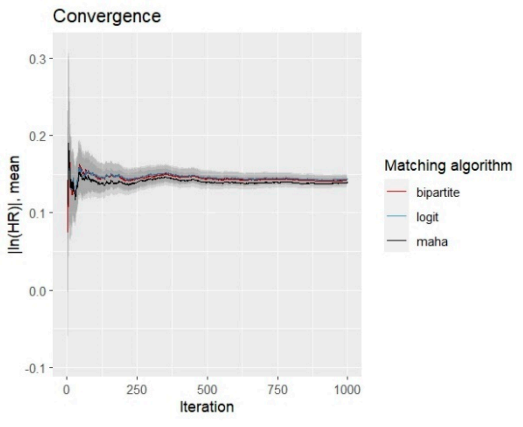

CheckMate-649 PFS PD-L1 CPS<1 in Nivolumab plus chemotherapy

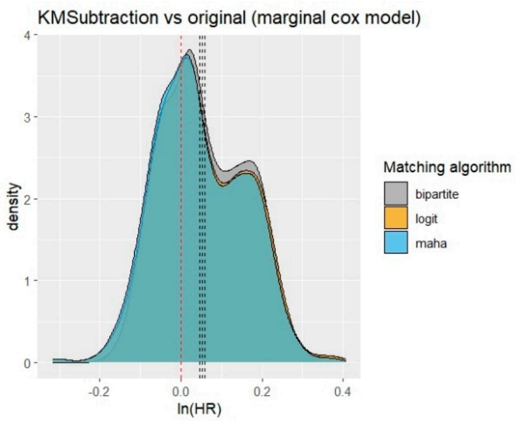

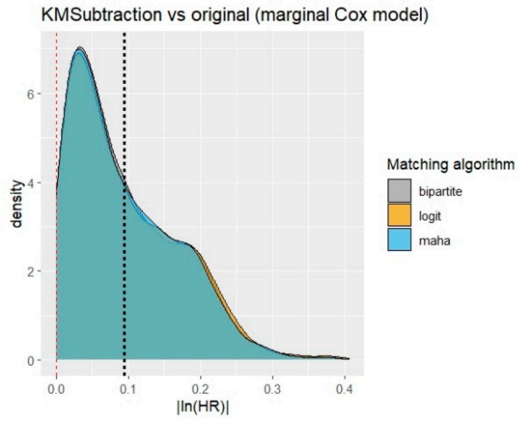

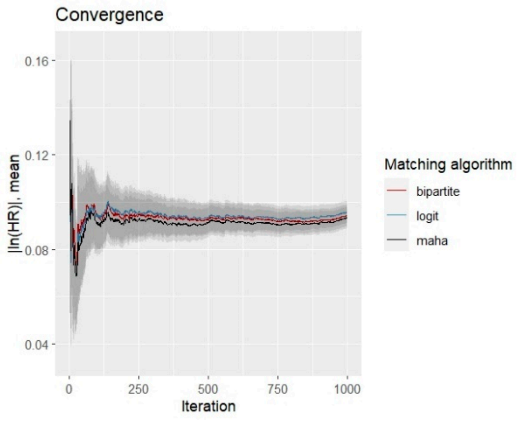

CheckMate-649 OS PD-L1 CPS1-4 in Nivolumab plus chemotherapy

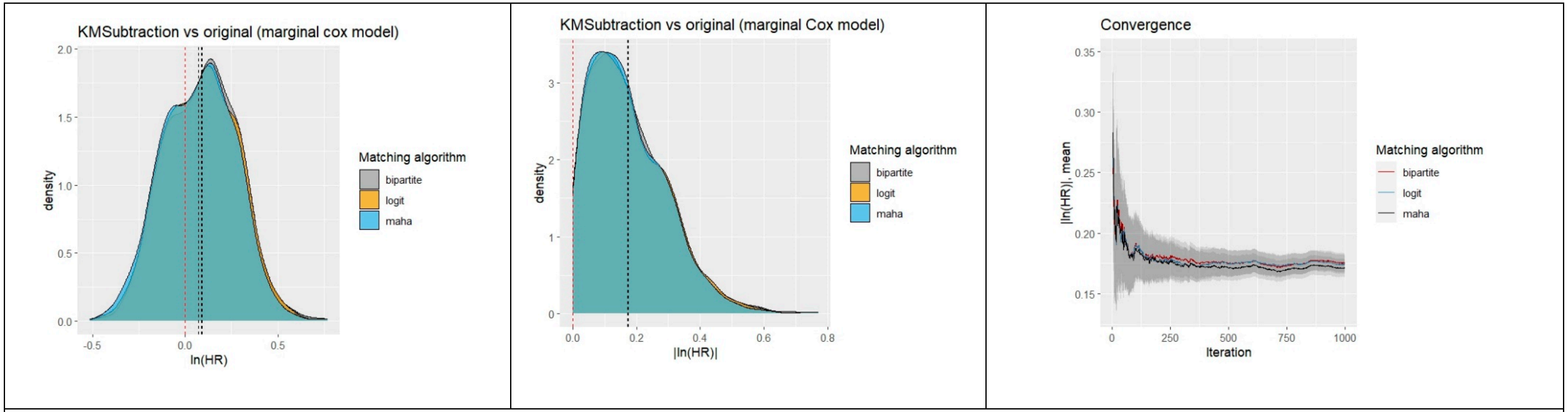

CheckMate-649 PFS PD-L1 CPS1-4 in Nivolumab plus chemotherapy

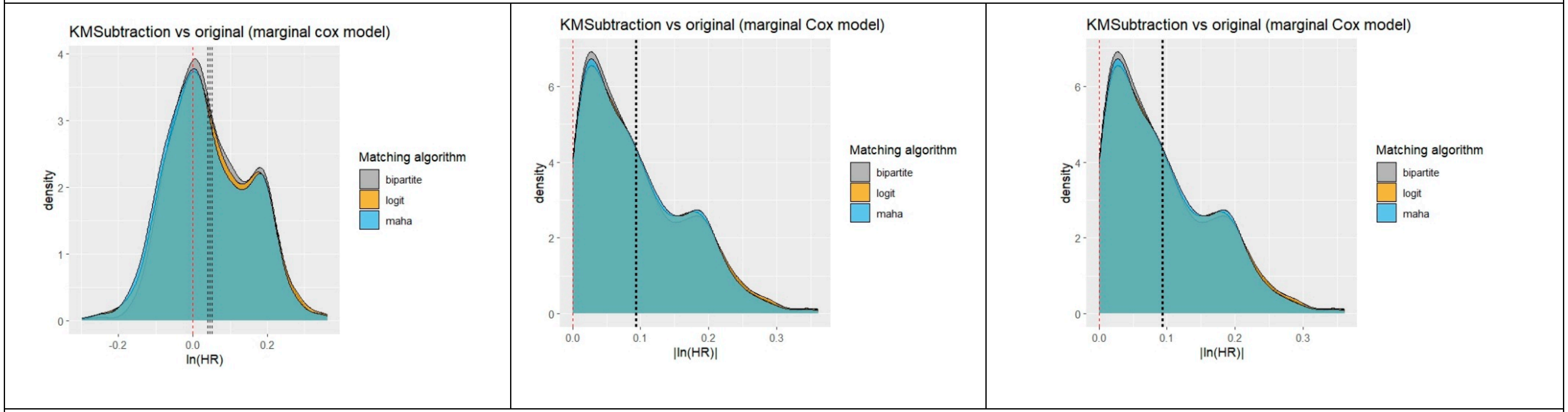

RATIONALE-305 OS PD-L1 TAP<5% in chemotherapy

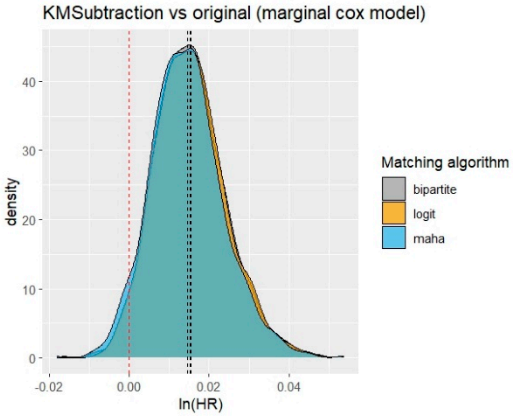

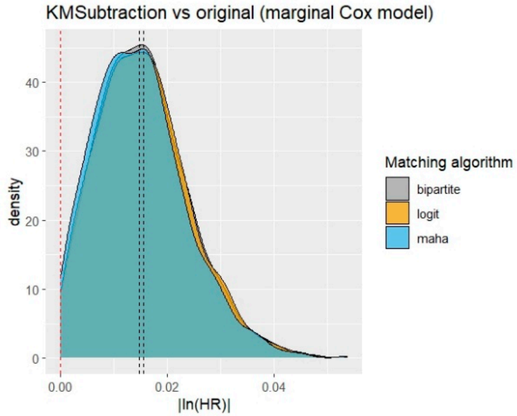

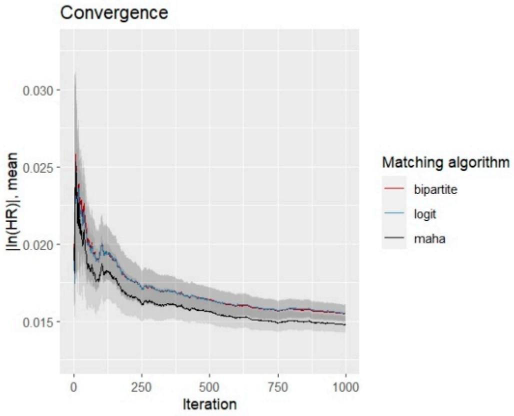

RATIONALE-305 OS PD-L1 TAP<5% in Tislelizumab plus chemotherapy

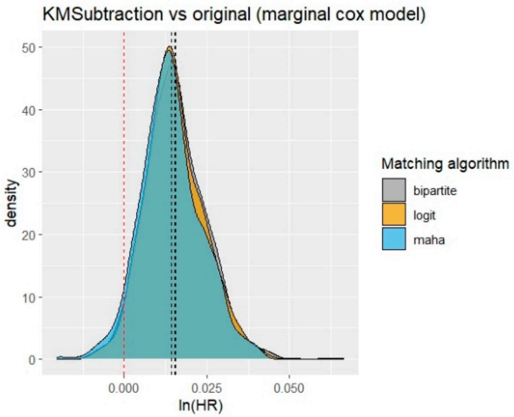

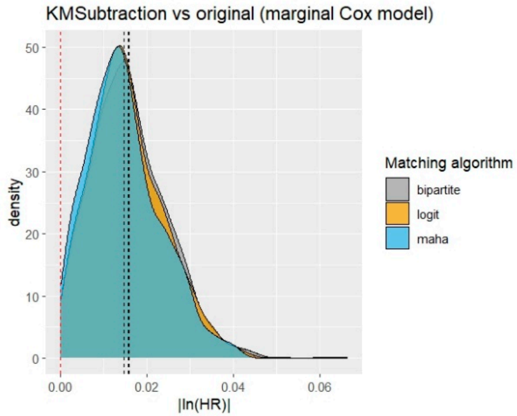

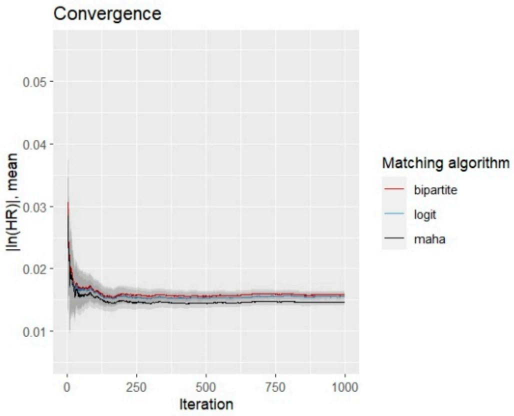

RATIONALE-305 PFS PD-L1 TAP<5% in chemotherapy

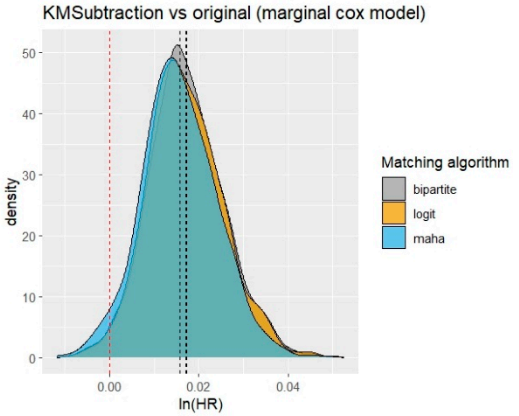

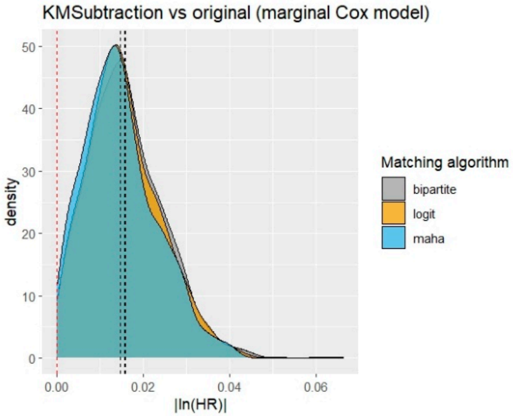

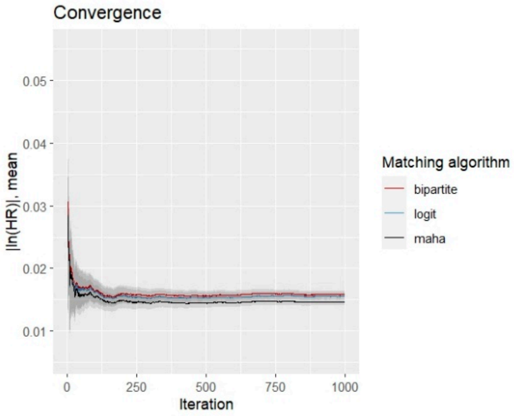

RATIONALE-305 PFS PD-L1 TAP<5% in Tislelizumab plus chemotherapy

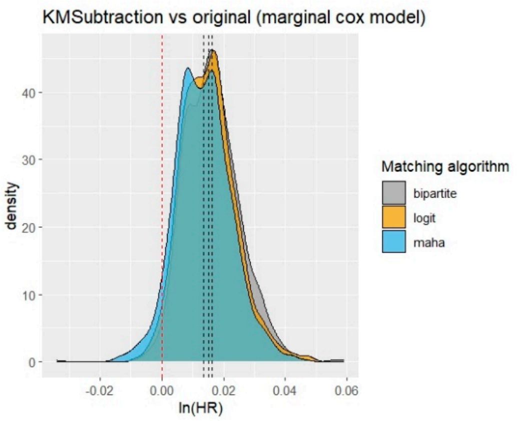

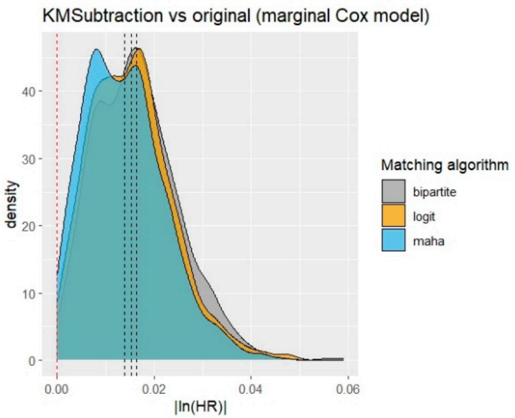

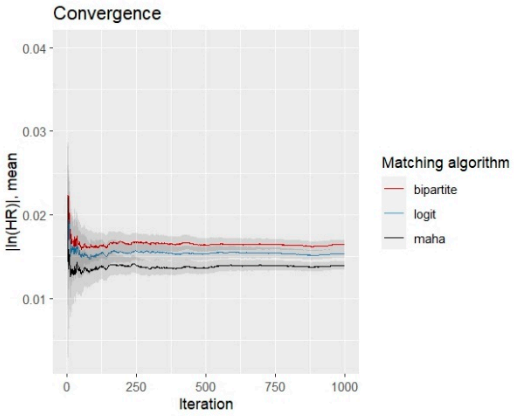

Supplementary Table S9: Treatment-related adverse events

	TRAEsa											
**Trial**	**ICI-chemo**						**Chemo**					
**Continued-chemo**	**Patients**	**All grades**	**Grade 3-5**	**Serious events**	**leading to discontinuation**	**leading to death**	**Patients**	**All grades**	**Grade 3-5**	**Serious events**	**leading to discontinuation**	**leading to death**
CheckMate-649	782	738(95%)	462(59%)	172(22%)	284(36%)	16 (2%)	767	679(89%)	341(44%)	93(12%)	182(24%)	4(<1%)
KEYNOTE-859	785	776(99%)	466(59%)	184(23%)	207(26%)	64(8%)	787	771(98%)	402(51%)	146(19%)	158(20%)	58(7%)
**Total**	**1567**	**1514(97%)**	**928(59%)**	**356(23%)**	**491(31%）**	**80(5%)**	**1554**	**1450(93%)**	**743(48%)**	**239(15%)**	**340(22%)**	**62(4%)**
**Single-agent maintenance**												
ORIENT-16	328	319(97.3%)	196(59.8%)	151(46%)	52(16%)	6(1.8%)	320	308(96.3%)	168(52.5%)	125(39.1)	30(9%)	2(<1%)
RATIONALE-305	498	483(97%)	268(54%)	113(23%)	80(16%)	6(1%)	494	476(96%)	246(50%)	72(15%)	40(8%)	2(<1%)
**Total**	**826**	**802(97%)**	**464(56%)**	**264(32%)**	**132(16%)**	**12(1%)**	**814**	**784(96%)**	**414(51%)**	**197(24%)**	**70(9%)**	**4(<1%)**

Supplementary Table S10: Subgroup analysis by PD-L1 CPS subpopulations

Region	Trial	Total	ICI-chemo	Chemo
**Global patients**				
**≥1**	CheckMate-649	1296(82%)	641	655
	KEYNOTE-859	1235(72%)	618	617
	ORIENT-16	546(84%)	275	271
**<1**	CheckMate-649	265(17%)	140	125
	KEYNOTE-859	344(22%)	172	172
	ORIENT-16	104(16%)	52	52
**≥5**	CheckMate-649	955(60%)	473	482
	ORIENT-16	397(61%)	197	200
	RATIONALE-305	523(52%)	254	269
**<5**	CheckMate-649	606(38%)	308	298
	ORIENT-16	253(39%)	130	123
	RATIONALE-305	451(46%)	237	214
**≥10**	KEYNOTE-859	551(35%)	279	272
	ORIENT-16	288(44%)	146	142
**<10**	KEYNOTE-859	1026(65%)	509	517
	ORIENT-16	362(56%)	181	181
**Asian patients**				
**≥1**	KEYNOTE-859	401(25%)	201	200
	ORIENT-16	546(84%)	275	271
**<1**	ORIENT-16	104(16%)	52	52
**≥5**	CheckMate-649	228(14%)	117	111
	ORIENT-16	397(61%)	197	200
**<5**	ORIENT-16	253(39%)	130	123
**≥10**	KEYNOTE-859	184(12%)	96	88
	ORIENT-16	288(44%)	146	142
**<10**	ORIENT-16	362(56%)	181	181
**Chinese patients**				
**≥1**	CheckMate-649	183(11.6%)	89	94
	ORIENT-16	546(84%)	275	271
**<1**	CheckMate-649	25(1.5%)	10	15
	ORIENT-16	104(16%)	52	52
**≥5**	CheckMate-649	156(9.9%)	75	81
	ORIENT-16	397(61%)	197	200
**<5**	CheckMate-649	52(3.3%)	24	28
	ORIENT-16	253(39%)	130	123
**≥10**	ORIENT-16	288(44%)	146	142
**<10**	ORIENT-16	362(56%)	181	181
